# Organization and evolution of Gorilla centromeric DNA from old strategies to new approaches

**DOI:** 10.1038/srep14189

**Published:** 2015-09-21

**Authors:** C. R. Catacchio, R. Ragone, G. Chiatante, M. Ventura

**Affiliations:** 1 University of Bari Aldo Moro, Department of Biology, Via Orabona 4, Bari, 70125, Italy

## Abstract

The centromere/kinetochore interaction is responsible for the pairing and segregation of replicated chromosomes in eukaryotes. Centromere DNA is portrayed as scarcely conserved, repetitive in nature, quickly evolving and protein-binding competent. Among primates, the major class of centromeric DNA is the pancentromeric α*-*satellite, made of arrays of 171 bp monomers, repeated in a head-to-tail pattern. α-satellite sequences can either form tandem heterogeneous monomeric arrays or assemble in higher-order repeats (HORs). Gorilla centromere DNA has barely been characterized, and data are mainly based on hybridizations of human alphoid sequences. We isolated and finely characterized gorilla α*-*satellite sequences and revealed relevant structure and chromosomal distribution similarities with other great apes as well as gorilla-specific features, such as the uniquely octameric structure of the suprachromosomal family-2 (SF2). We demonstrated for the first time the orthologous localization of alphoid suprachromosomal families-1 and −2 (SF1 and SF2) between human and gorilla in contrast to chimpanzee centromeres. Finally, the discovery of a new 189 bp monomer type in gorilla centromeres unravels clues to the role of the centromere protein B, paving the way to solve the significance of the centromere DNA’s essential repetitive nature in association with its function and the peculiar evolution of the α*-*satellite sequence.

In multicellular eukaryotes the pairing and segregation of replicated chromosomes in mitosis and meiosis is essential to guarantee the complete and correct chromosomal complement in daughter cells. This process is mediated by the synchronized work of the spindle apparatus that connects to chromosomes through a proteinaceous bridge called a kinetochore^1^. The chromosomal counterpart of this molecular link is represented by the centromere. Throughout the mammalian orders, as well as more generally among all higher eukaryotes, the centromeric chromatin has been initially and consistently described by the incorporation of a histone H3 variant, the centromere protein A (CENP-A)^2^,^3^, and the recruiting of the centromere protein B (CENP-B)^4^.

Focusing on the primary sequence of the centromeric DNA, its low conservation as well as its rapid and distinctive evolution are copiously described. Nonetheless, it is generally acknowledged that there is an outstanding maintenance of features, such as its repetitive nature, structure and protein-binding competence^5^. The centromeric DNA is AT-rich and highly repetitive in almost every kind of plant and animal studied to date (with the exception of the holocentric centromeres of *C. elegans*)^6^,^7^. This constant co-occurrence between the centromeric functionality and repetitive DNA has been interpreted as evidence for a functional role of the satellite DNA, helping the assembly of the kinetochore and allowing a perfect timing at chromosome segregation.

Among primates, the major class of centromeric DNA is the pancentromeric α*-*satellite, composed of long stretches of 171 bp monomers, tandemly repeated in a head-to-tail pattern that extends for ~250 kbp up to ~5 Mbp per chromosome^8^,^9^,^10^. These sequences have been identified throughout the primate order, including great apes, Old World and New World monkeys^11^,^12^,^13^,^14^, with the exception of the suborder Strepsirhini^15^. α*-*satellite DNA is the most abundant repetitive DNA in all primate species studied, making up to 3–5% of each chromosome^10^,^16^.

The α-satellite can be variously classified, depending on its (i) primary sequence, (ii) multimeric structure and (iii) localization with respect to the centromere.

(i) Sequence analyses revealed that all existing primate types of alphoid monomers are most likely to be derived from only two ancient types of units and are therefore designed as “A” or “B” monomers^17^. (ii) α-satellite sequences can either form tandem heterogeneous monomeric arrays (10–40% divergence between individual monomers) or organize multimeric structures assembled in a period known as higher-order repeats (HORs)^18^,^19^,^20^,^21^,^22^,^23^. HOR units may be composed of two to over 30 monomers and are tandemly repeated several hundreds to thousands times per single centromere^13^,^24^,^25^. (iii) Where investigated, multimeric arrays have been found to contribute to the bulk of the centromeric chromatin, bordered by a monomeric α-satellite that acts as a junction to the pericentromeric regions^19^,^20^,^26^.

In HORs, monomers within a period differ greatly in sequence, while monomers standing at corresponding positions of different periods are virtually identical (<2% sequence divergence)^20^. The presence of alphoid HORs has been reported throughout the superfamily Hominoidea and is the evolutionary result of sequence homogenization created by molecular drive mechanisms, such as amplification, unequal crossing-over and gene conversion^27^,^28^. In particular, homogenization shows three patterns: local homogenization in tandem, intrachromosomal homogenization patterns that are regional but not in tandem, and interchromosomal or interarray patterns^29^.

Among the centromeric proteins that have been characterized, there are two main proteins directly binding the alphoid DNA: pJα and CENP-B. These proteins recognize the 17 bp pJα-motif (CTAPyGGTGPuAAAAGGAA) and CENP-B box (PyTTCGTTGGAAPuCGGGA) within A- and B-type monomers, respectively^17^. Modern great ape centromere organization emerged from ancestral A-type monomers mixed with more recent B-type monomers^17^. Like other tandem satellite families, the α-DNA evolves through molecular drive by mechanisms such as unequal crossing-over, gene conversion and transposition^16^,^30^,^31^. Originally, unequal crossover occurred between two similar monomers, creating tandem duplications (ancestral centromeric repeats of monomeres, ACRMs). Subsequent unequal crossovers were able to expand tandem arrays until a subset of ACRMs was multimerized into higher-order α-satellites thus creating several distinct HOR alphoid DNA families. HOR refers then to a structure in which multiple copies of the fundamental repeat units appear periodically^19^.

Due to the extremely high sequence identity of higher-order α-satellite monomers, unequal crossovers are much more likely to happen in HORs rather than in monomeric α-satellites causing different rates of evolution between them. Indeed, less conservation and higher intraspecies homogenization of orthologous HOR units than orthologous monomeric α-satellites have been described in closely related species^32^,^33^.

Based on monomer composition, structure and distribution, HOR DNA in great apes shapes different suprachromosomal families (SFs). In humans, for example, α-satellite sequences have been grouped into five SFs: SF1-5, each characterized by its own specific chromosomal distribution^17^. The five human SFs were initially revealed by restriction site periodicity and then identified by sequence-based phylogenetic analyse^19^,^34^,^35^. SF1, SF2 and SF5 are dimeric, i.e., contain two different monomers (A- and B-type) alternating regularly, as in SF1 and SF2, or are irregularly assembled, as in SF5^36^,^37^,^38^; SF3 is pentameric, i.e., formed by five different types of monomers (two A-type and three B-type)^13^; SF4 is monomeric, i.e., shaped by arrays of equally related monomers^35^. SF1-3 are organized in HORs and often designated as “new families” compared to the ancestral SF4 and SF5 and compose the centromeric region of all human chromosomes except the Y chromosome.

Conserved CENP-B boxes are located mostly in dimeric SFs and are regularly distributed every other monomer^39^,^40^. It has also been hypothesized that CENP-B dimers have a key role in the assembly of the centromeric chromatin by juxtaposing CENP-B boxes in α-satellite arrays^40^,^41^,^42^.

Since several studies have coupled the centromere function to higher-order rather than monomeric α-satellite in humans, the fairly recent creation of the “new families” may look intriguing and somehow counterintuitive^33^,^39^,^43^. Despite having been deeply characterized in humans, α-satellite structure and organization knowledge in primates has been primarily based on hybridizations of human alphoid sequences and restriction patterns. Among great apes, chimpanzee contains human-like HORs^44^, while orangutan mostly displays a basic monomeric organization, with HORs being very rare^45^. Nevertheless, the monomer primary sequence has been maintained similar enough to allow all human SFs to cross-hybridize with orangutan chromosomes at low stringency conditions.

Information on gorilla centromeric DNA is particularly meager and no details on its evolutionary history have been previously reported^46^. Here, we analyze the centromeric DNA in gorilla with several different approaches to achieve a broad display of organization and evolutionary history of the gorilla-specific α-satellite. Parallelisms with human alphoid sequences as well as gorilla-specific distinctive traits of the alphoid DNA were found.

## Results

Our investigation of gorilla centromeres was first achieved by isolating gorilla (GGO) centromeric DNA using human alphoid DNA sequence similarities^47^,^48^,^49^ and subsequently collecting GGO bacterial artificial chromosome (BAC) α-satellite clones and long gorilla centromeric sequences from online databases (whole-genome shotgun sequence, WGSS). Long sequences, such as BAC clones and WGSS, were essential to characterize the alphoid DNA structure and organization.

**Figure 1:**
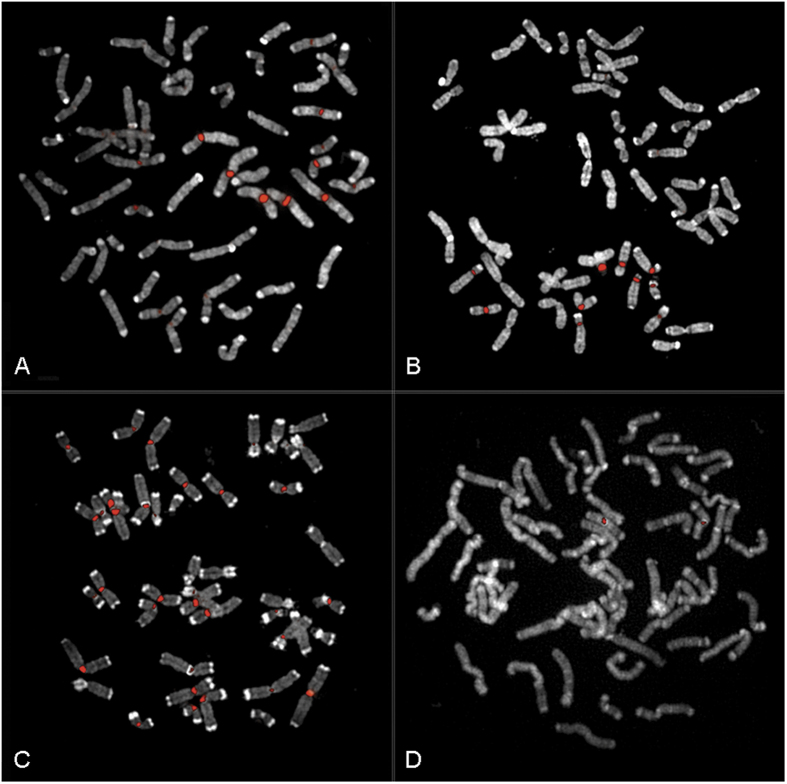
FISH experiments on GGO metaphases, using gorilla alphoid probes. (**A**) The plasmidic clone G.100 as an example of Group 1. (**B**) The plasmidic clone G.84 as an example of Group 2. (**C**) The plasmidic clone G.18 as an example of Group 3. (**D**) The plasmidic clone E.31 as an example of Group 4.

We isolated the GGO α-satellite by PCR amplification of gorilla genomic DNA using α-27/α-30 primers obtained from the most conserved regions of human alphoid consensus^50^,^51^. We then used the amplicons as probes in fluorescence *in situ* hybridization (FISH) experiments on gorilla metaphase chromosomes. The centromeric region of each chromosome was highlighted and no distribution or intensity differences were observed under either low or high stringency conditions. We cloned the amplicons and tested the clones by FISH on gorilla metaphase spreads; two main hybridization patterns were found (Groups 1 and 2, Table S1, Supplementary Note-Section 1), revealing two specific groups of centromeric sequences as main component of gorilla centromeres. A third subset of plasmids hybridized to chromosomes belonging to both Groups 1 and 2 (Group 3) and, lastly, there were pericentromeric clones hybridizing to only one pair of chromosomes (Group 4) (Table S1, Fig. 1). We also found “exceptional” clones that hybridized to all the chromosomes of one of the two main Groups (1 or 2) plus one single chromosome of the other group: Group 1 plus chromosome XVII or Group 2 plus chromosome V (Table S1 and Supplementary Note-Section 1).

To fully characterize the organization of centromeres in the gorilla genome, we collected gorilla α*-*satellites containing long-insert BAC clones (n = 41) and tested them by both FISH and *in vitro* enzymatic restriction. BAC FISH results on GGO metaphases were concordant to the previous results showing the same two main groups of patterns. Restriction patterns revealed that BACs from Group 1 were composed of dimeric sequences, while BACs belonging to Group 2 had a more complex and heterogeneous organization (Table S1, Supplementary Note-Section 2).

The same approach, from short-insert clones to long fully sequenced arrays, was used to perform α*-*satellite analysis by sequence. A subset of plasmids representative of the whole pool was sequenced, generating about 38 kbp of gorilla-specific alphoid DNA. We obtained 57 sequences ranging from 168 to 1536 bp in length (GenBank accession numbers JQ685164.1-JQ685171.1, JQ685175.1-JQ685179.1, JQ685186.1-JQ685223.1), containing up to eight α*-*satellite monomers per clone and 171 total monomers. Of the 57 sequences, 39 contained more than one monomer, thus being more informative to study the organization of alphoid arrays. Further, we retrieved and analyzed 66 WGSS containing α*-*satellites, 1293 to 26,052 bp in length, composed of 2351 monomers, making up 436 kbp of total sequence (Supplementary Note-Section 3).

We extracted 216 17-bp sequences composing one of the two centromeric protein-recognition domain (PRD) from plasmid inserts: 129/216 PRDs were pJα motif-positive, 89 possessed the CENP-B box, and 7 monomers had accumulated too many substitutions to be confidently grouped (Supplementary Note-Section 1). We annotated the “core” bases required for binding: 53/129 (41.1%) monomers containing the pJα-motif had the “core” pJα sequence required for protein binding completely conserved^17^, and 14/53 (26.4%) showed a perfect conservation of the entire 17 bp motif. Furthermore, 44/89 (49.4%) monomers containing the CENP-B box were positive for the binding “core”, i.e., contained all nine essential positions as described by Masumoto *et al.*^52^, and 32/44 (72.7%) showed a perfectly intact CENP-B box.

**Figure 2:**
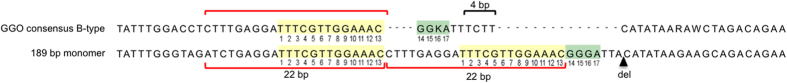
A schematic representation of the insertion in the 189 bp monomer obtained by aligning the longer units to the gorilla B-type consensus. Positions 15–77 are displayed for each consensus. Yellow and green bases represent the CENP-B box. del = deletion.

In our entire collection of gorilla alphoid monomers (2521 = 171 from plasmids + 2351 from WGSS), there were 104 extra-long monomeric units (~189 bp), all containing the same insertion. The insertion interrupted the CENP-B box at the 13^th^ position and duplicated 22 bp upstream (the unfinished CENP-B box plus nine more bases) creating a new complete and fully conserved CENP-B box. A 4 bp deletion downstream finally resulted in an 18 bp insertion (Fig. 2). All of these longer monomers, when derived from plasmid inserts, belonged to Group 2.

**Figure 3:**
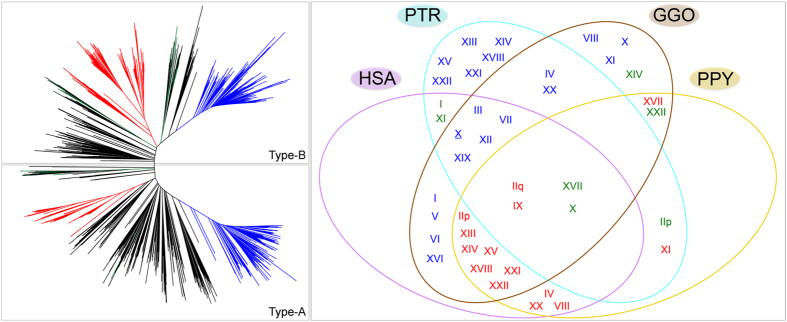
Left panel. Phylogenetic tree built by ClustalW showing all 2521 gorilla alphoid monomers extracted from WGSS plus our plasmid clones. **Right panel.** Venn diagram of the chromosomal hybridization pattern of the gorilla HOR alphoid suprachromosomal families in great apes according to data both in this work and in the literature. Colors and SF distributions are displayed as follows. Blue: SF1; Red: SF2; Green: SF3; Black: others.

We multi-aligned all the gorilla alphoid monomers (2521) and constructed a phylogenetic tree. A- and B-type monomers were clearly separated in the two main branches (Fig. 3). We derived a general gorilla α-satellite consensus sequence from the multi-alignment, and two gorilla-specific A- and B-type consensus sequences (Table 1).

As reported in human^17^, a CENP-B box can sporadically be found in A-type monomers and likewise the pJα motif in B-type monomers. Therefore, we calculated the p-distances between each GGO alphoid monomer extracted from plasmid inserts and the two GGO A- and B-type consensuses: 98.9% (92/93) of the A-type alphoid units contained the pJα motif, and 84.4% (65/77) of the B-type monomers contained the CENP-B box domain (see Methods section). In addition, we found no evidence of a perfect conservation of the essential position of CENP-B box in the type-A monomers nor the “core” of pJα motif perfectly conserved in the type-B monomers. As a sign of regularity and precise organization, 21/39 sequences containing more than one monomer showed a perfect alternance of CENP-B box and pJα motif monomers (Table S2, Supplementary Note-Section 3).

WGSS and plasmid sequences were examined for higher-order periodicities by BLAST comparisons, Tandem Repeats Finder (TRF) and dot plot. 31/66 WGSS and 5/46 plasmid sequences displayed higher order organizations (dimeric, tetrameric, pentameric or octameric) (Table S3, Supplementary Figure S1, Supplementary Note-Section 3); the rest of the sequences were made up of monomeric arrays.

The analysis of the phylogenetic tree pointed out that dimeric sequences were composed of monomers from only two clusters of the tree (blue) while the octameric sequences were from three branches (red) (Fig. 3). Sequences with tetrameric or pentameric patterns (green branches) did not form a clearly separated group on the phylogenetic tree, but rather they mixed with monomeric arrays (black branches) (Tables S3, S4, Fig. 3).

The three groups of sequences were accounted for as representative of gorilla alphoid SFs: SF1, SF2 and SF3 (blue, red and green branches in Fig. 3, respectively; Tables S3 to S5). Each group of sequences was individually studied and gorilla SF-specific substitutions were found (Supplementary Note-Section 3).

**Figure 4:**
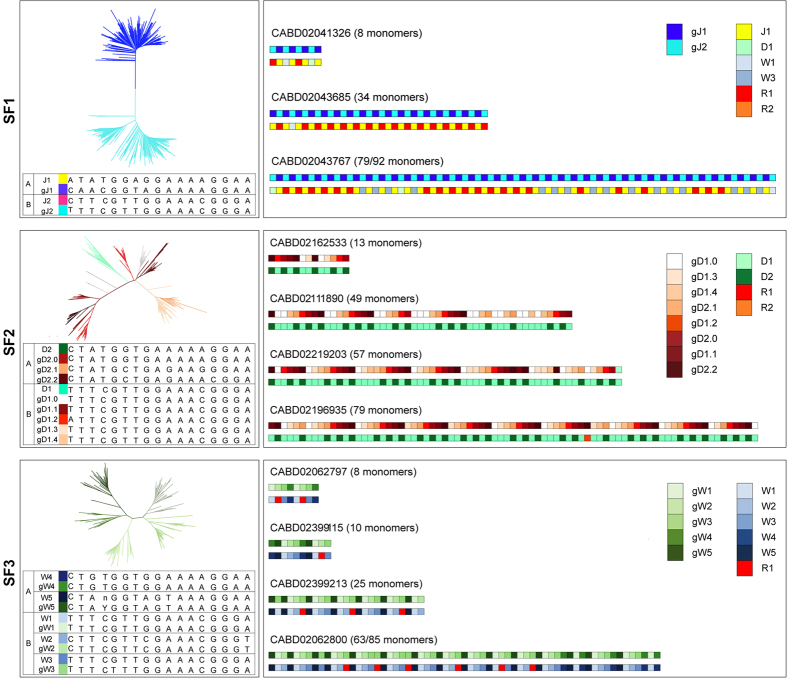
Examples, for each SF, of the succession of monomer types obtained by p-distance matrices analysis with both the 16 gorilla monomer units (panels on the right, first lane for each clone) and the 12 human consensus sequences (panels on the right, second lane for each clone) (see Methods section). Gorilla consensus names have been assigned based on p-distances to human consensus monomers (e.g. the five gorilla type-B consensuses belonging to SF2 are all named “D1” as the human type-B SF2 consensus, and further specified as .0 to .2 because of the corresponding growing divercence). SF-specific phylogenetic trees and substitutions of the PRD are also shown (panels on the left). Each position was considered unambiguous if more than 50% of monomers had the same nucleotide at that position. The ambiguous positions were designated as n. V is A/C/G; B is C/G/T; M is A/C; R is A/G; W is A/T; S is C/G; Y is C/T; K is G/T; F is -/A; I is -/C; J is -/G.

We can infer that SF1 (16 WGSS and 7 plasmid clones, Table S4) is dimeric and composed of two regularly alternating types of monomers (A-type and B-type); both presented conserved essential positions in the PRD (Table S3, Supplementary Note-Section 3). SF2 (10 WGSS and 11 plasmid clones, Table S4) is formed by eight different kinds of monomers: three A-type and five B-type monomers. All but two of the three A-type monomers showed a total conservation of the essential positions of the PRD. Moreover, two out of the eight monomers composing SF2 contained the 22 bp insertion previously described, and in four analyzed WGSS the insertion was further duplicated (CABD02196940.1, CABD02378856.1, CABD02378856.) or triplicated (CABD02196967.1) (Fig. 4, Table S3, Supplementary Note-Section 3). SF3 (5 WGSS, 1 plasmid, Table S5) is organized as a pentamer (three B-type and two A-type monomers); their PRDs are all conserved in the essential positions except for one of the two A-type monomers and one of the three B-type monomers (Table S3, Supplementary Note-Section 3). Sequences were assigned to SFs exclusively in the case of concordant results between the different analyses and when unequivocally composed of monomers distributed in one of the three groups of clusters of Fig. 4 (Table S4).

Furthermore, each monomer was more similar to its own family consensus than to the consensus sequences of all other monomeric types, thus supporting subfamily classification (Supplementary Note-Section 3). The remaining monomers from 35 WGSS and 27 plasmid sequences not belonging to SF1-3 often intermingled with the SF3 units in the phylogenetic tree (black branches, Fig. 3) or, rather, composed the more ancestral cluster of sequences at the boundary between A- and B-type units. Phylogenetic trees using these sequences showed no clear clusterization of monomers (Supplementary Figure S2) and all were arranged in monomers by TRF and dot plot (Table S3). Indeed, although we can roughly distinguish A- and B-type monomers, they did not form any other HOR family as they showed a totally irregular organization (Fig. 3, Supplementary Figure S1).

We generated 16 new gorilla alphoid consensus sequences, specific for the three different SFs (two monomers for SF1; eight monomers for SF2; five monomers for SF3) and one for the monomeric arrays (gM1) (Table 1). By performing multiple sequence alignment and building p-distance matrices between the 2521 gorilla alphoid monomers and the 12 human alphoid consensus sequences, we revealed that gorilla SF1, SF2, and SF3 parallel human SF1, SF2, and SF3 (Table 2, Supplementary Note-Section 3) (see Methods section). Gorilla A-type monomers gJ1 (SF1) always correspond to human monomer type J1, while the gorilla B-type monomers of the same family have significantly diverged from the human counterpart (J2) (Fig. 4, Supplementary Note-Section 3). Conversely, the SF2 monomer composition revealed an extensive conservation of the B-type monomers. Gorilla SF3 is the less homogeneous SF; the five monomers very rarely form long pentameric stretches (tetramers are more common) (Supplementary Note-Section 3M).

With the correspondence between gorilla and human alphoid SFs ascertained, we gained more informative insight into the evolution of these sequences by hybridizing all the gorilla BAC clones previously described plus plasmids representative of all subgroups (1, 2, 3 and 4) on human (HSA) metaphases (79); then, an exemplificative subset (16) was hybridized on chimpanzee (PTR) and orangutan (PPY). In HSA, all centromeres hybridized with gorilla SF1 and SF2 probes were homologous to the gorilla SF1 and SF2 chromosomes, respectively. However, human chromosomes 4, 8 and 20 did not show this concordancy; the centromeres of these three chromosomes harboring human SF2 sequences showed signals using gorilla SF2 clones while in gorilla they hybridized with clones containing SF1 arrays, thus showing a different organization of these centromeres in human and gorilla. Moreover, due to the evolutionary translocation, gorilla centromeres V and XVII contain human centromeres 17 and 5^53^,^54^. Despite the translocation, we observed signals on chromosome V using gorilla SF1 and chromosome XVII using SF2 clones in both gorilla and human. The only plasmidic clone containing SF3 sequences showed signals on chromosomes 17 and 11 (Tables 3 and S6). Furthermore, the “exceptional” clones hybridized randomly to human centromeres without maintaining the SF specificity (and hence showing a hybridization pattern similar to the gorilla clones from Group 3). Lastly, centromeric signals on human chromosomes 6 and 10 were not detected with any of the probes used (Fig. 3).

Gorilla SF1 sequences mostly mapped on PTR non-orthologously, while they did not hybridize at all on PPY chromosomes in either high or low stringency conditions. Gorilla SF2 clones, instead, highlighted all PPY chromosomes; although on PTR chromosomes very few signals were detected, mainly on chromosomes 9, 11 and 13 (XI, IX and IIq, respectively). The gorilla SF3 clone hybridized on IIp, XVII and X in PTR and (more faintly) in PPY (and on PTR I, VI and XI and PPY XXII) (Tables 3 and S7, Fig. 3).

## Discussion

The centromeric α-satellite DNA in primates is typically composed of tandem repeats of a highly divergent 171 bp monomer repeat unit, with pairwise sequence identities of 60–80% within and between chromosomal subsets^20^.

At present, the only data available about the α-satellite in gorilla come from comparative hybridization experiments, in which traditionally human alphoid probes have been used. The most detailed study in this species has uniquely shown the extensive conservation of the X chromosome satellites^46^.

In this work we have explored the centromere DNA in gorilla, investigating more than 8 Mbp of α-satellite, representing roughly the 9% of the total centromeric DNA sequence available for this species.

Our data indicate that the gorilla α-satellite is organized in at least three different HOR subfamilies. The SF1 and SF3 mantain the human organization, while SF2 is represented by an unusual octameric HOR which is totally absent in humans and is likely present on several chromosomes in gorilla (Fig. 3). Gorilla HOR SFs, like in human^19^,^20^, show very high sequence similarities between adjacent multimeric units—up to 94% in SF1 and SF3 and up to 99% in SF2 HORs—thus displaying SF2 arrays in gorilla as more incisively homogenized.

FISH results revealed differences in extension and relative localization of HOR versus monomeric arrays. Gorilla SF1, SF2 and SF3 probes gave more intense and centromerically located signals than probes composed of monomeric arrays (Supplementary Figure S3). These data demonstrated that in gorilla, as in human, SF4 and SF5 arrays, are less abundant and localize at the borders of centromeres opposite to SF1-3 that instead create the bulk of centromeres^19^,^20^,^26^. Moreover, our data showed that gorilla centromeric sequence organization, as in human, is quite complex containing more than one type of array^48^.

*In vitro* studies proved homodimer CENP-B binds by each amino terminus to a CENP-B box sequence^55^,^56^. This creates a complex that contains two CENP-B polypeptides and two DNA molecules^57^, suggesting that the role of CENP-B *in vivo* might be able to assemble the higher-order structure of centromere satellite DNA arrays by juxtaposing pairs of CENP-B box sequences. Subsequently, CENP-B might have a role in the establishment of heterochromatin, thereby facilitating cohesion of sister chromatids around the centromere. Although the centromeric DNA is not conserved among species, the CENP-B–CENP-B box interaction is significantly conserved among mammals and may play an important role in the establishment of the specific structure of the kinetochore^40^. We have analyzed the long-range distribution of the protein-binding sites through α*-*satellite DNA in gorilla chromosomes and found both very high conservation of the CENP-B box essential positions and regular alternance between monomers. In particular, CENP-B- and pJα-binding monomers alternated perfectly in SF1, while showing B-B^(*)^-B*-A-B-A-B-A and B-B-B-A-A in SF2 and SF3, respectively (Fig. 4, for details).

Moreover, the frequency of conserved CENP-B boxes or pJα motifs was much lower in monomeric arrays than in HORs, as previously demonstrated in human^21^. Subsequently, diagnostic mutations specific to each of the SFs were detected, revealing a very high degree of homogenization (Supplementary Note-Section 3).

Hybridization patterns of gorilla centromeric DNA, together with previously published data and sequence analyses, let us conclude that SF1 sequences emerged in the GGO-PTR-HSA common ancestor. Indeed, there is no presence in orangutan^47^. Their localization in GGO, HSA and PTR indicates that they were created on chromosomes III, VII, X, XII and XIX and were subjected to extensive relocalizations in PTR (Fig. 3).

Clones made of gorilla SF2 sequences hybridized to all orangutan chromosomes, except for chromosomes VI and XII, all acrocentric chromosomes in gorilla and human, and very few chimpanzee centromeres on chromosomes IIq, IX, and XI. Human SF2 sequences, used as probes, were previously found on chromosomes IIq^58^ and IX^48^ in chimpanzee and on chromosome XVII in gorilla^59^, while pancentromeric hybridization had been concordantly observed in orangutan^47^. Gorilla SF3 sequences scarcely hybridized to orangutan metaphases, and signals were detected in chimpanzee on chromosomes XI, XVII and X, in addition to few others, consistent with prior reports^48^.

The positive hybridizations obtained in PPY with gorilla SF2 and SF3 probes, might suggest the presence of these specific sequences in this species; nevertheless, since the high similarity of SF2 and especially SF3 arrays to the more ancient SF4 and SF5 monomers, our results might rather be produced by cross-hybridizitions with SF4 and SF5. Hence, before tracing evolutionary hypothesis involving SF2 and SF3 sequences, more focused studies are needed.

The conservation of both the sequence and location of SF2 between human and gorilla chromosomes was previously published^47^; however, our data prove for the first time that SF1 sequences are also present on homologous chromosomes in these two species, thus giving evidence of a higher similarity of alphoid DNA between human and gorilla than between human and chimpanzee. The only exceptions to the conservation of centromere-specificity between human and gorilla are the centromeres of the chromosomes IV, VIII and XX; all of these chromosomes underwent pericentric inversions during their evolutive history in great apes, and this could most probably have affected the centromere evolution^60^,^61^,^62^,^63^.

Furthermore, human hybridization patterns show the exclusivity of either SF1 or SF2 sequences on human chromosomes, while in gorilla there are exceptions to this rule in regards to chromosomes V and XVII, which we found to contain sequences from both SF1 and SF2. An exchange of centromeric sequences might have happened following the translocation event that created the human-gorilla V and XVII chromosomes^53^,^54^.

We described two out of the eight gorilla SF2 monomer types containing a highly conserved insertion (sometimes present two or three times). This extra 22 bp sequence breaks the CENP-B box, duplicates 22 bp upstream, and creates a new complete CENP-B box. These “scars” from the creation of “altered monomers” are similar to the mechanism proposed by Alexandrov *et al.* to explain the evolution of the S4-S5-S3 alphoid trimers in *Chiropotes* and *Pithecia*: they arose from an unequal crossover between two different monomers of an S3-S4 dimer that were shifted by 25 bp. As a result, S5 monomers are chimeric and contain a 25 bp duplication^19^. Both of these events gain interest in light of the assessed very high similarity between the CENP-B and the pogo and Tigger proteins coded by the *pogo* superfamily of transposable elements^64^. The human CENP-B and the transposases of the pogo type have highly similar primary sequences, a domain responsible for the coordination of the DNA cleavage during transposition and a DNA binding domain^65^.

The mechanism of transposition of these elements requires the approach of a pair of terminal inverted repeats (TIRs) to a protein-DNA complex and a following endonucleolytic cleavage and strand transfer. The elements *Tigger1* and *Tigger2* have an open reading frame similar to CENP-B, and the TIRs motifs for *Tigger2* are highly similar to the CENP-B box. It is then suggested that CENP-B derived from a transposase. This allowed us to speculate that the functional role of CENP-B at the primary constriction could not strictly be related to the centromere function or kinetochore assembly but rather to the modulation of evolution of the alphoid DNA by inducing recombination hotspots in the case where CENP-B had retained some transposase-associated activities. If this is the case, CENP-B would create nicks 10–20 bp upstream of the CENP-B box after having aligned two of them due to the dimerization of two CENP-Bs.

The role of a CENP-B-mediated transposition in the evolution of the centromere satellite could also possibly complement gene conversion and unequal exchange in solving the conundrum caused by evidence for both recombination and crossover suppression at centromeres^31^. Indeed, recombination events in α*-*satellites happen with a higher frequency 10–20 bp upstream of juxtaposed CENP-B boxes, which is actually possible when the CENP-B dimerizes^64^,^66^,^67^,^68^.

Our data provide new details into the organization and evolution of the centromere DNA in primates, giving direct evidence for the existence of higher-order alphoid SFs in gorilla centromere sequences. We show conserved distribution of SF1 and SF2 between human and gorilla and highlight an astonishing change specific to the gorilla lineage in the organization of SF2 sequences while maintaining a very high degree of sequence conservation. Indeed, SF2 in gorilla is uniquely octameric and periodically includes highly conserved 189 bp monomers. The discovery of these monomers allowed us to propose a role of CENP-B in centromere activity, closely related to the evolution of these sequences. Our model links the complexity of the centromeric sequence to its function and it traces future directions to study centromere functional properties.

## Methods

### Cell lines

Metaphase spreads and interphase nuclei were prepared from lymphoblastoid or fibroblast cell lines of *Pan troglodytes* (PTR) PTR5 (Bianka, Budapest zoo), *Gorilla gorilla* (GGO) (GGO5 ECACC CB1620) and *Pongo pygmaeus* (PPY) (Sinjo, Hamburg zoo). Human (HSA) metaphase spreads were prepared from Phytohemagglutinin-stimulated peripheral lymphocytes of normal donors by standard procedures. All metaphase spreads were obtained from female individuals.

### FISH and Image Analysis

FISH experiments were performed using BAC clones and plasmids directly labeled by nick-translation with Cy3-dUTP as previously described^69^ with minor modifications. Hybridization was performed at 37 °C in 2x sodium chloride sodium citrate (SSC), 50% (v/v) formamide, 10% (w/v) dextran sulfate, 3 μg C0t-1 DNA, and 3 mg sonicated salmon sperm DNA, in a volume of 10 μl. Post hybridization washing was at high stringency (60 °C in 0.1X SSC, three times) or at low stringency (37 °C in 2X SSC, 50% formamide, three times, and 42 °C in 2X SSC, three times). Nuclei and chromosome metaphases were DAPI-stained. Digital images were obtained using a Leica epifluorescence microscope equipped with a cooled CCD camera. Fluorescence signals detected with Cy3 filters and chromosomes and nuclei images detected with DAPI filter were recorded separately as grayscale images. Pseudocoloring and merging of images were performed using Adobe Photoshop software.

### Polymerase Chain Reaction (PCR) Labeling

DNA probes were directly labeled with Cy3-dUTP by PCR labeling; 200 ng of labeled probe was used for the FISH experiments. The use of PCR labeling avoids the possible contamination from genomic DNA by nick translation labeling of PCR products. PCR labeling was carried out in a final volume of 20 μl that contained 100 ng PCR product, 2.5 μl reaction buffer 10X, 2 μl MgCl_2_ 50 mM, 0.5 μl each primer 10 μM, 0.5 μl dACG 2 mM, 2.5 Cy5-dUTP 1 mM, 5 μl BSA 2%, and 0.3 μl Taq polymerase 5 U/μl.

### Library screening

Library-hybridization was carried out according to the protocol available at CHORI BACPAC resources (http://bacpac.chori.org/highdensity.htm). The CH255 segment 1 represents a ~7.0-fold clone coverage library (http://bacpac.chori.org).

### Alpha PCR

Gorilla genomic DNA were obtained from gorilla lymphoblastoid cell lines by standard methods^70^. α27 (CATCACAAAGAAGTTTCTGAGAATGCTTC) and α30 (TGCATTCAACTCACAGAGTTGAACCTTCC) primers were used to amplify genomic DNA by Polymerase Chain Reactions. They were obtained from the most conserved regions of human alphoid consensus^50^,^51^.

The PCR was performed as previously described^14^: 2 min initial denaturation at 94 °C, followed by 10 cycles of: 95 °C for 15s, 60 °C for 30s, and 72 °C for 1 min; followed by 20 cycles of 94 °C for 15s, 58 °C for 30s, and 72 °C for 1 min (20s more each cycle). Final extension was at 72 °C for 7 min (and then at hold 12 °C).

The reaction mixture consisted of 5 μl dNTPs (10X), 0.5 μl each primer (10 μM), 0.3 μl Platinum Taq DNA polymerase (5 U/μl), 1.5 μl MgCl_2_ (50 mM), 5 μl reaction buffer (Invitrogen) (10X), 3 μl of DNA template (50 ng/μl), and water up to 25 μl.

PCR products were analyzed by 1% agarose gel electrophoresis. They were labeled and used as a probe for FISH experiments on GGO metaphase spreads.

### Cloning

PCR products were cloned in pCR-XL-TOPO using the standard protocol Topo cloning XL PCR kit (Invitrogen).

### Southern Blot Analysis

Genomic DNAs from gorilla lymphoblastoid cell lines were prepared by standard procedures^70^. Endonuclease digestions were performed using a 4-fold excess of enzyme under the conditions suggested by the suppliers. Gel electrophoresis was performed in 1X tris-acetate (1X TAE 540 mM Tris-acetate, 1mMethylenediaminetetraacetic acid, EDTA). Genomic DNAs were run in a 0.8% agarose gel for 16–18 h, denatured, and DNA transferred to Hybond membrane (Amersham), using as transfer buffer NaOH/NaCl (sodium chloride NaOH 0.25 M, sodium cloride NaCl 1.5 mM).

Clone inserts (50 ng) were labeled with 32P-dATP (3,000 Ci/mmol; Amersham) by using random oligomer priming. Filters were exposed and developed using storm imaging system.

### Sequence and phylogenetic analyses

FASTA-formatted sequences were obtained corresponding to each gorilla α-satellite monomer and each sequence was analyzed by NCBI Blast2Sequences tool (http://blast.ncbi.nlm.nih.gov/bl2seq/wblast2.cgi) (BlastN program), aligning each first monomer with the entire sequence using default parameters (1 as reward for a match, −2 as penalty for a mismatch, and 5 and 2 as open and extension gap penalties). Gorilla α-satellite sequences (WGSS) were searched by using plasmid sequences as queries in a BLAST search on *Gorilla gorilla* (taxid: 9593) NCBI databases. High amount of sequences was available thank to the recent gorilla genome sequencing project^71^. Multiple sequence alignments and consensus sequence extractions were performed using Clustal W^72^. Phylogenetic analyses were conducted in MEGA6 (Molecular Evolutionary Genetic Analysis, version 6.06)^73^. The evolutionary history was inferred using the Neighbor-Joining method^74^. The optimal tree with the sum of branch length = 77.65354306 is shown. The tree is drawn to scale, with branch lengths in the same units as those of the evolutionary distances used to infer the phylogenetic tree. A bootstrap test with 500 replicates and pairwise deletion parameters was conducted to evaluate the statistical significance of each node (Supplementary Figure S4). The evolutionary distances were computed using the p-distance method and are in the units of the number of base differences per site. The analysis involved 2521 nucleotide sequences. All ambiguous positions were removed for each sequence pair. There were a total of 299, 193, 259 and 174, positions in the final dataset for the entire collection of monomers, for SF1, SF2 and SF3 sequences, respectively.

## Additional Information

**Accession codes:** GenBank accession numbers JQ685164.1-JQ685171.1, JQ685175.1-JQ685179.1, JQ685186.1-JQ685223.1.

**How to cite this article**: Catacchio, C. R. *et al.* Organization and evolution of Gorilla centromeric DNA from old strategies to new approaches. *Sci. Rep.* **5**, 14189; doi: 10.1038/srep14189 (2015).

## Supplementary Material

Supplementary Information

Supplementary Dataset

## Figures and Tables

**Table 1:** Gorilla alphoid consensus sequences and their size.

sequence ID	sequence	size (bp)
GGO_consensus	AATCTGCAAGTGGATATTTGGASYSYTTTGAGGVCTTCGKTGGAAAMGGRAATWTCT TCATATAAAAACTAGACAGAAGCATTCTCAGAAACTTCTTTGTGATGTGTGCATTCAACT CACAGAGTTGAACCTTYCTTTTGATAGAGCAGTTTTGAAACACYYCTTTTTGTAG	172
GGO_consensus_Atype	AATTTGCAAGTGGABATTTCGAGCGCTTTGJGGCCTATGGTAGAAAFAGGAAATATCT TCATATAAAAACTAGACAGAAGCATTCTCAGAAACTWCTTTGTGATGTGTGIRTTCAACT CACAGAJKTGAACCTTTCTTTTGATAGAGCAGTTTTGAAACACTCTTTTTGTAG	172
GGO_consensus_Btype	AATCTGCAAGTGGATATTTGGACCTCTTTGAGGATTTCGTTGGAAACGGKATTTCTTCAT ATAARAWCTAGACAGAAGAATTCTCAGWAACTTCTTTGKGATGTWTGCBTTCAACTCA CAGAGTTGAACMTTCCTTTTGATAGAGCAGRTTTGAAACACTCTTTTTGTGG	170
gJ1	AATTTGCAAGTGGACATTTCAAGCGCTTTGGGGCCAACGGTAGAAAAGGAAATATCT TCGTATAAAAACTAGAGAGAATCATTCTCAGAAACCACTTTGTGATGTGTGCGTTCCACT CACAGAGTTTAACCTTTCTTTTCATAGAGCAGTTTGGAAACACTCTGTTTGTAA	171
gJ2	AGTCTGCAAGTGGATATTTGGACCTCTTTGAGGATTTCGTTGGAAACGGGATTTCT TCATCTAATGCTAGACAGAAGAATTCTCAGTAACTTCTTTGGGTTGCGTGTGTTCAACT CACAGAGTTGAACCTTCCTTTAGACAGAGCAGATTTGAAACCCTCTTTTTGTGG	169
gD1.0	AATCTGCAAGTGGATATTTGGATAGGTTTGAAGATTTCGTTGGAAACGGGAATATCT TCATATAAAATCTAGACAGAAGCATTCTCAGAAACTTCTTTGTGATATCTGCATTCAAGA CACAGAGTTGAATATTCCCCTTCATAGAGCAAGTTTGAAACACTCTTTTTGTGG	171
gD1.1	AATCTGCAAGTGGATATTTGGATAGCTTTGAAGATTTCGTTGGAAACGGGAATTTCT TCATATCAAATCGAGACAGTAGCATTCTCAGAAACTTCCTTGTGATATCTGCATTCAAGT CAGAGAGTTGAACATTCCCTTTCATAGAGCAGGTTTGAAACACTCTTTCGGTGG	171
gD1.2	AATCTGCAACTGGATATTTGGATAGATTTGAAGAATTCGTTGGAAACGGGAATATCT TCCAATAAAATCTAGACAGAAGCATTCTCAGAAACTTCTTTGTGATGCTTGCATTCAACT CATAGAGTTGAACATTCCCTATCATAGAGCAGGTTGGAAACACTCATTTTGTAG	171
gD1.3	AATCTGCAAGTGGATATTTGGATAGATTTGAGGATTTCCGTTGGAAACGGGATTACAT ATAAAAAGCAGACGGCAGCATTCTCCGAAATTTCTTTGCGATGTTTGCATTCAAGTCA CAGAGTTGAACATTCCCTTTCATAGAGCAGGTTTGAAACACTCTTTTTGTGG	168
gD1.4	AATCTGCAAGTGGATATTTGGGTAGATCTGAGGATTTCGTTGGAAACCTTTGAGGAT TTCGTTGGAAACGGGATTACATATAAGAAGCAGACAGAAGCATTCTCCGAAATTTCT TTGTGATGTTTGCATTCAAGTCGCAGAGTTGAACATTCCCTTTCATAGAGCAGGTTT GAAACACTCTTTCTGTAC	189
gD2.0	AATGTGGAAGTGGACATTTGGAGCGCTTTGAGGCCTATGGTGAAAAAGGAAATATCT TCCCATAAAACCTAGACAGAAGCATTGTCAGAAACTTCTTTGTGATGTGTGTACT CAACTAACAGAGTTGAACCTTCCTTTTGACAGAGCAGTTTTGAAACACTCTTTTTGTAG	171
gD2.1	TATCTAGAGGAGGACATTTCGAGCGCTTTCTGGCCTATGCTGAGAAGGGAAATATCT TCAAATAAAAACTAGACAGAAGCATTCTCAGAAAGTTGTTTGTGATGTGTGTCCT CAACTAACAGAGTTGAACCTTTGTTTTGATACAGCAGTGTGGAAACACTCTTTTTGTAG	171
gD2.2	TATCTGCAAGTGGGCATTTCGAGCGCTTTCAGGCCTATGCTGAGAAACGGAATATCT TCAAATAAAAACCAGACCGAAGCATTCTCAGAAACTTATTTGTGATGTGTGTCCT CACCTAACAGAGTTGAACGTTTGTTTTGATACAGCAGTTTGGAAACACTCTTTTTGTAG	171
gW1	AATCTGTAAGTGGATATTTGGACCCCTCTGAGGATTTCGTTGGAAACGGGATAAACT TCCCATAACTAAACGGAAGCATTCTCAGAAACTTCTTTGTGATGTTTGCATTCAGCTCA CAGAGTTGAACCTTCCTTTGATAGTTCAGGTTTGAAACACTCTTTTTGTAG	167
gW2	AATCTGCAAGTGCATATTTGGACCACCGAGTGGCCTTCGTTCGAAACGGGTATATCT TCACGTAAAAGCTAGGCAGAAGCATTCTCGGGAACTTCTCTGTGATGATTGCATTCAACT CACAGAGTTGGACACTCCTTTTGATAGAGCAGTTTTGAAACTCTCTTTTGGTAG	171
gW3	AATCTGCAAGTGGATATGTGGACCTCTTTGAAGATTTCTTTGGAAACGGGAATATCTTCA CATAAAAACTAAACAGAAGCATTCTCAGAAACTACTTTGTGATGATTGCATTCAACTCA CAGAGTTGAACATTCCTATTGATAGAGCAGTTTGGAAACACTCTTTTGGTAG	171
gW4	AATCTGCAAGTGGACATTTGGAGCGCTTTGAGGCCTGTGGTGGAAAAGGAAATATCT TCACATAAAAACTAGATAGAAGCATTCTCAGAAACTCTTTGTGATGATTGCATTCAACT CACAGAGTTGAACATTCCTTTTGATAGAGCAGTTTGGAAACACTCTTTTTGTG	169
gW5	AATCTGCAAGTGGAGATTTGGACTGCTTTGAGGCCTAYGGTAGTAAAGGAAATAACT TCATATAAAAACCAAACAGAAGCATTCTCAGAAAATTCTTTGTGATGATTGAGTTGAACT CACAGAGCTGAACATTGCTTTTGATGGAGCAGTTTCCAAACACACTTTTTGTAG	171
gM1	AATCTGCAAGTGGATATTTGGAGCGCTTTGAGGCCTATGGTGGAAAAGGAAATATCT TCACATAAAAACTAGACAGAAGCATTCTGAGAAACTTCTTTGTGATGTGTGCATTCATCT CACAGAGTTGAACCTTTCTTTTGATTGAGCAGTTTTGAAACACTCTTTTTGTAG	171

**Table 2:** Summary of the comparison between gorilla and human alphoid suprachromosomal families.

SF	species	units	monomer name	monomer type
SF1	gorilla	2	gJ1-gJ2	A-B
	human	2	J1-J2	A-B
SF2	gorilla	8	gD1.0-gD1.3-gD1.4-gD2.1-gD1.2-gD2.0-gD1.1-gD2.2	B-B^(*)^-B^*^-A-B-A-B-A
	human	2	D1-D2	B-A
SF3	gorilla	5	gW1-gW2-gW3-gW4-gW5	B-B-B-A-A
	human	5	W1-W2-W3-W4-W5	B-B-B-A-A

Note. Asterisks indicate the presence of extra long monomers; brackets mean that a scarce portion (32/100) of monomers are extra long units.

**Table 3:** Hybridization results of five illustrative gorilla centromeric clones on great ape metaphase chromosomes, classified by suprachromosomal family.

		**HSA chromosome**																									
**1**	**2**	**3**	**4**	**5**	**6**	**7**	**8**	**9**	**10**	**11**	**12**	**13**	**14**	**15**	**16**	**17**	**18**	**19**	**20**	**21**	**22**	**X**				
SF1	G.105	+++		+++		++		+++					++				++			+++					
	CH255-52M24	+++		+++		+++		+++			+++		++				+++			+++					
SF2	G.84				+++					+++				++	++	++			++		+++	+++	++		
	CH255-50P4		++		++				++	+++				++	++	++			+++		++	+++	++		
SF3	E.27											+++						+++							
		**PTR chromosome**																							
	**CLONE**	**1I**	**2III**	**3IV**	**4V**	**5VI**	**6VII**	**7VIII**	**8X**	**9XI**	**10XII**	**11IX**	**12IIp**	**13IIq**	**14XIII**	**15XIV**	**16XV**	**17XVIII**	**18XVI**	**19XVII**	**20XIX**	**21XX**	**22XXI**	**23XXII**	**X**
SF1	G.105		++				++									+++		++			++	++	++	+++	
	CH255-52M24		++	++					+++				++			+++	++		++		+++	+++		+++	
SF2	G.84									++		++		++											
	CH255-50P4											+++		+++											
SF3	E.27	++				++				+++			++							++					+++
		**GGO chromosome**																							
	**CLONE**	**1I**	**2III**	**3IV**	**4V**^a^	**5VI**	**6VII**	**7VIII**	**8X**	**9XI**	**10XII**	**11IIq**	**12IIp**	**13IX**	**14XIII**	**15XV**	**16XVIII**	**17XVI**	**18XIV**	**19XVII**^a^	**20XIX**	**21XX**	**22XXI**	**23XXII**	**X**
SF1	G.105	++		+++		++		+++	++	++												+++			++
	CH255-52M24	++		+++	++			++										+++							
SF2	G.84													+++					++	++			+++	++	
	CH255-50P4												++	++					+++	++			+++	+++	
SF3	E.27																		++	+++				++	++
		**PPY chromosome**																							
	**CLONE**	**1I**	**2III**	**3IV**	**4V**	**5VI**	**6VIII**	**7X**	**8XI**	**9XII**	**10VII**	**11IIq**	**12IIp**	**13IX**	**14XIII**	**15XIV**	**16XV**	**17XVIII**	**18XVI**	**19XVII**	**20XIX**	**21XX**	**22XXI**	**23XXII**	**X**
SF1	G.105	**NO SIGNAL**																							
	CH255-52M24	**NO SIGNAL**																							
SF2	G.84		+++	++	++		++	++	++		++	++	++	++	++	++	++	++	++	++	++	++	++	++	++
	CH255-50P4		+++	++			++	++				++					++	++	+++	++	++	+++	++	+++	
SF3	E.27												++							++				++	++

Note. Plus represents the intensity of the detected signals: “++” medium and “+++” strong.

^a^Gorilla chromosomes V and XVII contain the centromeres of human chromosomes 17 and 5, respectively.
