# Supplementary material for: Organization and evolution of Gorilla centromeric DNA from old strategies to new approaches
Source: Sci Rep. 2015 Sep 21;5:14189. doi: 10.1038/srep14189 (PMC4585704; doi:10.1038/srep14189)

Supplementary Information

Organization and evolution of Gorilla centromeric DNA from old strategies to new approaches

Catacchio CR[1], Ragone R[1], Chiatante G[1] and Ventura M[1†]

[1]Department of Biology, University of Bari Aldo Moro, Via Orabona 4, 70125 Bari, Italy

CONTENTS

Supplementary Figures

Supplementary Tables

Supplementary Note

Supplementary Figure Legends

Supplementary Figure S1. Upper panel. BLAST analysis of 39 gorilla centromeric sequences from plasmids: each first alphoid monomer was compared to the rest of the clone. **Lower panel.** Example of JDotter outputs (obtained aligning whole sequences against themselves). Monomeric, dimeric (SF1), octameric (SF2) and tetrameric (SF3) periodicities are shown.

Supplementary Figure S2. Phylogenetic tree of gorilla alphoid monomers (n = 777) extracted from arrays not belonging to SF1-3.

Supplementary Figure S3 FISH experiments on GGO metaphases, using gorilla alphoid probes showing differences in hybridization pattern and intensity. SF1-3 probes gave more intense and centromere-specific signals. **(A)** Plasmid G.100 (monomers from SF1); **(B)** Plasmid A1.50 (monomers from SF2); **(C)** Plasmid E.27 (monomers from SF3); **(D)** Plasmid A1.49 (monomers from "SF2exceptional").

Supplementary Figure S4 Phylogenetic trees of all gorilla alphoid monomers collected, of monomers belonging to SF1, SF2 and SF3. Bootstrap values higher than 75% are shown to assess support for nodes.

Supplementary Figures

Clones	size (bp)	units	%identity between repeats	Clones	size (bp)	units	%identity between repeats
**A1.12**	1211	6	80-80-77-77-82	**E.27**	677	3	80-75
**A1.19F**	688	3	81-75	**E.31**	682	3	83-83
**A1.19R**	755	3	79-85-78	**E.32**	680	3	86-87
**A1.36F**	859	4	84-83-81	**E.35**	509	2	76
**A1.36R**	727	4	81-77	**E.54**	510	2	82
**A1.40**	1536	8	79-81-84-80-80-79-76	**E.56**	509	2	87
**A1.44F**	933	5	82-78-82-83	**E.73**	1196	6	68-**95**-65-**93**-68
**A1.44R**	843	4	88-82-83	**E.83**	1022	5	82-80-80-74
**A1.49**	1017	6	85-86-85-89-90	**E.95**	683	3	88-89
**A1.50**	1043	6	80-83-82-79-97	**E.104**	683	3	88-89
**A1.52F**	965	5	84-83-80-77	**F.8**	1018	5	79-**87**-79-**91**
**A1.52R**	813	5	81-80-76-84	**F.18F**	919	5	79-73-79-77
**A1.60**	1207	6	80-81-78-78-83	**F.18R**	888	4	94-81-83
**A1.64**	1041	6	79-82-80-77-91	**F.94**	855	4	74-84-80
**A1.73**	682	3	87-81	**G.18**	1022	5	78-87-78-78
**E.1**	512	2	81	**G.84**	856	4	89-76-84
**E.5**	678	3	70-**92**	**G.97**	1189	6	80-81-77-74-79
**E.7**	511	2	75	**G.100**	1189	6	76-**92**-74-**88**-83
**E.12**	681	3	79-85	**G.105**	1185	6	68-**95**-65-**93**-68
**E.24**	511	2	76				

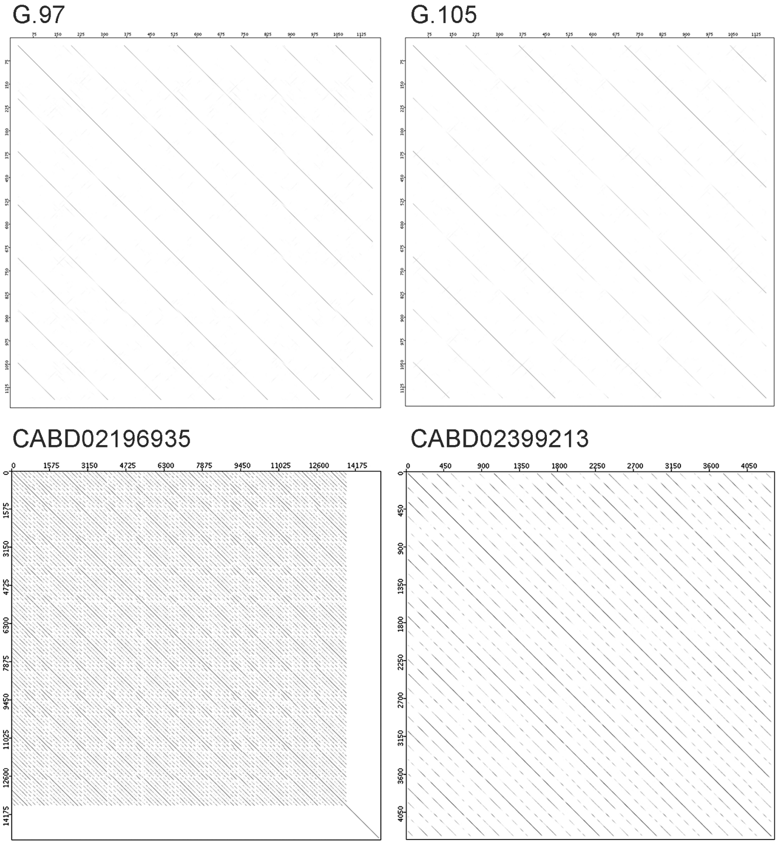

SupplementaryFigure S1

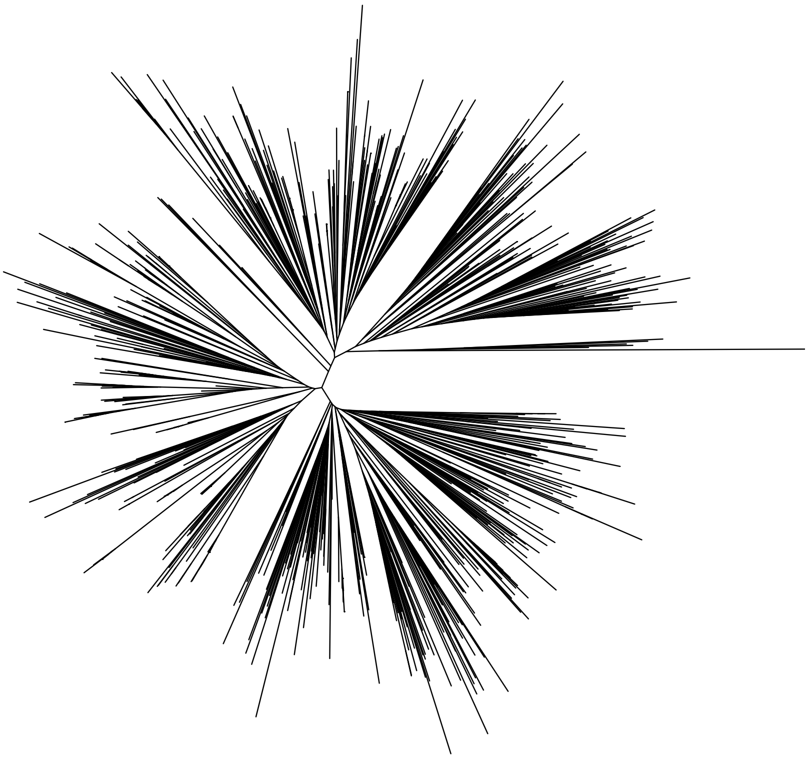

Supplementary Figure S2

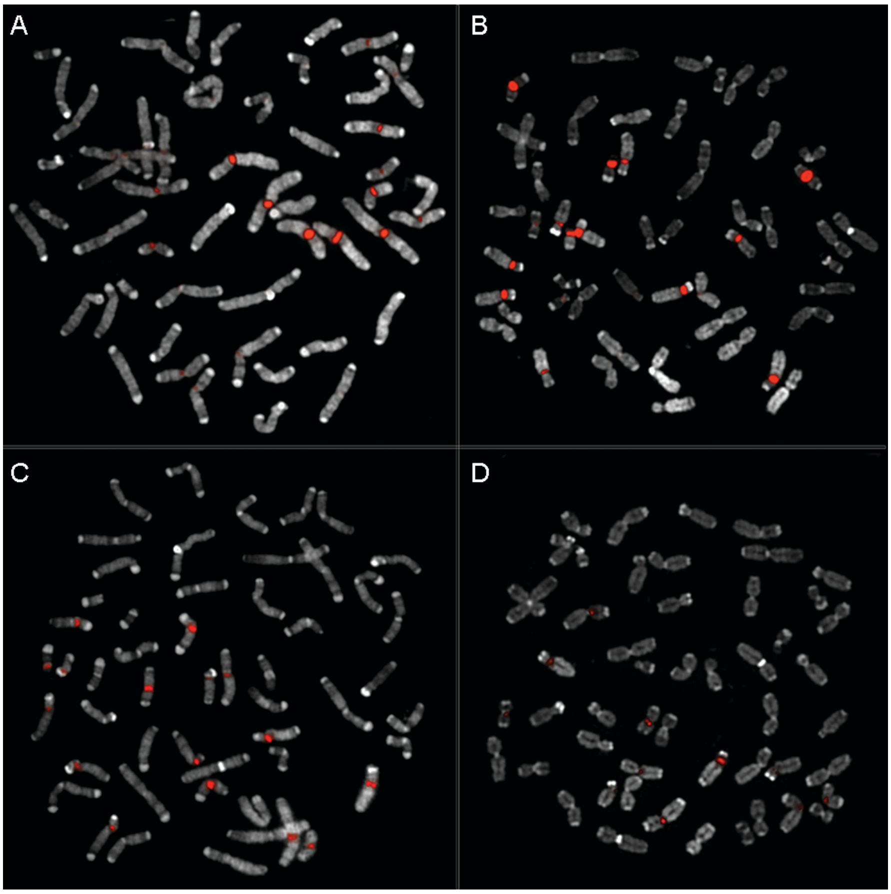

Supplementary Figure S3

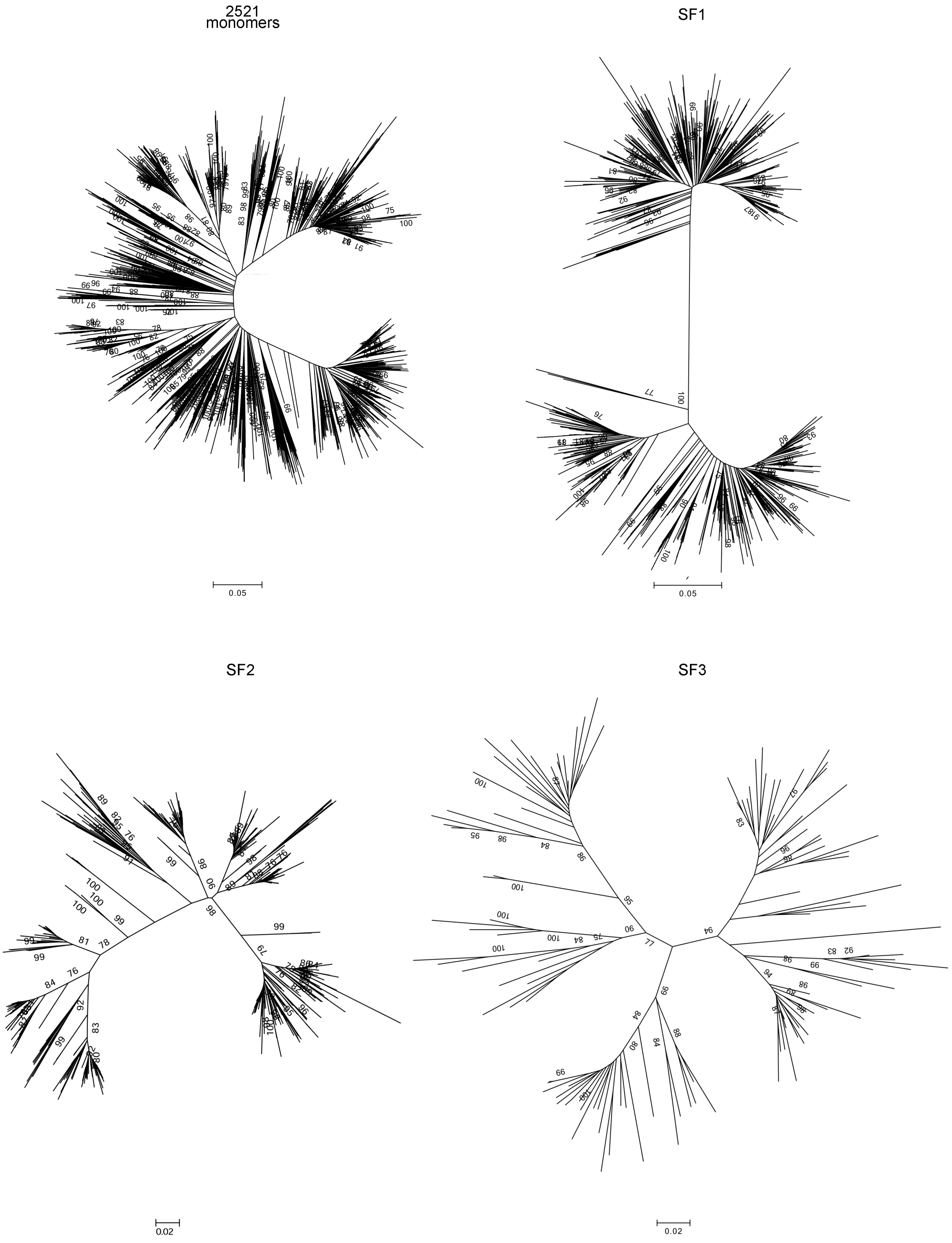

Supplementary Figure S4

Supplementary tables

Table S1. Hybridization results of 71 gorilla centromeric plasmids and 41 gorilla centromeric BACs on gorilla metaphase chromosomes, classified by hybridization pattern. "Exceptional" clones indicated below the dotted line. *In vitro* enzymatic digestion results are also shown only for BAC clones. RE= restriction enzyme.

	PLASMID	GGO chromosome																							
		1-I	2-III	3-IV	4-V[a]	5-VI	6-VII	7-VIII	8-X	9-XI	10-XII	11-IIq	12-IIp	13-IX	14-XIII	15-XV	16-XVIII	17-XVI	18-XIV	19-XVII[a]	20-XIX	21-XX	22-XXI	23-XXII	X
GROUP 1	E.5	++		+++	+++	+++	+++	++	++		++							+++			+++	+++			++
	E.50	+++		++		+++	++		+++	+++	+++							++			+++	+++			
	E.59		++					++			++											++			
	E.73	++		+++	++		+++	+++			++											++			
	F.8	+++	++	++	+++	+++	+++	+++	+++	++	+++							+++			+++	+++			++
	G.100	+++			++	+++					+++										++	+++			
	G.105	++		+++		++		+++	++	++												+++			++
	A1.36				++			++												+++	++				
	A1.41	+++	++		+++	++	++	++	++	++	++							++		++		++			++
	E.32			++							++							+++		+++					++
	E.54	+++								+++	+++							+++		++	++	++			++
GROUP 2	A1.3																			+++			++		
	A1.6												++	++			++		+++	++			++	+++	
	A1.8												++	++			++		++	+++				++	
	A1.9												+++	++	++		++			++			+++	+++	
	A1.12																		++	+++			++	++	
	A1.50											++	++		+++	+++	++		+++	+++				++	
	A1.52													++						+++					
	A1.60											++		++			++		++	+++			++	++	
	A1.62																+++		++				+++	+++	
	A1.64											++	++				++		+++	+++			+++	+++	
	A1.65															++								++	
	A2.4												++	++			++		++	+++			+++	+++	
	B1.1											++		++	++				++	+++			++	++	
	C5c													++			++		++	+++				+++	
	C7b											++	++	++			++		++	++			++	++	
	C15													++						+++			++	+++	
	C30a													++	++	++	++		++	+++			++	++	
	C35a													++			++		++	+++				++	
	C40														++	++				++			++	+	
	C41											++	++	+++	++	++	++			+++			+++	+++	
	C42b											++	++				++		++	+++			++	++	
	E.7																			+++				++	
	E.20											++		++					++	+++			++	++	
	E.24													++			++		++	+++				++	
	E.33																++		++	+++				++	
	E.34																		++	+++				++	
	E.36																			+++					
	E.56													++					++	+++			++	++	
	F.18											++	++	++	++	++	++		++	+++			++	++	
	G.84													+++					++	++			+++	++	
	A1.4				+++								++	++			++		++	+++					
	A1.18				++								++		++	++			+++						
	A1.49				++							++		++		++				++			++	++	
	A2.1				++								++	++			++		++	+++			++		
	A2.2				+++									++			+			+++			++	++	
	A2.5				++									++			++			+++			++	++	
	B1.3				+++								++	+			++		++	+++			++	++	
	B1.4				++									++	++										
	C13				++									+++		++	++			+++				+++	
	E.12				+++									++			++		++	+++			++	++	
	E.83				++							++		+++					++	++			++	++	
	F.94				+++							++		++			++			++			++		
GROUP 3	26a								+++			++	++	++						++			++	++	
	A1.7				+++	++		++				++	+++				++	+++		++		++	+++	+++	++
	A1.10		++		++	++		++				++			++						++		++	++	
	A1.19		++		++							++							++	++					
	A1.40				++		++			++			++						++	+++				++	
	A1.44	++			+++		++		++					++				+++	++		++	++	++	++	++
	A1.73				++								++	+++			+++		+++	+++	++		++	+++	++
	C8				+++	+++					+++									+++	+++	+++		+++	++
	E.27																		++	+++				++	++
	E.35									++	+++	++						++			++				++
	E.95											++		+++						+++	++			+++	
	E.104				++									++	++	++	++		++	+++	++		++	++	++
	G.18	++	+++		++	++	++	++	+++	++	++	++	++		++		++	++	++	++	++	+++	++	++	+++
	G.81		+++		+++	++														++				++	
	G.97												++	+++		++	++			+++	++		++	++	
GROUP 4	A1.15										+														
	E.1																			+					
	E.31																			+					

	BAC ID	GGO chromosome																								RE	restriction pattern		
		1-I	2-III	3-IV	4-V[a]	5-VI	6-VII	7-VIII	8-X	9-XI	10-XII	11-IIq	12-IIp	13-IX	14-XIII	15-XV	16-XVIII	17-XVI	18-XIV	19-XVII[a]	20-XIX	21-XX	22-XXI	23-XXII	X		170bp ladder	340bp ladder	higher bands
GROUP 1	CH255-54G3	+++		++		++	+++	++	++		+++							+++				++			++	NA			
	CH255-65K2	+++	++	++	++		++		+++		++										++				++	EcoRI		X	
	CH255-74N2			+++																						EcoRI		X	
	CH255-82B2	++		+++	++	++	++	++	++		++							++			++	++			++	EcoRI		X	
	CH255-90G1	++		+++	++	++	++	++	++		++							++			++	++			++	EcoRI		X	
	CH255-54A1	++			+++		++	++	+++		+++							++				++			+++	EcoRI		X	
	CH255-63P2	++					++		+++		+++															EcoRI		X	
	CH255-74D1	++	++		+++	++	+++		++	++	+++							++			++	++			++	EcoRI		X	
	CH255-77P5	+++		++	++		+++	++	++		+++							+++							++	EcoRI		X	
	CH255-72A8	+++	+++				+++		+++		++							++			+++	++			++	EcoRI		X	
	CH255-83C1	+++	+++				+++		+++		+++							+++								EcoRI		X	
	CH255-54L2	+++	+++	++			+++	++	+++		+++							++				++			++	EcoRI		X	
	CH255-94H1	+++	++	++	++	++	+++	+++										+++							++	EcoRI		X	
	CH255-69O1	++	++	++	++	++	+++	++	++		++							++			++	+++				EcoRI		X	
	CH255-75P5	+++	++	+++	+++	++	++	++			+++							++				+++				EcoRI		X	
	CH255-85J1	++		+++	++	++	++	++			+++							++				++			++	EcoRI		X	
	CH255-77K2	+++	++	++	+++	++	+++	+++	++	+++	+++							++			++	+++				EcoRI		X	
	CH255-86E1	+++	++		++	++	+++	+++	+++		+++							+++				+++				EcoRI		X	
	CH255-93P1	+++			++	++	+++		++		++											+++				EcoRI		X	
	CH255-52M	++		+++	++			++										+++								EcoRI		X	
	CH255-53A2	++		++	+++	++	++	++	++	++	++							++				+++			++	EcoRI		X	
	CH255-58E7	++		+++	++	++		++		++												++				EcoRI		X	
	CH255-62A2	++		++	++	++	++	++	++	++								++				++			++	EcoRI		X	
	CH255-68K1	++				++	++	++	++													++				EcoRI		X	
	CH255-75M	++			+++	++	+++	++	++	++	++							++			++	+++			++	EcoRI		X	
	CH255-74P7	++	++			+++	+++			++												++				EcoRI		X	
	CH255-86F1	++		+++		++	++		+++	++	+++							++				++				EcoRI		X	
	CH255-91A3	++	++	++		+++	++	++	++		+++							++				+++			+++	EcoRI		X	
	CH255-91A8	++	++	+++	++		++	++	++		++							++				+++			++	EcoRI		X	
	CH255-73I1	++	++	+++		++		++														++			++	EcoRI		X	
	CH255-77N24		+++					++	++		++											++				EcoRI		X	
	CH255-62P2	+++	++	+++	++	++	+++	++	+++	++	+++							+++			+++	++			++	EcoRI		X	
	CH255-77L1	+++	++	++	++	++	+++	++	++	++	++							++		+++	+++	+++			+++	EcoRI		X	
GROUP 2	CH255-90D8																			+++						EcoRI			7mer, 8mer, 12mer, 14mer, 16mer
	CH255-58L10																		++	+++				++		EcoRI			4mer, 6mer
	CH255-67O23											++	++	++	++	++	++		++	++			+++	++		StuI, XmnI	X		5mer, 8mer
	CH255-49C21											+++	++		++	++	++		+++	++			++	++		EcoRI	X		5mer, 7mer, 10mer, 11mer
	CH255-91G23												++	++	++		++		+++	++			++	+++		EcoRI		X	
	CH255-61G22											++	++		++	++	++		+++	++			++	++		StuI, EcoRI	X		4mer, 8mer
	CH255-73G21											+++	++		++	++	++		+++	++			++	++		XmnI, StuI, EcoRI	X		9mer, 10mer
	CH255-50P4												++	++					+++	++			+++	+++		XmnI, StuI	X		6mer, 11mer

Note. Plus represents the intensity of the detected signals: "++" medium and "+++" strong. Chromosome cells are colored based on the Group 1 and 2 hybridization patterns.

[a]Gorilla chromosomes V and XVII contain the centromeres of human chromosomes 17 and 5, respectively.

Table S2. Comparison between monomer type (A or B) based on p-distances with consensi, and PRD. Discordant results in bold.

		vs consensus A	vs consensus B	p-distance monomer type	PRD	ΔA/B-distance	|ΔA/B-distance|
1	A1.49_5	0,104	0,106	A	α	-0,002	0,002
2	G.97_6	0,104	0,106	A	α	-0,002	0,002
3	E.54_2	0,160	0,163	A	α	-0,003	0,003
4	G.97_2	0,179	0,182	A	α	-0,003	0,003
5	F.94_2	0,184	0,188	**A**	**B**	-0,003	0,003
6	A1.36F_3	0,105	0,113	A	α	-0,008	0,008
7	A1.52F_3	0,105	0,113	A	α	-0,008	0,008
8	A1.44R_3	0,153	0,163	A	α	-0,009	0,009
9	E.95_2	0,153	0,163	A	α	-0,009	0,009
10	E.104_2	0,153	0,163	A	α	-0,009	0,009
11	A1.44R_2	0,123	0,138	A	α	-0,015	0,015
12	A1.49_1	0,123	0,138	A	α	-0,015	0,015
13	A1.60_1	0,123	0,138	A	α	-0,015	0,015
14	A1.12_1	0,135	0,150	A	α	-0,015	0,015
15	E.95_1	0,153	0,169	A	α	-0,015	0,015
16	E.104_1	0,153	0,169	A	α	-0,015	0,015
17	E.83_4	0,166	0,181	A	α	-0,016	0,016
18	A1.60_3	0,160	0,176	A	α	-0,017	0,017
19	A1.50_1	0,104	0,125	A	α	-0,021	0,021
20	A1.64_1	0,106	0,127	A	α	-0,021	0,021
21	E.83_5	0,184	0,206	A	α	-0,022	0,022
22	A1.12_3	0,166	0,189	A	α	-0,023	0,023
23	G.97_3	0,086	0,113	A	α	-0,027	0,027
24	A1.36F_2	0,123	0,150	A	α	-0,027	0,027
25	A1.52F_2	0,123	0,150	A	α	-0,027	0,027
26	A1.19F_1	0,136	0,164	A	α	-0,028	0,028
27	A1.40_2	0,110	0,138	A	α	-0,028	0,028
28	E.31_3	0,129	0,157	A	α	-0,028	0,028
29	E.56_1	0,118	0,146	A	α	-0,028	0,028
30	A1.40_6	0,135	0,164	A	α	-0,029	0,029
31	E.32_2	0,124	0,153	A	α	-0,029	0,029
32	A1.44R_1	0,127	0,157	A	α	-0,029	0,029
33	A1.49_6	0,244	0,276	A	α	-0,032	0,032
34	A1.19R_3	0,099	0,132	A	α	-0,033	0,033
35	A1.44F_2	0,098	0,132	A	α	-0,034	0,034
36	F.94_3	0,104	0,138	A	α	-0,034	0,034
37	A1.40_1	0,166	0,200	A	α	-0,034	0,034
38	A1.52F_4	0,111	0,146	A	α	-0,034	0,034
39	A1.36F_4	0,117	0,152	A	α	-0,035	0,035
40	A1.36R_1	0,141	0,176	A	α	-0,035	0,035
41	A1.52R_2	0,141	0,176	A	α	-0,035	0,035
42	A1.52R_1	0,125	0,166	A	α	-0,041	0,041
43	A1.40_5	0,117	0,157	A	α	-0,041	0,041
44	E.56_2	0,117	0,157	A	α	-0,041	0,041
45	C8_1	0,155	0,196	A	α	-0,041	0,041
46	E.32_3	0,129	0,170	A	α	-0,041	0,041
47	A1.19R_1	0,141	0,182	A	α	-0,041	0,041
48	E.12_2	0,141	0,182	A	α	-0,041	0,041
49	F.94_1	0,141	0,182	A	α	-0,041	0,041
50	A1.52R_5	0,266	0,312	A	α	-0,046	0,046
51	A1.73_2	0,080	0,126	A	α	-0,046	0,046
52	E.54_1	0,104	0,151	A	α	-0,047	0,047
53	A1.52F_5	0,117	0,164	A	α	-0,047	0,047
54	E.27_3	0,178	0,226	A	α	-0,049	0,049
55	G.81_1	0,098	0,151	A	α	-0,053	0,053
56	E.83_3	0,136	0,190	A	α	-0,054	0,054
57	G.84_4	0,148	0,203	A	α	-0,054	0,054
58	E.24_2	0,129	0,189	A	α	-0,060	0,060
59	A1.50_3	0,135	0,195	A	α	-0,060	0,060
60	A1.64_3	0,135	0,195	A	α	-0,060	0,060
61	E.7_2	0,135	0,195	A	α	-0,060	0,060
62	A1.44F_4	0,074	0,138	A	α	-0,065	0,065
63	E.32_1	0,092	0,157	A	α	-0,065	0,065
64	F.18F_1	0,098	0,164	A	α	-0,065	0,065
65	A1.40_4	0,104	0,170	A	α	-0,066	0,066
66	E.59_1	0,141	0,208	A	α	-0,066	0,066
67	G.18_5	0,141	0,208	A	α	-0,066	0,066
68	G.97_1	0,147	0,214	A	α	-0,067	0,067
69	F.18F_4	0,160	0,226	A	α	-0,067	0,067
70	G.100_1	0,123	0,195	A	α	-0,072	0,072
71	G.84_1	0,147	0,220	A	α	-0,073	0,073
72	G.100_5	0,153	0,226	A	α	-0,073	0,073
73	E.33_1	0,117	0,195	A	α	-0,078	0,078
74	G.18_1	0,104	0,189	A	α	-0,084	0,084
75	A1.12_6	0,123	0,208	A	α	-0,085	0,085
76	A1.40_7	0,123	0,208	A	α	-0,085	0,085
77	A1.60_6	0,123	0,208	A	α	-0,085	0,085
78	F.18R_3	0,135	0,220	A	α	-0,085	0,085
79	G.100_3	0,098	0,189	A	α	-0,091	0,091
80	G.18_3	0,110	0,201	A	α	-0,091	0,091
81	E.5_1	0,123	0,214	A	α	-0,091	0,091
82	E.35_1	0,123	0,214	A	α	-0,091	0,091
83	E.50_1	0,123	0,215	A	α	-0,092	0,092
84	F.8_3	0,160	0,252	A	α	-0,092	0,092
85	E.5_3	0,098	0,201	A	α	-0,103	0,103

		vs consensus A	vs consensus B	p-distance monomer type	PRD	ΔA/B-distance	|ΔA/B-distance|
86	G.105_6	0,118	0,223	A	α	-0,105	0,105
87	E.73_6	0,113	0,218	A	α	-0,105	0,105
88	E.73_2	0,110	0,220	A	α	-0,110	0,110
89	F.8_1	0,110	0,220	A	α	-0,110	0,110
90	G.105_2	0,110	0,220	A	α	-0,110	0,110
91	F.8_5	0,117	0,226	A	α	-0,110	0,110
92	E.73_4	0,129	0,239	A	α	-0,110	0,110
93	G.105_4	0,129	0,239	A	α	-0,110	0,110
1	E.5_2	0,194	0,069	B	B	0,125	0,125
2	F.8_2	0,181	0,056	B	B	0,125	0,125
3	E.73_3	0,206	0,081	B	B	0,125	0,125
4	G.105_3	0,206	0,081	B	B	0,125	0,125
5	F.8_4	0,194	0,069	B	B	0,125	0,125
6	E.73_1	0,225	0,106	B	B	0,119	0,119
7	G.105_1	0,225	0,106	B	B	0,119	0,119
8	E.73_5	0,189	0,075	B	B	0,113	0,113
9	G.18_2	0,213	0,100	B	B	0,113	0,113
10	G.105_5	0,188	0,075	B	B	0,113	0,113
11	G.100_2	0,225	0,119	B	B	0,106	0,106
12	G.100_6	0,244	0,138	B	B	0,106	0,106
13	A1.12_2	0,239	0,144	B	B	0,096	0,096
14	A1.60_2	0,199	0,106	B	B	0,093	0,093
15	A1.73_3	0,198	0,106	B	B	0,092	0,092
16	E.24_1	0,198	0,106	B	B	0,092	0,092
17	E.34_1	0,198	0,106	B	B	0,092	0,092
18	A1.50_2	0,204	0,112	B	B	0,092	0,092
19	A1.64_2	0,204	0,112	B	B	0,092	0,092
20	E.7_1	0,210	0,118	B	B	0,092	0,092
21	A1.50_5	0,203	0,114	B	no box	0,089	0,089
22	A1.64_5	0,203	0,114	B	no box	0,089	0,089
23	A1.12_4	0,209	0,120	B	B	0,089	0,089
24	A1.60_4	0,209	0,120	B	no box	0,089	0,089
25	E.36_1	0,228	0,143	B	B	0,086	0,086
26	F.18F_3	0,228	0,143	B	B	0,086	0,086
27	A1.12_5	0,203	0,127	B	B	0,076	0,076
28	A1.60_5	0,203	0,127	B	B	0,076	0,076
29	F.18R_1	0,184	0,108	B	B	0,076	0,076
30	F.18R_2	0,196	0,120	B	B	0,076	0,076
31	E.35_2	0,175	0,100	B	B	0,075	0,075
32	G.100_4	0,206	0,131	B	B	0,075	0,075
33	F.18F_5	0,186	0,113	B	B	0,074	0,074
34	A1.50_4	0,173	0,106	B	B	0,067	0,067
35	A1.64_4	0,173	0,106	B	B	0,067	0,067
36	A1.40_8	0,185	0,118	B	B	0,067	0,067
37	A1.36F_1	0,191	0,124	B	B	0,067	0,067
38	A1.52F_1	0,191	0,124	B	B	0,067	0,067
39	F.18R_4	0,191	0,124	B	B	0,067	0,067
40	A1.19F_3	0,191	0,130	B	B	0,061	0,061
41	F.18F_2	0,167	0,112	B	B	0,055	0,055
42	E.1_2	0,130	0,081	B	B	0,049	0,049
43	E.83_1	0,198	0,149	B	B	0,048	0,048
44	E.27_1	0,216	0,168	B	B	0,048	0,048
45	A1.40_3	0,168	0,125	B	B	0,043	0,043
46	E.27_2	0,154	0,112	B	B	0,043	0,043
47	A1.36R_3	0,188	0,151	B	B	0,037	0,037
48	A1.52R_4	0,188	0,151	B	B	0,037	0,037
49	E.83_2	0,161	0,125	B	B	0,036	0,036
50	A1.19F_2	0,216	0,180	B	B	0,036	0,036
51	G.18_4	0,216	0,180	B	B	0,036	0,036
52	G.84_3	0,222	0,186	B	B	0,036	0,036
53	G.97_4	0,141	0,106	**B**	**α**	0,035	0,035
54	A1.36R_2	0,118	0,088	B	B	0,031	0,031
55	A1.52R_3	0,118	0,088	B	B	0,031	0,031
56	A1.44F_5	0,142	0,112	B	B	0,030	0,030
57	A1.73_1	0,179	0,149	B	B	0,030	0,030
58	F.94_4	0,179	0,149	B	B	0,030	0,030
59	E.12_1	0,191	0,161	B	B	0,030	0,030
60	E.31_1	0,141	0,113	**B**	**α**	0,029	0,029
61	A1.49_3	0,153	0,125	**B**	**α**	0,028	0,028
62	G.84_2	0,160	0,137	B	B	0,024	0,024
63	E.31_2	0,167	0,143	B	no box	0,024	0,024
64	A1.19R_2	0,147	0,125	B	B	0,022	0,022
65	E.12_3	0,147	0,125	B	B	0,022	0,022
66	A1.19R_4	0,152	0,134	B	B	0,018	0,018
67	A1.44F_3	0,185	0,168	B	B	0,017	0,017
68	E.1_1	0,183	0,169	**B**	**α**	0,014	0,014
69	A1.44F_1	0,194	0,181	B	B	0,013	0,013
70	G.97_5	0,166	0,154	B	no box	0,012	0,012
71	A1.49_2	0,142	0,130	B	B	0,012	0,012
72	A1.50_6	0,374	0,364	B	B	0,010	0,010
73	A1.64_6	0,373	0,364	B	B	0,010	0,010
74	A1.44R_4	0,166	0,156	**B**	**α**	0,009	0,009
75	A1.49_4	0,146	0,141	B	no box	0,005	0,005
76	E.95_3	0,166	0,163	**B**	**α**	0,003	0,003
77	E.104_3	0,166	0,163	B	**α**	0,003	0,003

Table S3a. Centromeric sequences from SF1-3 WGSS analysis by Tandem Repeats Finder, JDotter, RepeatMasker and phylogenetic tree observation.

WGSS accession number	size (bp)	definition	TRF[a]		dot plot periodicity	Repeat Masker	NEB Cutter		GGO suprachromosomal family[b]
			highest score periods	period score			Restriction Enzyme	fragment size (bp)	
CABD02041326	1627	g. gorilla gorilla wgss assembly, chr10_41717784_2087598.492	2mers/4mers	2989/2998	2mers	alpha-satellite	EcoRI	340	SF1
CABD02042655	14275	g. gorilla gorilla wgss assembly, chr10_41847028_9490812.332	2mers/6mers	24743/24743	2mers	alpha-satellite	EcoRI	340	SF1
CABD02042717	7160	g. gorilla gorilla wgss assembly, chr10_41847028_9490812.394	2mers/6mers/8mers	12615/12680/12675	2mers	alpha-satellite	EcoRI	340	SF1
CABD02042904	21190	g. gorilla gorilla wgss assembly, chr10_41847028_9490812.581	2mers	28952/8409	2mers	alpha-satellite	EcoRI	340	SF1
CABD02043063	17680	g. gorilla gorilla wgss assembly, chr10_41847028_9490812.740	2mers/8mers	31529/31564	2mers	alpha-satellite	EcoRI	340	SF1
CABD02043194	6530	g. gorilla gorilla wgss assembly, chr10_41847028_9490812.871	2mers	10948	2mers	alpha-satellite	EcoRI	340	SF1
CABD02043380	4560	g. gorilla gorilla wgss assembly, chr10_41847028_9490812.1057	2mers/10mers	8118/8299	2mers	alpha-satellite	EcoRI	340	SF1
CABD02043685	5908	g. gorilla gorilla wgss assembly, chr10_41847028_9490812.1362	2mers	10447	2mers	alpha-satellite	EcoRI	340	SF1
CABD02043690	7031	g. gorilla gorilla wgss assembly, chr10_41847028_9490812.1367	2mers	10454	2mers	alpha-satellite	EcoRI	340	SF1
CABD02043710	2224	g. gorilla gorilla wgss assembly, chr10_41847028_9490812.1387	2mers	4038	2mers	LTR to 264bp; alpha-satellite from 790bp	EcoRI	340	SF1
CABD02043744	16009	g. gorilla gorilla wgss assembly, chr10_41847028_9490812.1421	2mers/4mers	27332/27362	2mers	alpha-satellite	EcoRI	340	SF1
CABD02043759	6667	g. gorilla gorilla wgss assembly, chr10_41847028_9490812.1436	2mers/10mers	11514/11572	2mers	alpha-satellite	EcoRI	340	SF1
CABD02043767	15837	g. gorilla gorilla wgss assembly, chr10_41847028_9490812.1444	2mers	28408	2mers	alpha-satellite	EcoRI	340	SF1
CABD02043786	13314	g. gorilla gorilla wgss assembly, chr10_41847028_9490812.1463	2mers/6mers	23624/23629	2mers	alpha-satellite	EcoRI	340	SF1
CABD02340093	15916	g. gorilla gorilla wgss assembly, chr7_61604593_210427.19	2mers	19468	2mers	alpha-satellite	EcoRI	340	SF1
CABD02378036	3795	g. gorilla gorilla wgss assembly, chr9_38991108_196794.5	2mers/4mers	3438/3462	2mers	1419bp SINEs and LTR/ERVL-MaLR; alpha-satellite from 1818bp	EcoRI	340	SF1
CABD02105058	26052	g. gorilla gorilla wgss assembly, chr14_18070001_78812.2	monomers/2mers/8mers	21504/32548/34152	8mers	alpha-satellite	EcoRI	1382	SF2[c]
CABD02111890	21680	g. gorilla gorilla wgss assembly, chr14_68153186_1675064.6	monomers/2mers/8mers	4919/8301/11514	8mers	alpha-satellite to 8752bp, SINEs and LTR (DNA/TcMar-Tigger)	EcoRI	1382	SF2[c]
CABD02162145	2958	g. gorilla gorilla wgss assembly, chr18_16764900_2443665.288	monomers/4mers/5mers	3772/3914/4107	monomers	alpha-satellite	HinfI	171	SF2
CABD02162533	2361	g. gorilla gorilla wgss assembly, chr18_16764900_2443665.676	monomers/3mers	3112/3324	3mers	alpha-satellite	HinfI	171	SF2
CABD02196935	15154	g. gorilla gorilla wgss assembly, chr2_91548939_505318.48	monomers/8mers	17450/17912	8mers	13812bp alpha-satellite, SINEs and LINEs	EcoRI	1389	SF2[c]
CABD02196940	13625	g. gorilla gorilla wgss assembly, chr2_91548939_505318.53	monomers	13741	monomers	alpha-satellite	EcoRI	1390	SF2[c]
CABD02196967	8498	g. gorilla gorilla wgss assembly, chr2_91548939_505318.80	monomers/2mers/8mers	6564/7240/12853	8mers	alpha-satellite	EcoRI	1382	SF2[c]
CABD02219203	9989	g. gorilla gorilla wgss assembly, chr20_26077633_1186791.127	monomers/2mers/3mers/9m	12012/1742/1994/12704	9mers	alpha-satellite	EcoRI	1382/1539	SF2[c]
CABD02339997	1532	g. gorilla gorilla wgss assembly, chr7_61082970_61304.10	monomers/3mers	1987/2213	monomers	alpha-satellite	HinfI	171	SF2
CABD02378856	17036	g. gorilla gorilla wgss assembly, chr9_66508401_884849.28	monomers	20066	8mers	alpha-satellite	EcoRI	1382	SF2[c]
CABD02062797	1584	g. gorilla gorilla wgss assembly, chr11_51279207_195064.37	monomers/4mers	2030/2716	4mers	alpha-satellite	HinfI	171	SF3
CABD02062798	2585	g. gorilla gorilla wgss assembly, chr11_51279207_195064.38	monomers/7mers	3332/4243	monomers	alpha-satellite	HinfI	171	SF3
CABD02399115	1882	g. gorilla gorilla wgss assembly, chrX_58464962_182448.58	monomers/5mers	2347/3045	5mers	alpha-satellite	HinfI	171	SF3
CABD02399213	4356	g. gorilla gorilla wgss assembly, chrX_61625820_722115.92	monomers/4mers/8mers	5673/7609/7614	4mers	alpha-satellite	HinfI	171/342	SF3

[a]Alignment parameters (match, mismatch, indels) 2, 3, 5; min alignment score to report repeat 50; max period size 2000.
[b]According to the distribution of monomers in the phylogenetic tree.
[c]Sequences with extra long monomers.

Table S3b. Centromeric sequences from WGSS analysis by Tandem Repeats Finder, JDotter, RepeatMasker and phylogenetic tree observation.

WGSS accession number	size (bp)	definition	TRF[a]		dot plot periodicity	Repeat Masker	NEB Cutter		GGO suprachromosomal family[b]
			highest score periods	period score			Restriction Enzyme	fragment size (bp)	
CABD02062338	1592	g. gorilla gorilla wgss assembly, chr11_48761406_142758.12	monomers/2mers	2391/2399	monomers	alpha-satellite	HinfI	171	other
CABD02062339	1487	g. gorilla gorilla wgss assembly, chr11_48761406_142758.13	monomers	2332	monomers	alpha-satellite	HinfI	171	other
CABD02062342	1293	g. gorilla gorilla wgss assembly, chr11_48761406_142758.16	monomers	1581	monomers	alpha-satellite	HinfI	171	other
CABD02062800	14681	g. gorilla gorilla wgss assembly, chr11_51279207_195064.40	monomers/4mers/5mers	19144/19220/19009	4mers	alpha-satellite	HinfI	171/342	other
CABD02074388	2471	g. gorilla gorilla wgss assembly, chr12_4869828_2198150.20	monomers/3mers	2838/2838	monomers	alpha-satellite to 1894bp, LINEs	HinfI	171	other
CABD02078542	2561	g. gorilla gorilla wgss assembly, chr12_34721984_101804.10	2mers/3mers/5mers	3308/3355/3791	monomers	alpha-satellite	HinfI	171	other
CABD02078556	6154	g. gorilla gorilla wgss assembly, chr12_34721984_101804.24	monomers/3mers	8375/8367	monomers	alpha-satellite	HinfI/HaeIII	171	other
CABD02136827	2125	g. gorilla gorilla wgss assembly, chr16_34823341_347528.66	monomers/3mers/4mers	3338/3362/3471	monomers	alpha-satellite	HinfI/HaeIII	171	other
CABD02148649	2841	g. gorilla gorilla wgss assembly, chr17_21607211_684824.115	monomers/2mers	891/3130	2mers	alpha-satellite	HinfI	171	other
CABD02177216	1990	g. gorilla gorilla wgss assembly, chr19_32424770_236691.8	monomers/2mers/3mers	3035/3161/3046	monomers	alpha-satellite	XmnI	171	other
CABD02177245	1455	g. gorilla gorilla wgss assembly, chr19_32424770_236691.37	monomers/2mers	1813/1163	monomers	alpha-satellite	HinfI	171	other
CABD02248520	6149	g. gorilla gorilla wgss assembly, chr3_90496922_160426.11	monomers/6mers	9031/9093	monomers	alpha-satellite	EcoRI	681	other
CABD02248526	3033	g. gorilla gorilla wgss assembly, chr3_90496922_160426.17	monomers/7mers	4337/4467	monomers	alpha-satellite	EcoRI	1020	other
CABD02248536	2010	g. gorilla gorilla wgss assembly, chr3_90496922_160426.27	monomers/2mers	2857/2877	monomers	alpha-satellite	HinfI	171	other
CABD02248539	1629	g. gorilla gorilla wgss assembly, chr3_90496922_160426.30	monomers/3mers	2372/2440	monomers	alpha-satellite	HaeIII	171	other
CABD02248547	2059	g. gorilla gorilla wgss assembly, chr3_90496922_160426.38	monomers/3mers/4mers	2962/2979/3155	monomers	alpha-satellite	HaeIII	171	other
CABD02293322	1581	g. gorilla gorilla wgss assembly, chr5_46341207_144687.14	monomers/3mers	2423/2500	monomers	alpha-satellite	HinfI	171	other
CABD02293327	1698	g. gorilla gorilla wgss assembly, chr5_46341207_144687.19	monomers	2627	monomers	alpha-satellite	HinfI	171	other
CABD02293328	1851	g. gorilla gorilla wgss assembly, chr5_46341207_144687.20	monomers	2848	monomers	alpha-satellite	HinfI	171	other
CABD02293335	2172	g. gorilla gorilla wgss assembly, chr5_46341207_144687.27	monomers/4mers	3067/3127	monomers	alpha-satellite	HinfI	171	other
CABD02293349	3588	g. gorilla gorilla wgss assembly, chr5_46341207_144687.41	monomers/3mers	5230/5435	monomers	alpha-satellite	XmnI	171	other
CABD02293355	2001	g. gorilla gorilla wgss assembly, chr5_46341207_144687.47	monomers/2mers/3mers	2970/2971/2986	monomers	alpha-satellite	HinfI	171	other
CABD02318489	2235	g. gorilla gorilla wgss assembly, chr6_61938287_427991.31	monomers	3488	monomers	alpha-satellite	HinfI	171	other
CABD02318490	2257	g. gorilla gorilla wgss assembly, chr6_61938287_427991.32	monomers/4mers	3528/3542	monomers	alpha-satellite	HinfI	171	other
CABD02318497	1911	g. gorilla gorilla wgss assembly, chr6_61938287_427991.39	monomers/4mers	2790/2842	monomers	alpha-satellite	HaeIII	171	other
CABD02340102	15367	g. gorilla gorilla wgss assembly, chr7_61604593_210427.28	monomers/2mers	14027/14334	monomers	Alu from 14021-14327bp, alpha-satellite	HinfI/HaeIII	171	other
CABD02378835	3001	g. gorilla gorilla wgss assembly, chr9_66508401_884849.7	monomers/2mers/3mers	4323/4253/4394	monomers	alpha-satellite	HaeIII	171	other
CABD02379440	6003	g. gorilla gorilla wgss assembly, chr9_69218326_276768.2	monomers/5mers	3755/4053	monomers	SINEs and LTR; alpha-satellite from 2673bp	HinfI	171	other
CABD02399108	1830	g. gorilla gorilla wgss assembly, chrX_58464962_182448.51	monomers/3mers/4mers	2617/2716/2714	monomers	alpha-satellite	HinfI/HaeIII	171	other
CABD02399127	9254	g. gorilla gorilla wgss assembly, chrX_61625820_722115.6	monomers/5mers/6mers	13661/13691/13816	5mers	alpha-satellite	HinfI	171	other
CABD02399145	2793	g. gorilla gorilla wgss assembly, chrX_61625820_722115.24	monomers/4mers/6mers	3911/4091/4677	6mers	alpha-satellite	EcoRI	340	other
CABD02399170	13718	g. gorilla gorilla wgss assembly, chrX_61625820_722115.49	monomers/2mers/3mers	6914/16094/16789	monomers	alpha-satellite	HinfI/HaeIII	171	other
CABD02399207	6717	g. gorilla gorilla wgss assembly, chrX_61625820_722115.86	monomers/3mers	9441/9499	monomers	alpha-satellite	HinfI	171	other
CABD02399209	4804	g. gorilla gorilla wgss assembly, chrX_61625820_722115.88	monomers/4mers	7179/7289	monomers	alpha-satellite	HinfI	171	other
CABD02399223	6933	g. gorilla gorilla wgss assembly, chrX_61625820_722115.102	monomers/9mers	10421/10679	monomers	alpha-satellite	HinfI	171	other
CABD02399238	3932	g. gorilla gorilla wgss assembly, chrX_61625820_722115.117	monomers/9mers	5954/6739	9mers	alpha-satellite	HinfI	171	other

[a]Alignment parameters (match, mismatch, indels) 2, 3, 5; min alignment score to report repeat 50; max period size 2000.
[b]According to the distribution of monomers in the phylogenetic tree.

Table S4. Comparison between FISH and phylogenetic analyses on the plasmid pool analyzed. "Exceptional" clones indicated below the dotted line.

sequence source	sequence ID	units	dot plot periodicity	restriction enzyme periodicity	TRF[a]	Blast-2-sequences	p-distances vs gorilla SF consensuses	p-distances vs human SF consensuses	FISH pattern	assignment
plasmids	A1.12	6	monomers	171	monomers	monomers	SF2	SF2	SF2	SF2
	A1.19F	3	monomers	171	monomers	monomers	mix	SF4/SF5	mix	monomeric
	A1.19R	4	monomers	171	monomers	monomers	mix	SF5	mix	monomeric
	A1.36F	4	monomers	171	monomers	monomers	monomeric	SF5	SF1exceptional	monomeric
	A1.36R	3	monomers	171	monomers	monomers	monomeric	SF4/SF5	SF1exceptional	monomeric
	A1.40	8	monomers	171	monomers	monomers	mix	mix	mix	monomeric
	A1.44F	5	monomers	171	monomers	monomers	monomeric	SF5	mix	monomeric
	A1.44R	4	monomers	171	monomers	monomers	monomeric	SF5	mix	monomeric
	A1.49	6	monomers	171	monomers	monomers	monomeric	SF5	SF2exceptional	monomeric
	A1.50	6	monomers	171	monomers	monomers	SF2	SF2	SF2	SF2
	A1.52F	5	monomers	171	monomers	monomers	monomeric	SF5	SF2	-
	A1.52R	5	monomers	171	monomers	monomers	monomeric	SF4/SF5	SF2	-
	A1.60	6	monomers	171	monomers	monomers	SF2	SF2	SF2	SF2
	A1.64	6	monomers	171	monomers	monomers	SF2	SF2	SF2	SF2
	A1.73	3	monomers	171	monomers	monomers	mix	mix	mix	monomeric
	C8	1	monomers	171	monomers	monomers	monomeric	SF5	mix	monomeric
	E.1	2	monomers	171	monomers	monomers	monomeric	SF5	pericentromeric	monomeric
	E.5	3	2mers	340	monomers/2mers	dimers	SF1	SF1/SF5	SF1	SF1
	E.7	2	monomers	171	monomers	monomers	SF2	SF2	SF2	SF2
	E.12	3	monomers	171	monomers	monomers	monomeric	SF5	SF2exceptional	monomeric
	E.24	2	monomers	171	monomers	monomers	SF2	SF2	SF2	SF2
	E.27	3	monomers	171	monomers	monomers	SF3	mix	mix	SF3
	E.31	3	monomers	171	monomers	monomers	monomeric	SF5	pericentromeric	monomeric
	E.32	3	monomers	171	monomers	monomers	monomeric	SF5	SF1exceptional	monomeric
	E.33	1	monomers	171	monomers	monomers	SF2/monomeric	SF5	SF2	SF2
	E.34	1	monomers	171	monomers	monomers	SF2	mix	SF2	SF2
	E.35	2	monomers	171	monomers	monomers	mix	mix	mix	monomeric
	E.36	1	monomers	171	monomers	monomers	SF2	SF2	SF2	SF2
	E.50	1	monomers	171	monomers	monomers	SF1	SF1	SF1	SF1
	E.54	2	monomers	171	monomers	monomers	monomeric	SF5	SF1exceptional	monomeric
	E.56	2	monomers	171	monomers	monomers	monomeric	SF5	SF2	-
	E.59	1	monomers	171	monomers	monomers	SF1	SF1	SF1	SF1
	E.73	6	2mers	340	2mers	dimers	SF1	SF1	SF1	SF1
	E.83	5	monomers	171	monomers	monomers	monomeric	SF4/SF5	SF2exceptional	monomeric
	E.95	3	monomers	171	monomers	monomers	monomeric	SF5	mix	monomeric
	E.104	3	monomers	171	monomers	monomers	monomeric	SF5	mix	monomeric
	F.8	5	2mers	340	2mers	dimers	SF1	SF1	SF1	SF1
	F.18F	5	monomers	171	monomers	monomers	mix	mix	SF2	SF2
	F.18R	4	monomers	171	monomers	monomers	SF2	SF2	SF2	SF2
	F.94	4	monomers	171	monomers	monomers	monomeric	SF5	SF2exceptional	monomeric
	G.18	5	monomers	171	monomers	monomers	mix	SF1/SF2	mix	SF1/SF2
	G.81	1	monomers	171	monomers	monomers	monomeric	SF5	mix	monomeric
	G.84	4	monomers	171	monomers	monomers	SF2	SF2/SF5	SF2	SF2
	G.97	6	monomers	171	monomers	monomers	monomeric	SF5	mix	monomeric
	G.100	6	monomers	340	monomers/2mers	dimers	SF1	SF1	SF1	SF1
	G.105	6	2mers	340	2mers	dimers	SF1	SF1	SF1	SF1
WGSSs	CABD02041326	8	2mers	340	2mers/4mers	NA	SF1	SF1	-	SF1
	CABD02042655	83	2mers	340	2mers/6mers	NA	SF1	SF1	-	SF1
	CABD02042717	42	2mers	340	2mers/6mers/8mers	NA	SF1	SF1	-	SF1
	CABD02042904	124	2mers	340	2mers	NA	SF1	SF1	-	SF1
	CABD02043063	103	2mers	340	2mers/8mers	NA	SF1	SF1	-	SF1
	CABD02043194	36	2mers	340	2mers	NA	SF1	SF1	-	SF1
	CABD02043380	26	2mers	340	2mers/10mers	NA	SF1	SF1	-	SF1
	CABD02043685	34	2mers	340	2mers	NA	SF1	SF1	-	SF1
	CABD02043690	36	2mers	340	2mers	NA	SF1	SF1	-	SF1
	CABD02043710	12	2mers	340	2mers	NA	SF1	SF1	-	SF1
	CABD02043744	93	2mers	340	2mers/4mers	NA	SF1	SF1	-	SF1
	CABD02043759	38	2mers	340	2mers/10mers	NA	SF1	SF1	-	SF1
	CABD02043767	92	2mers	340	2mers	NA	SF1	SF1	-	SF1
	CABD02043786	77	2mers	340	2mers/6mers	NA	SF1	SF1	-	SF1
	CABD02340093	92	2mers	340	2mers	NA	SF1	SF1	-	SF1
	CABD02378036	10	2mers	340	2mers/4mers	NA	SF1	SF1	-	SF1
	CABD02105058	147	8mers	1382	monomers/2mers/8mers	NA	SF2	SF2	-	SF2
	CABD02111890	49	8mers	1382	monomers/2mers/8mers	NA	SF2	SF2	-	SF2
	CABD02162145	16	monomers	171	monomers/4mers/5mers	NA	SF2	SF2	-	SF2
	CABD02162533	13	3mers	171	monomers/3mers	NA	SF2	SF2	-	SF2
	CABD02196935	79	8mers	1389	monomers/8mers	NA	SF2	SF2	-	SF2
	CABD02196940	77	monomers	1390	monomers	NA	SF2	SF2	-	SF2
	CABD02196967	48	8mers	1382	monomers/2mers/8mers	NA	SF2	SF2	-	SF2
	CABD02219203	57	9mers	1382/1539	monomers/2mers/3mers/9mers	NA	SF2	SF2	-	SF2
	CABD02339997	8	monomers	171	monomers/3mers	NA	SF2	SF2	-	SF2
	CABD02378856	98	8mers	1382	monomers	NA	SF2	SF2	-	SF2
	CABD02062797	8	4mers	171	monomers/4mers	NA	SF3	SF3	-	SF3
	CABD02062798	15	monomers	171	monomers/7mers	NA	SF3	SF3	-	SF3
	CABD02062800	85	4mers	171/342	monomers/4mers/5mers	NA	SF3+monomeric	SF3/SF5	-	SF3
	CABD02399115	10	5mers	171	monomers/5mers	NA	SF3	SF3	-	SF3
	CABD02399213	25	4mers	171/342	monomers/4mers/8mers	NA	SF3	SF3	-	SF3
	CABD02062338	8	monomers	171	monomers/2mers	NA	monomeric	SF5	-	monomeric
	CABD02062339	8	monomers	171	monomers	NA	monomeric	SF5	-	monomeric
	CABD02062342	6	monomers	171	monomers	NA	monomeric	SF5	-	monomeric
	CABD02074388	11	monomers	171	monomers/3mers	NA	monomeric	SF5	-	monomeric
	CABD02078542	14	monomers	171	2mers/3mers/5mers	NA	monomeric	SF5	-	monomeric
	CABD02078556	35	monomers	171	monomers/3mers	NA	monomeric	SF5	-	monomeric
	CABD02136827	12	monomers	171	monomers/3mers/4mers	NA	monomeric	SF5	-	monomeric
	CABD02148649	16	2mers	171	monomers/2mers	NA	SF3	SF3/SF5	-	monomeric
	CABD02177216	10	monomers	171	monomers/2mers/3mers	NA	monomeric	SF5	-	monomeric
	CABD02177245	8	monomers	171	monomers/2mers	NA	monomeric	SF4	-	monomeric
	CABD02248520	35	monomers	681	monomers/6mers	NA	monomeric	SF5	-	monomeric
	CABD02248526	17	monomers	1020	monomers/7mers	NA	monomeric	SF5	-	monomeric
	CABD02248536	11	monomers	171	monomers/2mers	NA	monomeric	SF5	-	monomeric
	CABD02248539	9	monomers	171	monomers/3mers	NA	monomeric	SF5	-	monomeric
	CABD02248547	11	monomers	171	monomers/3mers/4mers	NA	monomeric	SF5	-	monomeric
	CABD02293322	8	monomers	171	monomers/3mers	NA	monomeric	SF5	-	monomeric
	CABD02293327	9	monomers	171	monomers	NA	monomeric	SF5	-	monomeric
	CABD02293328	10	monomers	171	monomers	NA	monomeric	SF5	-	monomeric
	CABD02293335	12	monomers	171	monomers/4mers	NA	monomeric	SF4/SF5	-	monomeric
	CABD02293349	20	monomers	171	monomers/3mers	NA	monomeric	SF5	-	monomeric
	CABD02293355	11	monomers	171	monomers/2mers/3mers	NA	monomeric	SF5	-	monomeric
	CABD02318489	13	monomers	171	monomers	NA	monomeric	SF5	-	monomeric
	CABD02318490	12	monomers	171	monomers/4mers	NA	monomeric	SF5	-	monomeric
	CABD02318497	10	monomers	171	monomers/4mers	NA	monomeric	SF5	-	monomeric
	CABD02340102	88	monomers	171	monomers/2mers	NA	monomeric	SF5	-	monomeric
	CABD02378835	16	monomers	171	monomers/2mers/3mers	NA	monomeric	SF5	-	monomeric
	CABD02379440	18	monomers	171	monomers/5mers	NA	monomeric	SF4	-	monomeric
	CABD02399108	10	monomers	171	monomers/3mers/4mers	NA	SF3+monomeric	SF5	-	monomeric
	CABD02399127	54	5mers	171	monomers/5mers/6mers	NA	monomeric	SF5	-	monomeric
	CABD02399145	16	6mers	340	monomers/4mers/6mers	NA	monomeric	SF5	-	monomeric
	CABD02399170	73	monomers	171	monomers/2mers/3mers	NA	SF3+monomeric	SF5	-	monomeric
	CABD02399207	38	monomers	171	monomers/3mers	NA	monomeric	SF5	-	monomeric
	CABD02399209	27	monomers	171	monomers/4mers	NA	SF3+monomeric	SF3/SF5	-	monomeric
	CABD02399223	40	monomers	171	monomers/9mers	NA	monomeric	SF5	-	monomeric
	CABD02399238	22	9mers	171	monomers/9mers	NA	monomeric	SF5	-	monomeric

[a]Alignment parameters (match, mismatch, indels) 2, 3, 5; min alignment score to report repeat 50; max period size 2000.

Table S5. Comparison between FISH and phylogenetic analyses on the plasmid pool analyzed. "Exceptional" clones indicated below the dotted line.

	PLASMID	monomer number	GGO chromosome																							gorilla suprachromosomal family (SF)[b]
			1-I	2-III	3-IV	4-V[a]	5-VI	6-VII	7-VIII	8-X	9-XI	10-XII	11-IIq	12-IIp	13-IX	14-XIII	15-XV	16-XVIII	17-XVI	18-XIV	19-XVII[a]	20-XIX	21-XX	22-XXI	23-XXII	X	
GROUP 1	E.5	3	++		+++	+++	+++	+++	++	++		++							+++			+++	+++			+++	SF1
	E.50	1	+++		++		+++	++		+++	+++	+++							++			+++	+++			++	SF1
	E.59	1		++					++			++											++				SF1
	E.73	6	++		+++	++		+++	+++			++											++				SF1
	F.8	5	+++	++	++	+++	+++	+++	+++	+++	++	+++							+++			+++	+++			+++	SF1
	G.100	6	+++			++	+++					+++										++	+++				SF1
	G.105	6	++		+++		++		+++	++	++												+++			++	SF1
	A1.36	4+3[c]				++			++												+++	++					monomeric
	E.32	3			++							++							+++		+++					++	SF1/monomeric
	E.54	2	+++								+++	+++							+++		++	++	++			++	monomeric
GROUP 2	A1.12	6																		++	+++			++	++		SF2
	A1.50	6											++	++		+++	+++	++		+++	+++				++		SF2
	A1.52	5+5[c]													++						+++						SF2/SF3/monomeric
	A1.60	6											++		++			++		++	+++			++	++		SF2
	A1.64	6											++	++				++		+++	+++			+++	+++		SF2
	E.7	2																			+++				++		SF2
	E.24	2													++			++		++	+++				++		SF2
	E.33	1																++		++	+++				++		SF2
	E.34	1																		++	+++				++		SF2
	E.36	1																			+++						SF2
	E.56	2													++					++	+++			++	++		monomeric
	F.18	5+4[c]											++	++	++	++	++	++		++	+++			++	++		SF2/monomeric
	G.84	4													+++					++	++			+++	++		SF2
	A1.49	6				++							++		++		++				++			++	++		monomeric
	E.12	3				+++									++			++		++	+++			++	++		monomeric
	E.83	5				++							++		+++					++	++			++	++		monomeric
	F.94	4				+++							++		++			++			++			++			monomeric
GROUP 3	A1.19	3+4[c]		++		++							++							++	++						SF3/monomeric
	A1.40	8				++		++			++			++						++	+++				++		SF2/monomeric
	A1.44	5+4[c]	++			+++		++		++					++				+++	++		++	++	++	++	++	monomeric
	A1.73	3				++								++	+++			+++		+++	+++	++		++	+++	++	SF2/monomeric
	C8	1				+++	+++					+++									+++	+++	+++		+++	++	monomeric
	E.27	3																		++	+++				++	++	SF3
	E.35	2									++	+++	++						++			++				++	SF1/monomeric
	E.95	3											++		+++						+++	++			+++		monomeric
	E.104	3				++									++	++	++	++		++	+++	++		++	++	++	monomeric
	G.18	5	++	+++		++	++	++	++	+++	++	++	++	++		++		++	++	++	++	++	+++	++	++	+++	SF1/SF2
	G.81	1		+++		+++	++														++				++		monomeric
	G.97	6												++	+++		++	++			+++	++		++	++		monomeric
GROUP 4	E.1	2																			+						monomeric
	E.31	3																			+						monomeric

Plus represents the intensity of the detected signals: "++" medium and "+++" strong. Chromosome cells are colored based on the Group 1 and 2 hybridization patterns.

[a]Gorilla chromosomes V and XVII contain the centromeres of human chromosomes 17 and 5, respectively.

[b]According to the monomeric distribution in the phylogenetic tree.

[c]All clones were bidirectionally sequenced. In these cases the inserts were too long to be joined into single sequences.

Table S6. Hybridization results of 69 gorilla centromeric clones on human metaphase chromosomes, classified by hybridization pattern.

	PLASMID/BAC	HSA chromosome																							gorilla suprachromosomal family (SF)[b]
		1	2	3	4	5[a]	6	7	8	9	10	11	12	13	14	15	16	17[a]	18	19	20	21	22	X	
	E.5	+++		+++		+++		+++			+++		+++				++			+++					SF1
	E.50	+++				++		++			++	++	++				++			+++					SF1
	E.59			+++				+++			+++		+++				+++								SF1
	E.73	+++		+++		+++							++				++								SF1
	F.8	+++		+++				++					++							+++					SF1
	G.100	+++		++		+++		++					+++				++			+++					SF1
	G.105	+++		+++		++		+++					++				++			+++					SF1
	CH255-54G3	+++		++		+++		++			++		++				+++			+++					NA
	CH255-65K23	+++		++		+++		+++			++		++				++			+++					NA
	CH255-74N2	+++		+++		+++		+++			++		++												NA
	CH255-82B2	+++		++		+++		+++					+++				++			+++					NA
	CH255-90G14	+++		+++		+++		++			++		+++				++			+++					NA
	CH255-54A1	+++		+++		+++		++			++		+++				++			+++					NA
	CH255-63P2																								NA
	CH255-74D1	+++		++		+++		++			++		++				++			++					NA
	CH255-77P5	+++		++		++		++			++		+++				+++			+++					NA
	CH255-72A8	+++		+++		+++		+++			+++		+++				+++			+++					NA
	CH255-83C18	+++		+++		+++		+++					+++				+++								NA
	CH255-54L2	+++		++		++		+++					+++				+++			+++					NA
	CH255-94H12	++		++		++		++			++		++				++								NA
	CH255-69O1	+++		+++		+++		+++					++				++			+++					NA
GROUP 1	CH255-75P5	+++		++		+++		+++			+++		++				++			+++					NA
	CH255-85J11	+++		++		+++		++			++		++				++			++					NA
	CH255-77K24							++									++								NA
	CH255-86E1	+++		++		+++		++			++		++				+++			+++					NA
	CH255-93P10	+++		+++		+++		+++			+++		+++				+++			+++					NA
	CH255-52M24	+++		+++		+++		+++			+++		++				+++			+++					NA
	CH255-53A22	+++				++		+++			++		++				++			++					NA
	CH255-58E7	+++		++		++		+++			++		++				++								NA
	CH255-62A2	+++		+++		+++		+++			+++		++				++			+++					NA
	CH255-68K1	+++		+++		+++		+++			++		+++				+++			+++					NA
	CH255-75M1	+++		++		+++		+++			++		+++				+++			+++					NA
	CH255-74P7	+++				+++		+++			+++						+++			++					NA
	CH255-86F14	+++		++		++		++					++												NA
	CH255-91A3	+++		++		+++		+++			++		++				+++			++					NA
	CH255-91A8	++		++		++		+++			+++		++				++			++					NA
	CH255-73I14	++		++		++		++			++		++												NA
	CH255-77N24	++		++		++		++			++		++				++			++					NA
	CH255-62P2	+++		++		++		+++			++		++				++			+++					NA
	A1.36				++			++							+++	++					+++	++		+++	monomeric
	E.32	+++	+++	+++	++	+++		+++	++	++		++	+++	++	++	++	+++		+++	++	+++	++	++		SF1+monomeric
	E.54	+++		+++		+++		+++	+++	+++	+++	+++	+++				+++		+++	+++	+++			+++	monomeric
	CH255-77L1	+++	+++	+++	++	+++		+++	++	++	++	+++	++				++	++	+++	++	+++				
	A1.12		+++											++	+++				+++		+++	++			SF2
	A1.50		+++							++					+++	++					++				SF2
	A1.60															++									SF2
	A1.64														++				++						SF2
	E.7		+++		+++					+++					+++	+++			+++		+++		+++		SF2
	E.24		+++		+++					+++					+++	+++					+++		+++		SF2
	E.33														+++								+		SF2
	E.34		+++		+++					+++				+++	+++	+++			+++		+++	+++	+++		SF2
	E.36				+++										+++										SF2
	G.84				+++					+++				++	++	++			++		+++	+++	++		SF2
GROUP 2	CH255-90D8		+++		+++				++	+++					++	++			++		+++		+++		NA
	CH255-58L10		+++		+++				++	+++				++	++	+++			+++		++	+++	++		NA
	CH255-67O23		++		+++				++	+++				+++	+++	+++			++		++	+++	+++		NA
	CH255-49C21		+++		+++				++	++				++	+++	++			++		+++	++	+++		NA
	CH255-91G23				++				++	+++				+++	+++	+++			+++		++	+++	+++		NA
	CH255-61G22		+++		+++				++	+++				+++	+++	+++			++		+++	+++	+++		NA
	CH255-73G21																								NA
	CH255-50P4		++		++				++	+++				++	++	++			+++		++	+++	++		NA
	A1.49		++	++	+++							++			++	++			+++	++	+++	++			monomeric
	E.83							+++				++			++			+++		++	++		++		monomeric
	F.94			++	+++	+++		+++		++		++	++							+++	+++			+++	monomeric
	A1.19												++			++			+++		+++	++			SF3+monomeric
	A1.40		++						++							++							++		SF2+monomeric
	A1.44	++	+++		++	++							+++		++			++	++		+++		+++		monomeric
	A1.73											++			++							++			SF2+monomeric
	C8	++		++	++	++	++	++				++	++					++		++			++		SF3+monomeric
GROUP 3	E.27											+++						+++							SF3
	E.35	+++	+++			+++					+++	+++					+++	+++	+++	+++					SF3+monomeric
	E.95	++		++		+++		++		+++		++	++								+++		++	+++	monomeric
	E.104	++		++		++		+++		++		++								+++			+++	++	monomeric
	G.18	++	++	++	++		++				++														SF1+SF2
	G.81	++	++	+++		++	++										++			+++	+++	++			monomeric
	G.97				++	++			++						++				++	+++	+++				monomeric
	A1.15		++		++	++													++	++	++				NA
GROUP 4	E.1							+++		+++										+++	+++				monomeric
	E.31	++	++	++	++	++	++	++	++	+++		++	++	++	++	++		++	+++	++	+++	++	+++		monomeric

Note. Chromosome cell colors reflect gSF: light gray for gSF1; dark gray for gSF2 (according to our data). "Exceptional" clones indicated below the dotted line.

[a]Chromosomes 5 and 17 cells have been colored as gorilla chromosomes XVII and V, respectively, according to their centromere.

[b]According to the monomeric distribution in the phylogenetic tree.

Table S7. Hybridization results of 16 gorilla centromeric clones on chimpanzee and orangutan metaphase chromosomes, classified by hybridization pattern.

	PLASMID/BAC	PTR chromosome																								gorilla suprachromosomal family (SF)b
		1-I	2-III	3-IV	4-V	5-VI	6-VII	7-VIII	8-X	9-XI	10-XII	11-IX	12-IIp	13-IIq	14-XIII	15-XIV	16-XV	17-XVIII	18-XVI	19-XVII	20-XIX	21-XX	22-XXI	23-XXII	X	
SF1	E.5		++				++									+++		++			++	++	++	+++		SF1
	E.73		++				++								++	+++	++	++			++	++	++	+++		SF1
	F.8		++				++								++	+++		++			++	++	++	+++		SF1
	G.105		++				++									+++		++			++	++	++	+++		SF1
	CH255-54G3			++												++	++	++	++		+++	+++	+++	+++		
	CH255-72A8		++						++		++					+++	++	+++	+++		+++	+++	+++	+++		
	CH255-77K24		++	++												++			++		++	++	+++	+++		
	CH255-52M24		++	++					+++				++			+++	++		++		+++	+++		+++		
	CH255-62P2		++	++												+++	++	++	++		+++	++	+++	+++		
	CH255-77L1		++	++				++	++		++					+++	+++	++	+	++	+++	++	++	++	++	
SF2	E.7									++		++		++												SF2
	G.84									++		++		++												SF2
	CH255-49C21											+++		++												
	CH255-73G21											+++		+++												
	CH255-50P4											+++		+++												
SF3	E.27	++				++				+++			++							++					+++	SF3

	PLASMID/BAC	PPY chromosome																								gorilla suprachromosomal family (SF)
		1-I	2-III	3-IV	4-V	5-VI	6-VIII	7-X	8-XI	9-XII	10-VII	11-IIq	12-IIp	13-IX	14-XIII	15-XIV	16-XV	17-XVIII	18-XVI	19-XVII	20-XIX	21-XX	22-XXI	23-XXII	X	
SF1	E.5												NO SIGNAL													SF1
	E.73												NO SIGNAL													SF1
	F.8												NO SIGNAL													SF1
	G.105												NO SIGNAL													SF1
	CH255-54G3												NO SIGNAL													
	CH255-72A8												NO SIGNAL													
	CH255-77K24												NO SIGNAL													
	CH255-52M24												NO SIGNAL													
	CH255-62P2												NO SIGNAL													
	CH255-77L1	++	++	+			+	++								++	+		++	+		+	+	++		
SF2	E.7	++	++	++	++		++	++	++		++	++	++	++	++	++	++	++	++	++	++	++	++	++	++	SF2
	G.84		+++	++	++		++	++	++		++	++	++	++	++	++	++	++	++	++	++	++	++	++	++	SF2
	CH255-49C21		++						++			++		++							++		+++	+++		
	CH255-73G21		++										++			++			++		++		++	+++		
	CH255-50P4		+++	++			++	++				++					++	++	+++	++	++	+++	++	+++		
SF3	E.27												++							++				++	++	SF3

Note. Chromosome cell colors reflect gSF: light gray for gSF1; dark gray for gSF2 (according to our data). "Exceptional" clones indicated below the dotted line.

[b]According to the monomeric distribution in the phylogenetic tree.

Supplementary Note

Organization and evolution of Gorilla centromeric DNA from old strategies to new approaches

Catacchio CR[1], Ragone R[1], Chiatante G[1] and Ventura M[1†]

[1]Department of Biology, University of Bari Aldo Moro, Via Orabona 4, 70125 Bari, Italy

Section 1: Gorilla α-satellite containing plasmids 7

Section 2: Gorilla α-satellite containing BACs 9

Section 3: Gorilla α-satellite containing WGSS and sequence analyses 10

Section 4: Literature Cited 10

Section 1: Gorilla α-satellite containing plasmids

By subcloning the α-27/α-30 GGO α-satellite, over 300 clones were obtained; 71 of them were tested by FISH on gorilla metaphase spreads (Table S1). 68/71 clones were grouped in two main subsets with similar hybridization patterns (Figure 1, Table S1, Supplementary Note Table 1): (A) 7/71 clones with signals on chromosomes I, III-VIII, X-XII, XVI, XIX, XX, and X, or subsets of them (plus four "exceptional" clones hybridizing also on chromosome XVII); (B) 30/71 clones with signals on the acrocentric chromosomes IIq, IIp, IX, XIII-XV, XVII, XVIII, XXI and XXII, or subsets of them (plus 12 "exceptional" clones hybridizing also on chromosome V). In addition, 15/71 clones hybridized on chromosomes belonging to both groups (Group 3) and 3/71 clones hybridized as single pericentromeric probes (Group 4).

52 clones from all four different groups were bidirectionally sequenced and regions of overlap between the two sequences of each clone were successfully joined into a longer sequence in 47/52 cases. 57 sequences were obtained: 47 complete sequences plus 10 ends. Preliminarily to any further analysis, the examination of the sequences by RepeatMasker indicated that they were composed entirely of α-satellite (Smit, Hubley et al. 1996-2010).

Sequences were split into ~171 bp monomers (min 165 bp; max 191 bp). The first base position was based on the starting point of the human alpha satellite consensus sequences (GenBank accession numbers AJ130751.1-AJ130762.1). The alignment of the first monomer of each clone against its whole sequence by BLAST (Altschul, Gish et al. 1990) showed identity percentages ranging from 75–97% without any recurrent periodicity for 34/39 comparisons (87.2%), reflecting the typical monomeric organization of the α-satellite (Table 3). 5/39 clones (12.8%) (E.5, E.73, F.8, G.100,

G.105), instead, exhibited an evident periodicity of ~340 bp with 87-95% identity between corresponding monomers of different HOR units in the same array, and 65-83% between monomers at non-corresponding positions. The same dimeric structure was concordantly confirmed by JDotter, Tandem Repeats Finder (TRF) and *in silico* enzymatic restriction by NebCutter V2.0 (Supplementary Note Table 2).

Regions harboring the protein-recognition domain (PRD) (CENP-B box or pJα motif) were finely studied; when essential (in bold) and nonessential positions were discordant, the PRD was assigned as follows: if |("mismatches if CENP-B box"-" mismatches if pJα motif")|≤2 in the essential positions and |("mismatches if CENP-B box"-" mismatches if pJα motif")|≥3 in the nonessential positions, the PRD was assigned concordantly to nonessential positions. If instead |("mismatches if CENP-B box"-" mismatches if pJα motif")|≤2 in the essential positions but |("mismatches if CENP-B box"-" mismatches if pJα motif")|<3 in the nonessential positions, the monomer was considered "no box". Monomers were also considered "no box" in the case of equality of mismatches (clones A1.50_5, A1.60_4, A1.64_5) (Supplementary Note Table 3). Possible PRD-alternance has also been considered (Supplementary Note Table 3, last column).

Southern blot analyses were performed showing a ladder of hybridizing bands of ~340 bp in EcoRI-digests. Both the 340 bp band from the genomic digestion (Supplementary Note Figure S1) and a mix of plasmids from all three main hybridization groups (G.105, G.84, G.97) were used as probes and gave identical patterns.

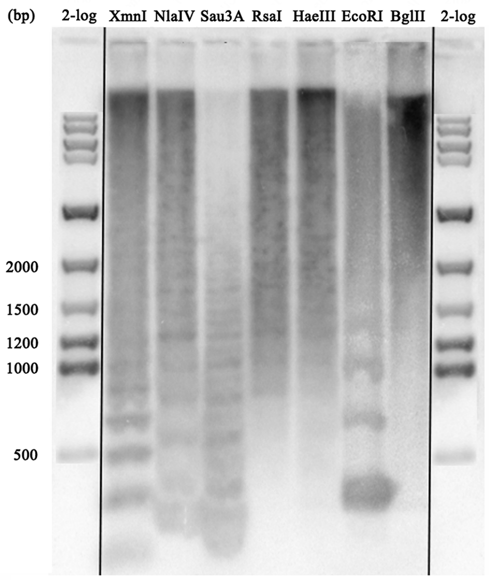

Supplementary Note Figure S1 Southern blot experiment on gorilla genomic DNA. The probe used was the smallest fragment obtained by digesting GGO genomic DNA with EcoRI – corresponding to the ~340 bp band. There were two kinds of patterns: a ~170 bp ladder in the lane of the DNA digested with XmnI and a ~340 bp ladder in the lane of the DNA digested with EcoRI (both visible in other lanes too, but less intensely). A mix of plasmids covering together all chromosome centromeres (G.105, G.84, G.97) gave identical patterns. Enzymes used for the digestion are reported on the top line of the picture.

Section 2: Gorilla α-satellite containing BACs

We screened a gorilla BAC library using as probes a mix of plasmids from the three centromeric hybridization groups (G.105, G.84, G.97). We selected 41 α-satellite-containing BAC clones showing stronger hybridization signals and tested them by both FISH and enzymatic restriction. FISH results on GGO metaphase spreads showed the same two main patterns identified by hybridizations with plasmid clones: 32/41 BACs mapped as Group 1 plasmids (plus one "exceptional" clone) and 8/41 BACs as Group 2 (Table 1).

Section 3: Gorilla α-satellite containing WGSS and sequence analyses

We picked out, by BLASTn, 66 highly scored WGSS (whole-genome shotgun sequence assembly) sequences, using the α-satellite containing plasmidic clones as the query. 2351 monomers were derived from these sequences and aligned together with monomers extracted from plasmids.

The multi-alignment of the SF-specific group of monomers displayed SF-specific diagnostic mutations and different degrees of PRD conservation among monomer types (Supplementary Note Tables 4-6).

p-distance matrices between the 2521 gorilla alphoid monomers and both the 12 human alphoid and 16 gorilla alphoid consensus sequences were created; monomer-type correspondence based on divergence for each monomer was then assessed (Supplementary Note Tables 7-9).

Section 4: Literature Cited

Altschul, S. F., W. Gish, et al. (1990). Basic local alignment search tool. Journal of molecular biology 215(3): 403-410.

Smit, A., R. Hubley, et al. (1996-2010). RepeatMasker Open-3.0. <http://www.repeatmasker.org>.

Supplementary Note Table 1. Summary of the fluorescently hybridized gorilla centromeric plasmids on gorilla metaphase chromosomes. "Exceptional" clones indicated in brackets.

Group	Mapping	Clone count
1	I, III-VIII, X-XII, XVI, XIX, XX, X	7/71 (+4/71)
2	IIq, IIp, IX, XIII-XV, XVII, XVIII, XXI, XXII	30/71 (+12/71)
3	All chromosomes	15/71
4	Single pericentromeric signal	3/71

Supplementary Note Table 2. Sequence analyses applied on five dimeric alphoid plasmid clones: Tandem Repeats Finder and NebCutter results. All detected a ~340 bp periodicity.

		**Tandem Repeats Finder**							**NEB Cutter**	
	size (bp)	Indices	Period Size	Copy Number	Consensus Size	Percent Matches	Percent Indels	Score	RE	fragment size (bp)
**E.5**	678	5--673	340	2.0	340	93	0	1140	HaeIII	340
		140--678	169	3.2	169	73	3	551		
**F.8**	1018	5--1013	340	3.0	340	93	1	1698	EcoRI	340
**E.73**	1196	1--1181	340	3.5	340	92	1	1956	EcoRI	340
**G.100**	1189	4--1189	340	3.5	339	85	2	1549	EcoRI, HinfI	340
		5--1189	171	7.0	170	69	8	699		
		168--1057	169	5.2	169	70	7	638		
**G.105**	1185	1--1180	340	3.5	340	93	0	1963	EcoRI, HinfI	340

Supplementary Note Table 3. Protein recognition domain (PRD) analysis and assignment of the 220 17-bp sequences extracted from gorilla plasmid inserts.

monomer ID	monomer lenght	monomer number	row box alignment	substitution matrix with pJα motif	substitution matrix with CENP-B box	essential positions: pJα motif substituted letters	essential positions: CENP-B box substituted letters	essential positions: mismatches if pJα motif	essential positions: mismatches if CENP-B box	essential positions: monomer type	non-essential positions: pJα motif substituted letters	non-essential positions: CENP-B box substituted letters	non-essential positions: mismatches if pJα motif	non-essential positions: mismatches if CENP-B box	non-essential positions: monomer type	assigned PRD	PRD alternance
GGO_consensus	172		C T T C G T/G T G G A A A C/A - - - - G G A/G A	C T A C/T G G T G G/A A A A A - - - - G G A A	T/C T T C G T T G G A A A/G C - - - - G G G A												
A.26*			. A . . . . . A . . . . . - - - -	. A T . . . . A . . . . . - - - -	. A . . . G . A . . . . A - - - - . . A .	A	AAA	1	3	α	TA	AG				α	
A.30*			. . A T . . . . A . . . . - - - -	. . . . - - - -	. . A T . G . . . . . . A - - - - . . A .		ATAA	0	4	α		G				α	
A.35*			. . A T . . . . - - - - T . . .	. . . . - - - - T . . .	. . A T . G . . . . . . A - - - - T . A .	T	ATATA	1	5	α		G				α	
A1.12_1	172		. . A A . A . . . . - - - -	. . . A . A . . . . - - - -	. . A A . A . . . . . . A - - - - . . A .		AAAA	0	4	α	AA	A				α	
A1.12_2	172		T . C . . . G . . . . . . - - - - C . . .	T . C . . T G . . . . . C - - - - C . G .	. . C . . . G . . . . . . - - - - C . . .	CC	CC	2	2	NA	TCTGG	G	5	1	B	B	
A1.12_3	172		. . A T . C . . A G . . . - - - - C . . .	. . . . . C . . . G . . . - - - - C . G .	. . A T . C . . . G . . A - - - - C . . .	GC	ATGAC	2	5	α	CG	C				α	
A1.12_4	167		T . . . . - - - - - - - -	T . T . . T . . . . . . C - - - - - - - -	. . . . - - - - - - - -	C--	---	3	3	NA	TTT	-	3	1	B	B	
A1.12_5	189	6	T . . . . C T T T . A . G	T . T C . T . . . . . . C C T T T . A G G	. . . . C T T T . A . G	CCTTTA	CTTTA	6	5	NA	TTCTGG	G	6	1	B	B	NO
				T . T C . . . . C - - - - . . G .	. . . . - - - -	C		1	0	NA	TTCG		4	0	B	B	
A1.12_6	171		. . A T . . . . A G . . G - - - -	. . . . . T . . . G . . G - - - -	. . A T . . . . . G . . G - - - - . . A .	GG	ATGGA	2	5	α	T					α	
A1.12_*			A . . . . - - - -	A . T . . T . . . . . . C - - - - . . G .	A . . . . - - - -	C		1	0	NA	ATTG		4	0	B	B	
A1.19F_1	171		. . A T A . . T . . . . . - - - -	. . . . A . . T . . . . . - - - -	. . A T A G . T . . . . A - - - - . . A .	T	ATAAA	1	5	α	A	GT				α	
A1.19F_2	171		. . . . C . . . . . - - - - . . . T	. . T . . T . C . . . . C - - - - . . G T	. . . . C . . . . . - - - - . . . T	CC		2	0	B	TTGT	CT				B	
A1.19F_3	171	3	T . . . T . . . . - - - -	T . T . T T . . . . . . C - - - - . . G .	. . . . T . . . . - - - -	C	T	1	1	NA	TTTTG		5	0	B	B	NO
A1.19F_*			. . G T . . . . - - - -	. . G . . . . - - - -	. . G T . G . . . . . . A - - - - . . A .		GTAA	0	4	α	G	G				α	
A1.19R_1	171		. . A T . . . A . . . . . - - - -	. . . . A . . . . . - - - -	. . A T . G . A . . . . A - - - - . . A .	A	ATAA	1	4	α		GA				α	
A1.19R_2	171		. C . . . . T - - - -	. C T . . T . . . . . . T - - - - . . G .	. C . . . . T - - - -	T	CT	1	2	NA	CTTG		4	0	B	B	
A1.19R_3	170	3	. . G T . - . . T . . . . - - - -	. . G . . - . . T . . . . - - - -	. . G T . - . . T . . . A - - - - . . A .	T	GTAA	1	4	α	G-	T-				α	YES
A1.19R_4	169		. . . . A C . . T T . . T - - - -	. . T . A C . . T T . . T - - - - . . G .	. . . . A C . . T T . . T - - - -	TTT	ATT	3	3	NA	TACG	CT	4	2	B	B	
A1.36F_1	171		T . . . . - - - -	T . T . . T . . . . . . C - - - - . . G .	. . . . - - - -	C		1	0	NA	TTTG		4	0	B	B	
A1.36F_2	171		. . A T . A A . . . . . . - - - -	. . . . . A A . . . . . . - - - -	. . A T . A A . . . . . A - - - - . . A .		ATAA	0	4	α	AA	AA				α	
A1.36F_3	170	4	. . A . . A . . . . - - - -	. . . . . A . . . . - - - -	. . A . . A . . . . . . A - - - - . . A .		AAA	0	3	α	A	A				α	NO
A1.36F_4	171		. . A T . . . . T . . . . - - - - A . . .	. . . . T . . . . - - - - A . . .	. . A T . G . . T . . . A - - - - A . A .	TA	ATAAA	2	5	α		GT				α	
A1.36F_*			. . A T . . . A . . . . . - - - -	. . . . A . . . . . - - - -	. . A T . G . A . . . . A - - - - . . A .	A	ATAA	1	4	α		GA				α	
A1.36R_1	171		. . G T . . . . - - - - . . . G	. . G . . . . - - - - . . G G	. . G T . G . . . . . . A - - - - . . . G		GTA	0	3	α	GGG	GG				α	
A1.36R_2	170		. . . . A . A . . . . . . - - - - A . T G	. . T . A T A . . . . . C - - - - A . T G	. . . . A . A . . . . . . - - - - A . T G	CA	AAT	2	3	NA	TATATG	AG	6	2	B	B	
A1.36R_3	169	4	. . . . A . . . . - - - - C . . .	. . T . A T . . . . . . C - - - - C . G .	. . . . A . . . . - - - - C . . .	CC	AC	2	2	NA	TATG		4	0	B	B	NO
A1.36R_*			. . G T A . . C C . . . . - - - -	. . G . A . . C C . . . . - - - -	. . G T A G . C C . . . A - - - - . . A .	CC	GTAAA	2	5	α	GA	GCC				α	
A1.40_1	171		. . G T . . . . - - - - . . . T	. . G . . . . - - - - . . . T	. . G T . G . . . . . . A - - - - . . A T		GTAA	0	4	α	GT	GT				α	
A1.40_2	171		. . A . A . . A . . . . . - - - -	. . . . A . . A . . . . . - - - -	. . A . A G . A . . . . A - - - - . . A .	A	AAAA	1	4	α	A	AG				α	
A1.40_3	170		. . . . T . . . . . - - - -	. . T . . T . T . . . . C - - - - . . G .	. . . . T . . . . . - - - -	TC		2	0	B	TTG	T				B	
A1.40_4	171		. . A T . . A . A . . . . - - - -	. . . . . . A . . . . . . - - - -	. . A T . G A . . . . . A - - - - . . A .		ATAA	0	4	α	A	GA				α	
A1.40_5	171	8	. . A T A . . . A T . . . - - - - . . T .	. . . . A . . . . T . . . - - - - . . T .	. . A T A G . . . T . . A - - - - . . T .	T	ATATAT	1	6	α	AT	G				α	NO
A1.40_6	171		. A A T . . A . A . . . . - - - - C C . .	. A . . . . A . . . . . . - - - - C C . .	. A A T . G A . . . . . A - - - - C C A .	CC	AATACCA	2	7	α	AA	GA				α	
A1.40_7	171		. . A T . C . . A G . . G - - - -	. . . . . C . . . G . . G - - - -	. . A T . C . . . G . . G - - - - . . A .	GG	ATGGA	2	5	α	C	C				α	
A1.40_8	171		A . . . . - - - -	A . T . . T . . . . . . C - - - - . . G .	A . . . . - - - -	C		1	0	NA	ATTG	A	4	1	B	B	
A1.40_*			. . A T . . . . A . . . . - - - -	. . . . - - - -	. . A T . G . . . . . . A - - - - . . A .		ATAA	0	4	α		G				α	
A1.44F_1	169		. . . . T . . . . - - - -	. . T . T T . . . . . . C - - - - . . G .	. . . . T . . . . - - - -	C	T	1	1	NA	TTTG		4	0	B	B	
A1.44F_2	171		. . . T . . . . A . . . . - - - -	. . T . . . . - - - -	. . . T . G . . . . . . A - - - - . . A .		TAA	0	3	α	T	G				α	
A1.44F_3	171	5	T A . . T . . . . - - - -	T A T . T T . . . . . . C - - - - . . G .	. A . . T . . . . - - - -	C	AT	1	2	NA	TATTTG		6	0	B	B	YES
A1.44F_4	171		. . A T . . . . - - - -	. . . . - - - -	. . A T . G . . . . . . A - - - - . . A .		ATAA	0	4	α		G				α	
A1.44F_5			T . . . . - - - -	T . T . . T . . . . . . C - - - - . . G .	. . . . - - - -	C		1	0	NA	TTTG		4	0	B	B	
A1.44R_1	163		. . G T . - . . T . . . . - - - -	. . G . . - . . T . . . . - - - -	. . G T . - . . T . . . A - - - - . . A .	T	GTAA	1	4	α	G-	T-				α	
A1.44R_2	171		. . . . . A . . . . - - - -	. . T . . A . . . . - - - -	. . . . . A . . . . . . A - - - - . . A .		AA	0	2	α	TA	A				α	
A1.44R_3	171	4	. . A . . A . . . . - - - -	. . . . . A . . . . - - - -	. . A . . A . . . . . . A - - - - . . A .		AAA	0	3	α	A	A				α	NO
A1.44R_4	171		. C A T . A . T . . . . . - - - -	. C . . . A . T . . . . . - - - -	. C A T . A . T . . . . A - - - - . . A .	T	CATAA	1	5	α	CA	AT				α	
A1.44R_*			. . A T . . . . - - - -	. . . . - - - -	. . A T . G . . . . . . A - - - - . . A .		ATAA	0	4	α		G				α	
A1.49_1	171		. . A T . A . . . . - - - -	. . . . . A . . . . - - - -	. . A T . A . . . . . . A - - - - . . A .		ATAA	0	4	α	A	A				α	
A1.49_2	171		. . . . A C . . T T . . T - - - -	. . T . A C . . T T . . T - - - - . . G .	. . . . A C . . T T . . T - - - -	TTT	ATT	3	3	NA	TACG	CT	4	2	B	B	
A1.49_3	171		. . A . . . . - - - -	. . . . - - - -	. . A . . G . . . . . . A - - - - . . A .		AAA	0	3	α		G				α	
A1.49_4	166	6	. . . T . C . . . . . . T - - - -	. . T . . C . . . . . . T - - - - . . G .	. . . T . C . . . . . . T - - - -	T	TT	1	2	NA	CTG	C	3	1	no box	no box	NO
A1.49_5	171		. . . T . . . . - - - -	. . T . . . . - - - -	. . . T . G . . . . . . A - - - - . . A .		TAA	0	3	α	T	G				α	
A1.49_6	188		. . A . . . . - - - -	. . . . - - - -	. . A . . G . . . . . . A - - - - . . A .		AAA	0	3	α		G				α	
A1.50_1	171		. . A T . A . . . . - - - -	. . . . . A . . . . - - - -	. . A T . A . . . . . . A - - - - . . A .		ATAA	0	4	α	A	A				α	
A1.50_2	171		T . . . . - - - -	T . T . . T . . . . . . C - - - - . . G .	. . . . - - - -	C		1	0	NA	TTTG		4	0	B	B	
A1.50_3	171		. . A T . C . . A G . . . - - - - C . . .	. . . . . C . . . G . . . - - - - C . G .	. . A T . C . . . G . . A - - - - C . . .	GC	ATGAC	2	5	α	CG	C				α	
A1.50_4	171	6	T . C . . . . - - - -	T . C . . T . . . . . . C - - - - . . G .	. . C . . . . - - - -	C	C	1	1	NA	TTCG		4	0	B	B	NO
A1.50_5	167		T . . . . - - - - - - - -	T . T . . T . . . . . . C - - - - - - - -	. . . . - - - - - - - -	C--	---	3	3	NA	TTTC	-	1	1	no box	no box	
A1.50_6	186		T . . . . C T T T . A . G	T . T C . T . . . . . . C C T T T . A G G	. . . . C T T T . A . G	CCTTTA	CTTTA	6	5	NA	TTCTGG	G	6	1	B	B	
				T . T C . . . . C - - - - . . G .	. . . . - - - -	C		1	0	NA	TTCG		4	0	B	B	

A1.52F_1	171		T . . . . - - - -	T . T . . T . . . . . C - - - - - - . G .	. . . . - - - - - - . . .	C		1	0	NA	TTTG		4	0	B	B	
A1.52F_2	171		. . A T . A A . . . . . - - - -	. . . . . A A . . . . . - - - - - - . . .	. . A T . A A . . . . A - - - - - - . A .		ATAA	0	4	α	AA	AA				α	
A1.52F_3	170	5	. . A . . A . . . . . . - - - -	. . . . . A . . . . . . - - - - - - . . .	. . A . . A . . . . A - - - - - - . A .		AAA	0	3	α	A	A				α	NO
A1.52F_4	171		. . A T . . . T . . . . - - - - A . . .	. . . . T . . . . - - - - - - A . . .	. . A T . G . T . . A - - - - A . A .	TA	ATAAA	2	5	α		GT				α	
A1.52F_5	171		. . A T . . . A . . . . - - - -	. . . . . . A . . . . . - - - - - - . . .	. . A T . G . A . . A - - - - - - . A .	A	ATAA	1	4	α		GA				α	
A1.52R_1	169		. A A T . . . A . . . . - - - -	. A . . . . A . . . . . - - - - - - . A A	. A A T . G . A . . A - - - - - - . A .	A	AATAA	1	5	α	AAA	GA	3	2	NA	α	
A1.52R_2	171		. . G T . . . . - - - - . . . G	. . G . . . . - - - - - - . G G	. . G T . G . . . . A - - - - - - . . G		GTA	0	3	α	GGG	GG				α	
A1.52R_3	170	5	. . . . A . A . . . . . - - - - A . T G	. . T . A T A . . . . C - - - - A . T G	. . . . A . A . . . . - - - - - A . T G	CA	AAT	2	3	NA	TATATG	AG	6	2	B	B	NO
A1.52R_4	169		. . . . A . . . . - - - - C . . .	. . T . A T . . . . . C - - - - C . G .	. . . . A . . . . . . - - - - - C . . .	CC	AC	2	2	NA	TATG		4	0	B	B	
A1.52R_5	171		. . G T A . . C C . . . - - - -	. . G . A . C C . . . . - - - - - - . . .	. . G T A G . C C . . A - - - - - - . A .	CC	GTAAA	2	5	α	GA	GCC				α	
A1.60_1	171		. . A A . A . . . . . . - - - -	. . . A . A . . . . . . - - - - - - . . .	. . A A . A . . . . A - - - - - - . A .		AAAA	0	4	α	AA	A				α	
A1.60_2	170		T . . . . - - - -	T . T . . T . . . . C - - - - - - . G .	. . . . - - - - - - . . .	C		1	0	NA	TTTG		4	0	B	B	
A1.60_3	171		. . A T . C . . A G . . . - - - - C . . .	. . . . . C . . G . . . - - - - - C . G .	. . A T . C . . G . A - - - - C . . .	GC	ATGAC	2	5	α	CG	C				α	
A1.60_4	167		T . . . . - - - - - - - - -	T . T . . T . . . . C - - - - - - - - -	. . . . - - - - - - - - -	C--	---	3	3	NA	TTTC	-	1	1	no box	no box	
A1.60_5	189	6	T . . . . C TTT . A . G	T . T C . T . . . . C C T T T . A G G	. . . . C T T T . A . G	CCTTTA	CTTTA	6	5	NA	TTCTGG	G	6	1	B	B	NO
				T . T C . . . . . . C - - - - - - . G .	. . . . - - - - - - . . .	C		1	0	NA	TTCG		4	0	B	B	
A1.60_6	171		. . A T . . . A G . . G - - - -	. . . . . T . . G . . G - - - - - - . . .	. . A T . . . . G . G - - - - - - . A .	GG	ATGGA	2	5	α	T					α	
A1.60_*			A . . . . - - - -	A . T . . T . . . . C - - - - - - . G .	A . . . . - - - - - - . . .	C		1	0	NA	ATTG	A	4	1	B	B	
A1.64_1	169		. . A T . A . . . . . . - - - -	. . . . . A . . . . . . - - - - - - . . .	. . A T . A . . . . A - - - - - - . A .		ATAA	0	4	α	A	A				α	
A1.64_2	171		T . . . . - - - -	T . T . . T . . . . C - - - - - - . G .	. . . . - - - - - - . . .	C		1	0	NA	TTTG		4	0	B	B	
A1.64_3	171		. . A T . C . . A G . . . - - - - C . . .	. . . . . C . . G . . . - - - - - C . G .	. . A T . C . . G . A - - - - C . . .	GC	ATGAC	2	5	α	CG	C				α	
A1.64_4	171	6	T . C . . . . - - - -	T . C . . T . . . . C - - - - - - . G .	. C . . . . - - - - - - . . .	C	C	1	1	NA	TTCG		4	0	B	B	NO
A1.64_5	167		T . . . . - - - - - - - - -	T . T . . T . . . . C - - - - - - - - -	. . . . - - - - - - - - -	C--	---	3	3	NA	TTTC	-	1	1	no box	no box	
A1.64_6	194		T . . . . C TTT . A . G	T . T C . T . . . . C C T T T . A G G	. . . . C T T T . A . G	CCTTTA	CTTTA	6	5	NA	TTCTGG	G	6	1	B	B	
				T . T C . . . . . . C - - - - - - . G .	. . . . - - - - - - . . .	C		1	0	NA	TTCG		4	0	B	B	
A1.73_1	171		. . . . T . . . . - - - -	. . T . . T T . . . C - - - - - - . G .	. . . . T . . . - - - - - - . . .	TC		2	0	B	TTG	T				B	
A1.73_2	171	3	. . A T . . . A . . . . - - - -	. . . . . . A . . . . . - - - - - - . . .	. . A T . G . A . . A - - - - - - . A .	A	ATAA	1	4	α		GA				α	YES
A1.73_3	171		T . . . . - - - -	T . T . . T . . . . C - - - - - - . G .	. . . . - - - - - - . . .	C		1	0	NA	TTTG		4	0	B	B	
A1.73_*			. . A T . C . . A G . . . - - - - C . . .	. . . . . C . . G . . . - - - - - C . G .	. . A T . C . . G . A - - - - C . . .	GC	ATGAC	2	5	α	CG	C				α	
B.7			. . A . . . . - - - -	. . . . - - - - - - . . .	. . A . . G . . . . A - - - - - - . A .		AAA	0	3	α		G				α	
B.42			. . . . C . . . . T - - - - . . . T	. . T . . T . C . . . T - - - - - - . G T	. . . . C . . . T - - - - - - . T	CT	T	2	1	NA	TTGT	CT	4	2	B	B	
C.5			. . A T . . . . . G . . - - - -	. . . . G . . . - - - - - - . . .	. . A T . G . . . G . A - - - - - - . A .	G	ATAA	1	4	α		GG				α	
C8_1	169	1	. . A T . . . . - - - - . . C .	. . . . - - - - - - . C .	. . A T . G . . . . A - - - - - - . C .		ATAC	0	4	α	C	G				α	YES
C8_*			T . . . A . . . . - - - - . . . G	T . T . A T . . . . C - - - - - - . G G	. . . . A . . . . . . - - - - - - . . G	C	A	1	1	NA	TTATGG	G	6	1	B	B	
C13			. . A T . . . A . . . . - - - -	. . . . . . A . . . . . - - - - - - . . .	. . A T . G . A . . A - - - - - - . A .	A	ATAA	1	4	α		GA				α	
C15			. . A T . . . A . . . . - - - -	. . . . . . A . . . . . - - - - - - . . .	. . A T . G . A . . A - - - - - - . A .	A	ATAA	1	4	α		GA				α	
C40			. . G A . . . . A . . . - - - -	. . G A . . . . - - - - - - . . .	. . G A . G . . . . A - - - - - - . A .		GAAA	0	4	α	GA	G				α	
C41			T . . . . - - - -	T . T . . T . . . . C - - - - - - . G .	. . . . - - - - - - . . .	C		1	0	NA	TTTG		4	0	B	B	
E.1_1	172		T . A T . . . . - - - - T . . .	T . . . . - - - - - - T . . .	. . A T . G . . . . A - - - - T . A .	T	ATATA	1	5	α	T	G				α	
E.1_2	171	2	. . . . . C . . . . . . - - - -	. . T . . C . . . . C - - - - - - . G .	. . . . . C . . . . . - - - - - - . . .	C		1	0	NA	TCG	C	3	1	B	B	NO
E.1_*			. . . . T - - - -	. . T . . T . . . . T - - - - - - . G .	. . . . T - - - - - - . . .	T	T	1	1	NA	TTG		3	0	B	B	
E.5_1	171		. A A . A . . A . . . . - - - -	. A . . A . A . . . . . - - - - - - . . .	. A A . A G . A . . A - - - - - - . A .	A	AAAAA	1	5	α	AA	GA				α	
E.5_2	169	3	T . . . . - - - - A . . .	T . T . . T . . . . C - - - - A . G .	. . . . - - - - - A . . .	CA	A	2	1	NA	TTTG		4	0	B	B	YES
E.5_3	171		. A A . . . A . . . . . - - - -	. A . . . . A . . . . . - - - - - - . . .	. A A . . G . A . . A - - - - - - . A .	A	AAAA	1	4	α	A	GA				α	
E.5_*			T G . . . . - - - -	T G T . . T . . . . C - - - - - - . G .	. G . . . . - - - - - - . . .	C	G	1	1	NA	TGTTG		5	0	B	B	
E.7_1	171		T . . . . - - - -	T . T . . T . . . . C - - - - - - . G .	. . . . - - - - - - . . .	C		1	0	NA	TTTG		4	0	B	B	
E.7_2	171	2	. . A T . C . . A G . . . - - - - C . . .	. . . . . C . . G . . . - - - - - C . G .	. . A T . C . . G . A - - - - C . . .	GC	ATGAC	2	5	α	CG	C				α	YES
E.7_*			T . . . . - - - -	T . T . . T . . . . . . - - - - - - . G .	. . . . A - - - - - - . . .		A	0	1	NA	TTTG		4	0	B	B	
E.12_1	171		. . . . T . . . . - - - -	. . T . . T T . . . C - - - - - - . G .	. . . . T . . . - - - - - - . . .	TC		2	0	B	TTG	T				B	
E.12_2	171	3	. . A T . . . A . . . . - - - -	. . . . . . A . . . . . - - - - - - . . .	. . A T . G . A . . A - - - - - - . A .	A	ATAA	1	4	α		GA				α	YES
E.12_3	171		. C . . . . T - - - -	. C T . . T . . . . T - - - - - - . G .	. C . . . . T - - - - - - . . .	T	CT	1	2	NA	CTTG		4	0	B	B	
E.12_*			. . G T . - . . T . . . - - - -	. . G . . - . T . . . . - - - - - - . . .	. . G T . - . . T . . A - - - - - - . A .	T	GTAA	1	4	α	G-	T-				α	
E.20			. . A T . . . T . . . . - - - -	. . . . T . . . . - - - - - - . . .	. . A T . G . T . . A - - - - - - . A .	T	ATAA	1	4	α		GT				α	
E.24_1	171		T . . . . - - - -	T . T . . T . . . . C - - - - - - . G .	. . . . - - - - - - . . .	C		1	0	NA	TTTG		4	0	B	B	
E.24_2	171	2	. . A T . C . . A G . . . - - - - C . . .	. . . . . C . . G . . . - - - - - C . G .	. . A T . C . . G . A - - - - C . . .	GC	ATGAC	2	5	α	CG	C				α	YES
E.24_*			T . . . . - - - -	T . T . . T . . . . C - - - - - - . G .	. . . . - - - - - - . . .	C		1	0	NA	TTTG		4	0	B	B	
E.27_1	171		. . . . C . . . . - - - - C . T T	. . T . . T . C . . . C - - - - C . T T	. . . . C . . . - - - - - C . T T	CCC	CT	3	2	NA	TTTT	CT	4	2	B	B	
E.27_2	171	3	T . . . T . A . . . . . - - - -	T . T . T T A . . . . C - - - - - - . G .	. . . T . A . . . . . - - - - - - . . .	C	T	1	1	NA	TTTTAG	A	6	1	B	B	NO
E.27_3	171		. . A A A . . A . T . . - - - - A . . .	. . . A . A . A . T . . - - - - - - A . . .	. . A A A G . A . T . A - - - - A . A .	ATA	AAATAAA	3	7	α	AA	GA				α	
E.27_*			A . . A T . . . . . . - - - - -	A . T A . T T . . . . . - - - - - - . G .	A . . A T . . . . . . - - - - - - . . .	-	AT-	1	3	α	ATATTG	A				α	
E.31_1	171		. . A . . . . - - - -	. . . . - - - - - - . . .	. . A . . G . . . . A - - - - - - . A .		AAA	0	3	α		G				α	
E.31_2	171	3	. . . T . C . . . . . T - - - -	. . T . . C . . . . T - - - - - - . G .	. . T . C . . . . . T - - - - - - . . .	T	TT	1	2	NA	CTG	C	3	1	no box	no box	NO
E.31_3	171		. . A T . A C . . . . . - - - -	. . . . . A C . . . . . - - - - - - . . .	. . A T . A C . . . A - - - - - - . A .		ATAA	0	4	α	AC	AC				α	
E.31_*			. . A T . A . . . . . . - - - -	. . . . . A . . . . . . - - - - - - . . .	. . A T . A . . . . A - - - - - - . A .		ATAA	0	4	α	A	A				α	
E.32_1	171		. A A A . . . A . . . . - - - -	. A . A . . A . . . . . - - - - - - . . .	. A A A . G . A . . A - - - - - - . A .	A	AAAAA	1	5	α	AA	GA				α	
E.32_2	169	3	. . C T . . . . - - - - C . . .	. . C . . . . - - - - - - C . . .	. . C T . G . . . . A - - - - C . A .	C	CTACA	1	5	α	C	G				α	NO
E.32_3	171		. . A T . . . . - - - -	. . . . - - - - - - . . .	. . A T . G . . . . A - - - - - - . A .		ATAA	0	4	α		G				α	
E.32_*			. . G T . . . . - - - - C . . .	. . G . . T . . . . . . - - - - - - C . . .	. . G T . . . . . . A - - - - C . A .	C	GTACA	1	5	α	GT					α	

E.33_1	171	1	. . A T . . . . A . . . - - - -	. . . . - - - - - - . . .	. . **A T .** G . . . . . **A** - - - - - . **A** .		ATAA	0	4	α		G				α	YES
E.33_*			T . . T . . . . - - - -	T . T . . T . . . . . **C** - - - - - . G .	. . . **T .** . . . . . . - - - - -	C	T	1	1	NA	TTTG		4	0	B	B	
E.34_1	171	1	T . . . . - - - -	T . T . . T . . . . . **C** - - - - - . G .	. . . . - - - - -	C		1	0	NA	TTTG		4	0	B	B	YES
E.34_*			. . A T . C C . A G . . . - - - - C . . .	. . . . C C . . **G** . . - - - - - **C** . G .	. . **A T .** C C . . **G** . . **A** - - - - **C** . . .	GC	ATGAC	2	5	α	CCG	CC				α	
E.35_1	171		. A . . . . A . . . . . - - - -	. A T . . . **A** . . . . - - - - - . . .	. **A** . . G . A . . . . **A** - - - - - . **A** .	A	AAA	1	3	α	TA	AG				α	
E.35_2	169	2	T . . . . - - - -	T . T . . T . . . . . **C** - - - - - . G .	. . . . - - - - -	C		1	0	NA	TTTG		4	0	B	B	YES
E.35_*			. . G T . . . . - - - - . . T .	. . G . . . . - - - - - . T .	. **G T .** G . . . . . **A** - - - - - . **T** .		GTAT	0	4	α	GT	G				α	
E.36_1	171	1	T . . . . - - - -	T . T . . T . . . . . **C** - - - - - . G .	. . . . - - - - -	C		1	0	NA	TTTG		4	0	B	B	YES
E.36_*			. A A T . C . . A G . . . - - - - C . . .	. A . . C . . **G** . . - - - - - **C** . G .	**A A T .** C . . **G** . . **A** - - - - **C** . . .	GC	AATGAC	2	6	α	ACG	C				α	
E.50_1	170	1	. A . . . . A . . . . . - - - -	. A T . . . **A** . . . . - - - - - . . .	. **A** . . G . A . . . . **A** - - - - - . **A** .	A	AAA	1	3	α	TA	AG				α	YES
E.50_*			T . . . . - - - -	T . T . . T . . . . . **C** - - - - - . G .	. . . . - - - - -	C		1	0	NA	TTTG		4	0	B	B	
E.54_1	171		. A A T . . . . - - - -	. A . . . . - - - - - . . .	. **A A T .** G . . . . . **A** - - - - - . **A** .		AATAA	0	5	α	A	G				α	
E.54_2	171	2	. . A T A . . . A . . . - - - -	. . . . A . . . . . . - - - - - . . .	. . **A T A** G . . . . . **A** - - - - - . **A** .		ATAAA	0	5	α	A	G				α	NO
E.54_*			. . . T . C . . . . . . T - - - -	. . T . . C . . . . . **T** - - - - - . G .	. . . **T .** C . . . . . **T** - - - - -	T	TT	1	2	NA	CTG	C	3	1	no box	no box	
E.56_1	169		. . C T . . . . - - - - C . . .	. . C . . . . - - - - - **C** . . .	. **C T .** G . . . . . **A** - - - - **C** . **A** .	C	CTACA	1	5	α	C	G				α	
E.56_2	171	2	. . A T . . . . - - - -	. . . . - - - - - . . .	. **A T .** G . . . . . **A** - - - - - . **A** .		ATAA	0	4	α		G				α	NO
E.56_*			. . A T C A . . . . . . - - - -	. . . . C A . . . . . - - - - - . . .	. **A T C** A . . . . . **A** - - - - - . **A** .		ATCAA	0	5	α	CA	A				α	
E.59_1	171	1	. A . . . . A . . . . . - - - -	. A T . . . **A** . . . . - - - - - . . .	. **A** . . G . A . . . . **A** - - - - - . **A** .	A	AAA	1	3	α	TA	AG				α	YES
E.59_*			T . . . . G . - - - -	T . T . . T . . . . **G C** - - - - - . G .	. . . . - - - - -	GC		2	0	B	TTTG					B	
E.73_1	169		T . C . . . . - - - -	T . C . . T . . . . . **C** - - - - - . G .	. **C** . . . . - - - - -	C	C	1	1	NA	TTCG		4	0	B	B	
E.73_2	171		. A A . . . A . . . . . - - - -	. A . . . . **A** . . . . - - - - - . . .	. **A A .** . G . A . . . **A** - - - - - . **A** .	A	AAAA	1	4	α	A	AG				α	
E.73_3	169		T . C . . . . - - - -	T . C . . T . . . . . **C** - - - - - . G .	. **C** . . . . - - - - -	C	C	1	1	NA	TTCG		4	0	B	B	
E.73_4	172	6	. A A . . . A . . . . . - - - -	. A . . . . **A** . . . . - - - - - . . .	. **A A .** . G . A . . . **A** - - - - - . **A** .	A	AAAA	1	4	α	A	GA				α	YES
E.73_5	169		T . . . . - - - -	T . T . . T . . . . . **C** - - - - - . G .	. . . . - - - - -	C		1	0	NA	TTTG		4	0	B	B	
E.73_6	168		. A A . . A A . . . . . - - - -	. A . . . A **A** . . . . - - - - - . . .	. **A A .** . G A A . . . **A** - - - - - . **A** .	A	AAAA	1	4	α	AA	AAG				α	
E.73_*			T . . . . T - - - -	T . T . . T . . . . . **T** - - - - - . G .	. . . . **T** - - - - -	T	T	1	1	NA	TTTG		4	0	B	B	
E.83_1	171		. . . . A . . . . - - - - . . C T	. . T . A T . . . . . **C** - - - - - . C T	. . . **A** . . . . - - - - - . **C** T	C	AC	1	2	NA	TATCT	T	5	1	B	B	
E.83_2	170		. . . . C . . . . - - - -	. . T . C T . . . . . **C** - - - - - . G .	. . . **C** . . . . - - - - -	C	C	1	1	NA	TTCG		4	0	B	B	
E.83_3	170	5	. C A T . C . T . T . . - - - -	. C . . C . **T** . **T** . . - - - - - . . .	. **C A T .** C . T . **T** . . **A** - - - - - . **A** .	TT	CATTAA	2	6	α	CC	TC				α	NO
E.83_4	171		. . G . . . . - - - -	. . G . . . . - - - - - . . .	. **G .** . G . . . . . **A** - - - - - . **A** .		GAA	0	3	α	G	G				α	
E.83_5	171		. . G G . . . . - - - -	. . G G . . . . - - - - - . . .	. **G G .** G . . . . . **A** - - - - - . **A** .		GGAA	0	4	α	GG	G				α	
E.83_*			. . G G . . . . - - - -	. . G G . . . . - - - - - . . .	. **G G .** G . . . . . **A** - - - - - . **A** .		GGAA	0	4	α	GG	G				α	
E.95_1	171		. . A T C A . . . . . . - - - -	. . . . C A . . . . . - - - - - . . .	. **A T C** A . . . . . **A** - - - - - . **A** .		ATCAA	0	5	α	CA	A				α	
E.95_2	171	3	. . A . . A . . . . . . - - - -	. . . . . A . . . . . - - - - - . . .	. **A .** . A . . . . . **A** - - - - - . **A** .		AAA	0	3	α	A	A				α	NO
E.95_3	171		. C A . . A . A . . . . - - - -	. C . . . A . **A** . . . - - - - - . . .	**C A .** . A . A . . . **A** - - - - - . **A** .	A	CAAA	1	4	α	CA	AA				α	
E.95_*			. . A T . A . . . . . . - - - -	. . . . . A . . . . . - - - - - . . .	. **A T .** A . . . . . **A** - - - - - . **A** .		ATAA	0	4	α	A	A				α	
E.104_1	171		. . A T C A . . . . . . - - - -	. . . . C A . . . . . - - - - - . . .	. **A T C** A . . . . . **A** - - - - - . **A** .		ATCAA	0	5	α	CA	A				α	
E.104_2	171	3	. . A . . A . . . . . . - - - -	. . . . . A . . . . . - - - - - . . .	. **A .** . A . . . . . **A** - - - - - . **A** .		AAA	0	3	α	A	A				α	NO
E.104_3	171		. C A . . A . A . . . . - - - -	. C . . . A . **A** . . . - - - - - . . .	**C A .** . A . A . . . **A** - - - - - . **A** .	A	CAAA	1	4	α	CA	AA				α	
E.104_*			. . A T . A . . . . . . - - - -	. . . . . A . . . . . - - - - - . . .	. **A T .** A . . . . . **A** - - - - - . **A** .		ATAA	0	4	α	A	A				α	
F.8_1	171		. . A . . . A . . . . . - - - -	. . . . . . **A** . . . . - - - - - . . .	. **A .** . G . A . . . **A** - - - - - . **A** .	A	AAA	1	3	α		AG				**α**	
F.8_2	169		T . . . . - - - -	T . T . . T . . . . . **C** - - - - - . G .	. . . . - - - - -	C		1	0	NA	TTTG		4	0	B	B	
F.8_3	171	5	T C G . . . A . . . . . - - - -	T C G . . . **A** . . . . - - - - - . . .	**C G .** . G . A . . . **A** - - - - - . **A** .	A	CGAA	1	4	α	TCG	GA				α	YES
F.8_4	169		T . . . . - - - -	T . T . . T . . . . . **C** - - - - - . G .	. . . . - - - - -	C		1	0	NA	TTTG		4	0	B	B	
F.8_5	171		. C A . . . A . . . . . - - - -	. C . . . . **A** . . . . - - - - - . . .	**C A .** . G . A . . . **A** - - - - - . **A** .	A	CAAA	1	4	α	C	AG				α	
F.8_*			T . . . . - - - -	T . T . . T . . . . . **C** - - - - - . G .	. . . . - - - - -	C		1	0	NA	TTTG		4	0	B	B	
F.18F_1	171		. . A T . . A . . . . . - - - -	. . . . . . **A** . . . . - - - - - . . .	. **A T .** G . A . . . **A** - - - - - . **A** .	A	ATAA	1	4	α		GA				α	
F.18F_2	171		. . G . A . . . . - - - -	. . G . A T . . . . . **C** - - - - - . G .	. **G . A** . . . . - - - - -	C	GA	1	2	NA	GATG		4	0	B	B	
F.18F_3	171	5	T . . . . - - - -	T . T . . T . . . . . **C** - - - - - . G .	. . . . - - - - -	C		1	0	NA	TTTG		4	0	B	B	NO
F.18F_4	171		. A A T . C . . A G . . . - - - - C . . .	. A . . C . . **G** . . - - - - - **C** . G .	**A A T .** C . . **G** . . **A** - - - - **C** . . .	GC	AATGAC	2	6	α	ACG	C				α	
F.18F_5			T . . . . T - - - -	T . T . . T . . . . . **T** - - - - - . G .	. . . . **T** - - - - -	T	T	1	1	NA	TTTG		4	0	B	B	
F.18R_1	189		T . . . . C T T T . A . G	T . T C . T . . . . . **C C T T T . A** G G	. . . . **C T T T . A .** G	CCTTTA	CTTTA	6	5	NA	TTCTGG	G	6	1	B	B	
				T . T C . . . . **C** - - - - - . G .	. . . . - - - - -	C		1	0	NA	TTCG		4	0	B	B	
F.18R_2	189		T . . . . C T T T . A . G	T . T C . T . . . . . **C C T T T . A** G G	. . . . **C T T T . A .** G	CCTTTA	CTTTA	6	5	NA	TTCTGG	G	6	1	B	B	
		4		T . T C . . . . **C** - - - - - . G .	. . . . - - - - -	C		1	0	NA	TTCG		4	0	B	B	NO
F.18R_3	171		. . A T . . . A G . . G - - - -	. . . . . T . . **G** . **G** - - - - - . . .	. **A T .** . . . . **G** . . **G** - - - - - . **A** .	GG	ATGGA	2	5	α	T					α	
F.18R_4	171		A . . . . - - - -	A . T . . T . . . . . **C** - - - - - . G .	A . . . . - - - - -	C		1	0	NA	ATTG	A	4	1	B	B	
F.18R_*			. . A T . . . . - - - -	. . . . - - - - - . . .	. **A T .** G . . . . . **A** - - - - - . **A** .		ATAA	0	4	α		G				α	
F.94_1	171		. . A T . . A . . . . . - - - -	. . . . . . **A** . . . . - - - - - . . .	. **A T .** G . A . . . **A** - - - - - . **A** .	A	ATAA	1	4	α		GA				α	
F.94_2	171		. C . . . . T - - - -	. C T . . T . . . . . **T** - - - - - . G .	. **C** . . . . **T** - - - - -	T	CT	1	2	NA	CTTG		4	0	B	B	
F.94_3	171	4	. . A . A . . A . . . . - - - -	. . . . A . **A** . . . . - - - - - . . .	. **A . A** G . A . . . **A** - - - - - . **A** .	A	AAAA	1	4	α	A	AG				α	YES
F.94_4	171		. . . . T . . . . - - - -	. . T . . T . **T** . . . **C** - - - - - . G .	. . . . T . . . . - - - - -	TC		2	0	B	TTG	T				B	
F.94_*			. . A T . . C A . . . . - - - -	. . . . . . C **A** . . . - - - - - . . .	. **A T .** G C A . . . **A** - - - - - . **A** .	A	ATAA	1	4	α	C	GCA				α	

G.18_1	171		. A . . . . A . . . . ----. . . .	. A T . . . **A** . . . . . ------. . . .	. **A** . . . G . A . . . **A** ------ . **A** .	A	AAA	1	3	α	TA	AG				α	
G.18_2	169		T . . . . G . ----. . . .	T . T . T . . . . **G C** ------. . G .	. . . . ------ . . .	GC		2	0	B	TTTG					B	
G.18_3	171		. A A . . . A . . . . ----. . . .	. A . . . . **A** . . . . . ------. . . .	. **A A** . . G . A . . . **A** ------ . **A** .	A	AAAA	1	4	α	A	AG				α	
G.18_4	171	5	T . . A . . . . ----. . . .	T . T . A T . . . . . **C** ------. . G .	. . . . **A** . . . . . . ------ . . .	C	A	1	1	NA	TTATG		5	0	B	B	YES
G.18_5	171		. . A T . . . A . . . ----C . . .	. . . . ------**C** . . .	. . **A T** . G . . . . . **A** ---- **C** . **A** .	C	ATACA	1	5	α		G				α	
G.18_*			T . . . A . . . . . . ----. . . .	T . T . . A . . . . . **C** ------. . G .	. . . . . A . . . . . ------ . . .	C		1	0	NA	TTAG	A	4	1	B	B	
G.81_1	171	1	. . A T . . C A . . T . ----. . C .	. . . . . T C **A** . . . **T** . ------. . C .	. . **A T** . C A . . T **A** ------ . **C** .	AT	ATAC	2	4	α	TCC	CAT				α	YES
G.81_*			. . . . ----. . . .	. . T . . T . . . . . **C** ------. . G .	. . . . ------ . . .	C		1	0	NA	TTG		3	0	B	B	
G.84_1	171		. . A T . C . . A G T . G ----. . . .	. . . . . C . . **G T** . **G** ------. . . .	. . **A T** . C . . **G** T . **G** ------ . **A** .	GTG	ATGGA	3	5	α	C	CT				α	
G.84_2	171		A . . . . ----. . . .	A . T . . T . . . . . **C** ------. . G .	A . . . . ------ . . .	C		1	0	NA	ATTG	A	4	1	B	B	
G.84_3	171	4	T . . A . . . . ----. . . .	T . T . A T . . . . . **C** ------. . G .	. . . . **A** . . . . . . ------ . . .	C	A	1	1	NA	TTATG		5	0	B	B	NO
G.84_4	170		. . A . . . A . . . . ----. . . .	. . . . ------. . . .	. . **A** . G . . . . **A** ------ . **A** .		AAA	0	3	α		G				**α**	
G.84_*			T . . A . G . . . . . ----. . . .	T . T . A T G . . . . **C** ------. . G .	. . . . **A** . G . . . ------ . . .	C	A	1	1	NA	TTATGG	G	6	1	B	B	
G.97_1	171		. . G T . . . A . . . ----A . C .	. . G . . . **A** . . . . . ------**A** . C .	. . **G T** . G . A . . . **A** ---- **A** . **C** .	AA	GTAAC	2	5	α	GC	GA				α	
G.97_2	170		A . A . A . . . T . . -----A . . C	A . . . A T . . . . . . ------**A** . G C	A . **A** . **A** . . . . . ------ **A** . . C	-A	AA-A	2	4	α	AATGC	AC	5	2		α	
G.97_3	171		. . A T . A A . . . . ----. . . .	. . . . . A A . . . . . ------. . . .	. . **A T** . A A . . . **A** ------ . **A** .		ATAA	0	4	α	AA	AA				α	
G.97_4	171	6	. . A . . . . ----. . . .	. . . . ------. . . .	. . **A** . G . . . . **A** ------ . **A** .		AAA	0	3	α		G				α	NO
G.97_5	166		. . . T . C . . . . . T ----. . . .	. . T . . C . . . . **T** ------. . G .	. . . **T** . C . . . . **T** ------ . . .	T	TT	1	2	NA	CTG	C	3	1	no box	no box	
G.97_6	171		. . . T . . . . ----. . . .	. . T . . . . ------. . . .	. . . **T** . G . . . . **A** ------ . **A** .		TAA	0	3	α	T	G				α	
G.97_*			. . C T . . . . ----C . . .	. . C . . . . ------**C** . . .	. . **C T** . G . . . . **A** ---- **C** . **A** .	C	CTACA	1	5	α	C	G				α	
G.100_1	171		. A A T . . . A . . . ----. . . .	. A . . . . **A** . . . . . ------. . . .	. **A A T** . G . A . . . **A** ------ . **A** .	A	AATAA	1	5	α	A	GA				α	
G.100_2	169		. . . . ----. . . .	. . T . . T . . . . . **C** ------. . G .	. . . . ------ . . .	C		1	0	NA	TTG		3	0	B	B	
G.100_3	171		. A A T . . A A . . . ----. . . .	. A . . . A **A** . . . . . ------. . . .	. **A A T** . G A A . . . **A** ------ . **A** .	A	AATAA	1	5	α	AA	GAA				α	
G.100_4	169	6	. . . . . . A . . . . ----. . . .	. . T . . T A . . . . **C** ------. . G .	. . . . . . A . . . ------ . . .	C		1	0	B	TTAG	A	4	1	B	B	YES
G.100_5	171		. A A T . . A A . . . ----C . . .	. A . . . A **A** . . . . . ------**C** . . .	. **A A T** . G A A . . . **A** ---- **C** . **A** .	AC	AATACA	2	6	α	AA	GAA				α	
G.100_6	169		. . . . ----. . T .	. . T . . T . . . . . **C** ------. . T .	. . . . ------ . **T** .	C	T	1	1	NA	TTT		3	0	B	B	
G.100_*			. A A T . . . A . . . ----. . . .	. A . . . . **A** . . . . . ------. . . .	. **A A T** . G . A . . . **A** ------ . **A** .	A	AATAA	1	5	α	A	GA				α	
G.105_1	169		T . C . . . . ----. . . .	T . C . . T . . . . . **C** ------. . G .	. . **C** . . . . ------ . . .	C	C	1	1	NA	TTCG		4	0	B	B	
G.105_2	171		. A A . . . A . . . . ----. . . .	. A . . . . **A** . . . . . ------. . . .	. **A A** . . G . A . . . **A** ------ . **A** .	A	AAAA	1	4	α	A	AG				α	
G.105_3	169		T . C . . . . ----. . . .	T . C . . T . . . . . **C** ------. . G .	. . **C** . . . . ------ . . .	C	C	1	1	NA	TTCG		4	0	B	B	
G.105_4	171	6	. A A . . . A . . . . ----. . . .	. A . . . . **A** . . . . . ------. . . .	. **A A** . . G . A . . . **A** ------ . **A** .	A	AAAA	1	4	α	A	AG				α	YES
G.105_5	169		T . . . . ----. . . .	T . T . . T . . . . . **C** ------. . G .	. . . . ------ . . .	C		1	0	NA	TTTG		4	0	B	B	
G.105_6	171		. A A . . A A . . . . ----. . . .	. A . . . A **A** . . . . . ------. . . .	. **A A** . . G A A . . . **A** ------ . **A** .	A	AAAA	1	4	α	AA	AAG				α	
G.105_*			T . . . . T ----. . . .	T . T . . T . . . . . **T** ------. . G .	. . . . **T** ------ . . .	T	T	1	1	NA	TTTG		4	0	B	B	

Note. When essential (in bold) and non-essential positions were discordant PRD was assigned as follows: if |("mismatches if CENP-B box"-" mismatches if pJα motif")|≤2 in the essential positions, and |("mismatches if CENP-B box"-" mismatches if pJα motif")|≥3 in the non-essential positions, the PRD was assigned concordantly to non-essential positions. If instead |("mismatches if CENP-B box"-" mismatches if pJα motif")|≤2 in the essential positions, but |("mismatches if CENP-B box"-" mismatches if pJα motif")|<3 in the non-essential positions, the monomer was considered "no box". Monomers were considered "no box" also in case of equality of mismatches (clones A1.50e, A1.60d, A1.64e).

*-indicates PRD extracted upstream or downstream thefirst or last monomer of the insert, respectively.

Supporting Note Table 4. Illustrative alignments of 465 and 471 gorilla alphoid monomers from gSF1 with A- and B- gorilla consensi by ClustalW. PRDs are highlighted in yellow. : V is A/C/G; B is C/G/T; M is A/C; R is A/G; W is A/T; S is C/G; Y is C/T; K is G/T; F is -/A; I is -/C; J is -/G.

gJ1

[illegible]

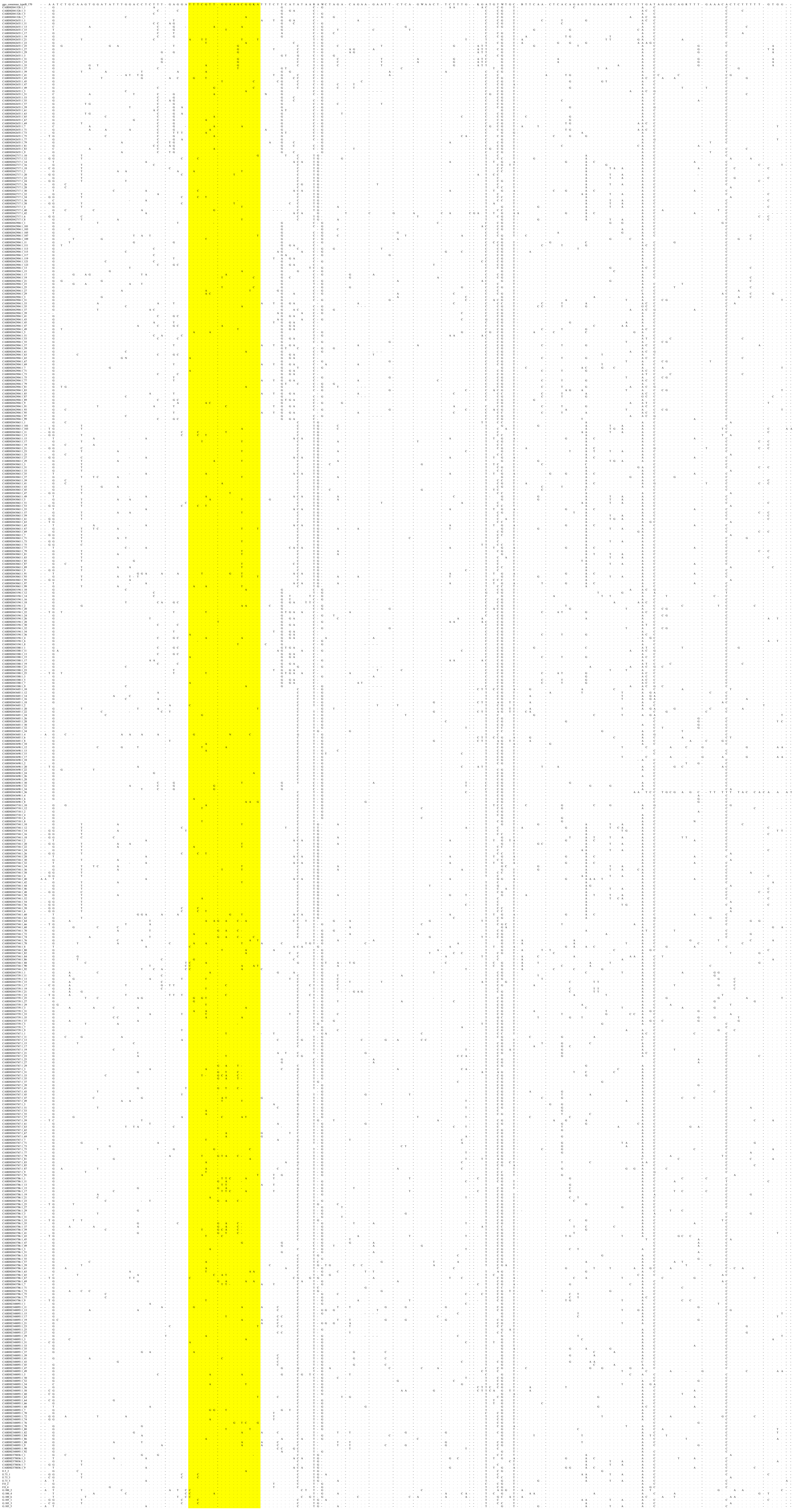

Supporting Note Table 5. Alignments of 74, 76, 60, 100, 71, 59, 70 and 54 gorilla alphoid monomers from gSF2 with A- and B- gorilla consensi by ClustalW. PRDs are highlighted in yellow. V is A/C/G; B is C/G/T; M is A/C; R is A/G; W is A/T; S is C/G; Y is C/T; K is G/T; F is -/A; I is -/C; J is -/G.

gD1.0

[illegible]

gD1.1

[illegible]

gD1.2

[illegible]

gD1.3

[illegible]

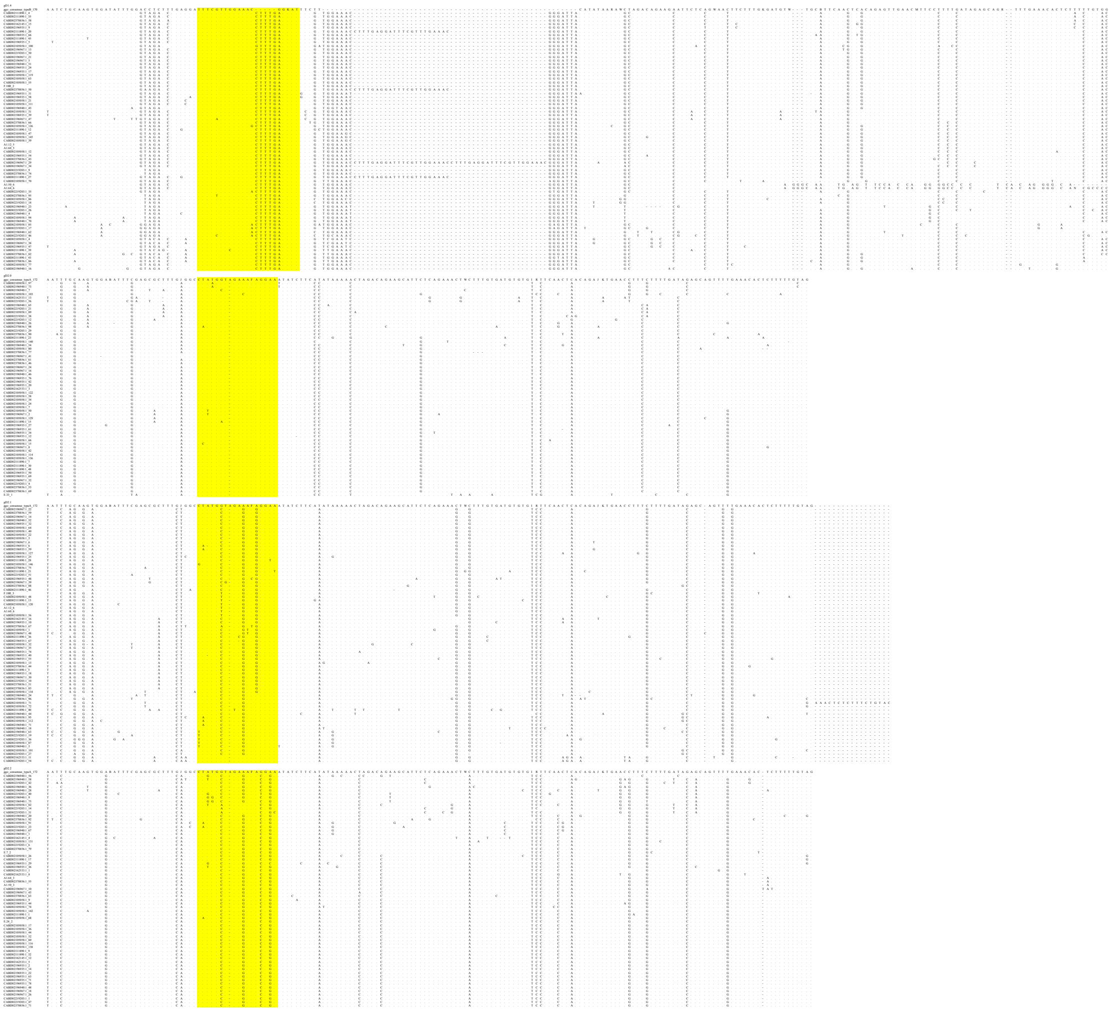

Supporting Note Table 6. Alignments of 21, 26, 27, 14 and 21 gorilla alphoid monomers from gSF3 with A- and B- gorilla consensi by ClustalW. PRDs are highlighted in yellow. V is A/C/G; B is C/G/T; M is A/C; R is A/G; W is A/T; S is C/G; Y is C/T; K is G/T; F is -/A; I is -/C; J is -/G.

gW1

```
ggo_consensus_typeB_170  A A T C T G C A A G T G G A T A T T T G G A C C T C T T T G A G G A T T T C G T T G G A A A C G G K A T T T C T T C A T A T A A R A W C T A G A C A G A A G A A T T C T C A G W A A C T T C T T T G K G A T G T W T G C B T T - C A A C T C A C A G A G T T G A A C M T T C C T T T T G A T A G A G C A G R T T T G A A A C A C T C T T T T T G T G G
CABD02062800.1_58  [illegible]
CABD02062798.1_6  [illegible]
CABD02399115.1_3  [illegible]
CABD02062800.1_45  [illegible]
CABD02399213.1_20  [illegible]
CABD02062800.1_53  [illegible]
CABD02062800.1_29  [illegible]
CABD02062800.1_33  [illegible]
CABD02062800.1_2  [illegible]
CABD02399213.1_12  [illegible]
CABD02399213.1_16  [illegible]
CABD02399213.1_24  [illegible]
CABD02062800.1_41  [illegible]
CABD02062800.1_5  [illegible]
CABD02062800.1_20  [illegible]
CABD02062800.1_15  [illegible]
CABD02399213.1_8  [illegible]
CABD02062800.1_37  [illegible]
CABD02062800.1_25  [illegible]
CABD02399213.1_4  [illegible]
CABD02062800.1_11  [illegible]
```

gW2

```
ggo_consensus_typeB_170  A A T C T G C A A G T G G A T A T T T G G A C C T C T T T G A G G A T T T C G T T G G A A A C G G K - A T T T C T T C A T A T A A R A W C T A G A C A G A A G A A T T C T C A G W A A C T T C T T T G K G A T G T W T G C B T T C A A C T C A C A G A G T T G A A C M T T C C T T T T G A T A G A G C A G R T T T G A A A C A C T C T T T T T G T G G
CABD02062797.1_2  [illegible]
CABD02062798.1_13  [illegible]
CABD02062797.1_6  [illegible]
CABD02399115.1_9  [illegible]
CABD02062798.1_7  [illegible]
CABD02399115.1_4  [illegible]
CABD02062800.1_59  [illegible]
CABD02062800.1_3  [illegible]
CABD02062800.1_38  [illegible]
CABD02062800.1_46  [illegible]
CABD02399213.1_5  [illegible]
CABD02062800.1_21  [illegible]
CABD02399213.1_13  [illegible]
CABD02062800.1_26  [illegible]
CABD02062800.1_54  [illegible]
CABD02399213.1_9  [illegible]
CABD02062800.1_6  [illegible]
CABD02399213.1_21  [illegible]
CABD02399213.1_25  [illegible]
CABD02399213.1_17  [illegible]
E.27_1  [illegible]
CABD02062800.1_12  [illegible]
CABD02062800.1_16  [illegible]
CABD02062800.1_30  [illegible]
CABD02062800.1_34  [illegible]
CABD02062800.1_42  [illegible]
```

gW3

```
ggo_consensus_typeB_170  A A T C T G C A A G T G G A T A T T T G G A C C T C T T T G A G G A T T T C G T T G G A A A C G G K - A - T T T C T T C A T A T A A R A W C T A G A C A G A A G A A T T C T C A G W A A C T T C T T T G K G A T G T W T G C B T T C A A C T C A C A G A G T T G A A C M T T C C T T T T G A T A G A G C A G R T T T G A A A C A C T C T T T T T G T G G
CABD02062800.1_17  [illegible]
CABD02062800.1_22  [illegible]
CABD02399213.1_6  [illegible]
E.27_2  [illegible]
CABD02062800.1_13  [illegible]
CABD02062800.1_35  [illegible]
CABD02062800.1_18  [illegible]
CABD02062800.1_23  [illegible]
CABD02062800.1_31  [illegible]
CABD02062800.1_43  [illegible]
CABD02399213.1_10  [illegible]
CABD02062800.1_27  [illegible]
CABD02399213.1_14  [illegible]
CABD02399213.1_18  [illegible]
CABD02062800.1_7  [illegible]
CABD02399213.1_2  [illegible]
CABD02062800.1_9  [illegible]
CABD02399213.1_22  [illegible]
CABD02062800.1_39  [illegible]
CABD02062800.1_47  [illegible]
CABD02062800.1_55  [illegible]
CABD02062800.1_60  [illegible]
CABD02399115.1_10  [illegible]
CABD02062797.1_3  [illegible]
CABD02062798.1_8  [illegible]
CABD02062797.1_7  [illegible]
CABD02062798.1_1  [illegible]
```

gW4

```
ggo_consensus_typeA_172  A A T T T G C A A G T G G A B A T T T C G A G C G C T T T G J G G C C T A T G G T A G A A A F A G G A A A T A T C T T C A T A T A A A A A C T A G A C A G A A G C A T T C T C A G A A A C T W C T T T G T G A T G T G T G I R T T C A A C T C A C A G A J K T G A A C C T T T C T T T G A T A G A G C A G T T T G A A A C A C T C T T T T T G T A G -
CABD02062797.1_4  [illegible]
CABD02062798.1_9  [illegible]
CABD02062797.1_8  [illegible]
CABD02062798.1_2  [illegible]
CABD02062800.1_61  [illegible]
CABD02062800.1_48  [illegible]
CABD02062800.1_56  [illegible]
CABD02062800.1_63  [illegible]
CABD02399115.1_6  [illegible]
CABD02062800.1_51  [illegible]
CABD02062798.1_11  [illegible]
CABD02062798.1_4  [illegible]
CABD02062798.1_15  [illegible]
CABD02399115.1_1  [illegible]
```

gW5

```
ggo_consensus_typeA_172  A A T T T G C A A G T G G A B A T T T C G A G C G C T T T G J G G C C T A T G G T A G A A A F A G G A A A T A T C T T C A T A T A A A A A C T A G A C A G A A G C A T T C T C A G A A A C T W C T T T G T G A T G T G T G I R T T C A A C T C A C A G A J K T G A A C C T T T C T T T G A T A G A G C A G T T T G A A A C A C T C T T T T T G T A G
CABD02062800.1_19  [illegible]
CABD02062800.1_44  [illegible]
CABD02399213.1_7  [illegible]
CABD02399213.1_19  [illegible]
CABD02062800.1_4  [illegible]
CABD02399213.1_3  [illegible]
CABD02062800.1_10  [illegible]
CABD02399213.1_11  [illegible]
CABD02062800.1_1  [illegible]
CABD02399213.1_23  [illegible]
CABD02062800.1_32  [illegible]
CABD02399213.1_15  [illegible]
CABD02062800.1_28  [illegible]
CABD02062800.1_49  [illegible]
CABD02062800.1_40  [illegible]
CABD02062800.1_24  [illegible]
CABD02062800.1_36  [illegible]
E.27_3  [illegible]
CABD02062800.1_14  [illegible]
CABD02062800.1_57  [illegible]
CABD02399115.1_7  [illegible]
```

Supporting Note Table 7. Alphoid composition and organiation of the WGSSs and plasmids (16+7) containing exclusively monomers from the blue branches of the phylogenetic tree.

CABD02041326.1_1		
CABD02041326.1_2		
CABD02041326.1_3	W1R1	
CABD02041326.1_4		
CABD02041326.1_5		
CABD02041326.1_6		
CABD02041326.1_7	D1	
CABD02041326.1_8		
CABD02042655.1_1	W1R1	
CABD02042655.1_2		
CABD02042655.1_3		
CABD02042655.1_4		
CABD02042655.1_5	D1	
CABD02042655.1_6		
CABD02042655.1_7		
CABD02042655.1_8		
CABD02042655.1_9		
CABD02042655.1_10		
CABD02042655.1_11	W1R1	
CABD02042655.1_12		
CABD02042655.1_13		
CABD02042655.1_14		
CABD02042655.1_15	W1	
CABD02042655.1_16		
CABD02042655.1_17	W1R1	
CABD02042655.1_18		
CABD02042655.1_19	W1R1	
CABD02042655.1_20		
CABD02042655.1_21		
CABD02042655.1_22		
CABD02042655.1_23		
CABD02042655.1_24		
CABD02042655.1_25		
CABD02042655.1_26		
CABD02042655.1_27		
CABD02042655.1_28		
CABD02042655.1_29		
CABD02042655.1_30		
CABD02042655.1_31		
CABD02042655.1_32		
CABD02042655.1_33		
CABD02042655.1_34		
CABD02042655.1_35		
CABD02042655.1_36		
CABD02042655.1_37	W1R1	
CABD02042655.1_38		
CABD02042655.1_39		
CABD02042655.1_40		
CABD02042655.1_41	D1	
CABD02042655.1_42		
CABD02042655.1_43		
CABD02042655.1_44		
CABD02042655.1_45		
CABD02042655.1_46		
CABD02042655.1_47		
CABD02042655.1_48		
CABD02042655.1_49	W1	
CABD02042655.1_50		
CABD02042655.1_51	W1R1	
CABD02042655.1_52		
CABD02042655.1_53		
CABD02042655.1_54		
CABD02042655.1_55	W1R1	
CABD02042655.1_56		
CABD02042655.1_57	W1	
CABD02042655.1_58		
CABD02042655.1_59	W1R1	
CABD02042655.1_60		
CABD02042655.1_61	W1R1	
CABD02042655.1_62		
CABD02042655.1_63	W1R1	
CABD02042655.1_64		
CABD02042655.1_65	W1R1	
CABD02042655.1_66		
CABD02042655.1_67		
CABD02042655.1_68		
CABD02042655.1_69		
CABD02042655.1_70		
CABD02042655.1_71	W1R1	
CABD02042655.1_72		
CABD02042655.1_73		
CABD02042655.1_74		
CABD02042655.1_75	W1R1	
CABD02042655.1_76		
CABD02042655.1_77	W1R1	
CABD02042655.1_78		
CABD02042655.1_79	W1	
CABD02042655.1_80		
CABD02042655.1_81	W1R1	
CABD02042655.1_82		
CABD02042655.1_83	W1R1	
CABD02042717.1_1		
CABD02042717.1_2	W3R1	
CABD02042717.1_3		
CABD02042717.1_4	W3	
CABD02042717.1_5		
CABD02042717.1_6		
CABD02042717.1_7		
CABD02042717.1_8	W3	
CABD02042717.1_9		
CABD02042717.1_10	W1W3R1	
CABD02042717.1_11		
CABD02042717.1_12		
CABD02042717.1_13		
CABD02042717.1_14		
CABD02042717.1_15		
CABD02042717.1_16		
CABD02042717.1_17		
CABD02042717.1_18	W3	
CABD02042717.1_19		
CABD02042717.1_20	W3R1	
CABD02042717.1_21		
CABD02042717.1_22	W3	
CABD02042717.1_23		
CABD02042717.1_24		
CABD02042717.1_25		
CABD02042717.1_26	W3	
CABD02042717.1_27		
CABD02042717.1_28		
CABD02042717.1_29		
CABD02042717.1_30		
CABD02042717.1_31		
CABD02042717.1_32	W3	
CABD02042717.1_33		
CABD02042717.1_34		
CABD02042717.1_35		
CABD02042717.1_36	W3	
CABD02042717.1_37		
CABD02042717.1_38	W3R1	
CABD02042717.1_39		
CABD02042717.1_40	D1	
CABD02042717.1_41		
CABD02042717.1_42		
CABD02042904.1_1		
CABD02042904.1_2		
CABD02042904.1_3		
CABD02042904.1_4		
CABD02042904.1_5		
CABD02042904.1_6		
CABD02042904.1_7		
CABD02042904.1_8		
CABD02042904.1_9		
CABD02042904.1_10		
CABD02042904.1_11		
CABD02042904.1_12		
CABD02042904.1_13	D1	
CABD02042904.1_14		
CABD02042904.1_15		
CABD02042904.1_16		
CABD02042904.1_17		
CABD02042904.1_18		
CABD02042904.1_19		
CABD02042904.1_20		
CABD02042904.1_21		
CABD02042904.1_22		
CABD02042904.1_23		
CABD02042904.1_24		
CABD02042904.1_25		
CABD02042904.1_26		
CABD02042904.1_27		
CABD02042904.1_28		
CABD02042904.1_29		
CABD02042904.1_30		
CABD02042904.1_31		
CABD02042904.1_32		
CABD02042904.1_33	D1	
CABD02042904.1_34		
CABD02042904.1_35		
CABD02042904.1_36		
CABD02042904.1_37		
CABD02042904.1_38		
CABD02042904.1_39		
CABD02042904.1_40		
CABD02042904.1_41	W1R1	
CABD02042904.1_42		
CABD02042904.1_43		
CABD02042904.1_44		
CABD02042904.1_45		
CABD02042904.1_46		
CABD02042904.1_47		
CABD02042904.1_48		
CABD02042904.1_49		
CABD02042904.1_50		
CABD02042904.1_51		
CABD02042904.1_52		
CABD02042904.1_53	W1	
CABD02042904.1_54		
CABD02042904.1_55		
CABD02042904.1_56		
CABD02042904.1_57		
CABD02042904.1_58		
CABD02042904.1_59		
CABD02042904.1_60		
CABD02042904.1_61	D1	
CABD02042904.1_62		
CABD02042904.1_63	W1R1	
CABD02042904.1_64		
CABD02042904.1_65		
CABD02042904.1_66		
CABD02042904.1_67		
CABD02042904.1_68		
CABD02042904.1_69	W1R1	
CABD02042904.1_70		
CABD02042904.1_71		
CABD02042904.1_72		
CABD02042904.1_73	W1	
CABD02042904.1_74		
CABD02042904.1_75		
CABD02042904.1_76		
CABD02042904.1_77	W1R1	
CABD02042904.1_78		
CABD02042904.1_79		
CABD02042904.1_80		
CABD02042904.1_81	D1	
CABD02042904.1_82		
CABD02042904.1_83		
CABD02042904.1_84		
CABD02042904.1_85	D1	
CABD02042904.1_86		
CABD02042904.1_87		
CABD02042904.1_88		
CABD02042904.1_89	W1R1	
CABD02042904.1_90		
CABD02042904.1_91		
CABD02042904.1_92		
CABD02042904.1_93		
CABD02042904.1_94		
CABD02042904.1_95	D1	
CABD02042904.1_96		
CABD02042904.1_97	D1	
CABD02042904.1_98		
CABD02042904.1_99		
CABD02042904.1_100		
CABD02042904.1_101		
CABD02042904.1_102		
CABD02042904.1_103		
CABD02042904.1_104		
CABD02042904.1_105		
CABD02042904.1_106		
CABD02042904.1_107	W1	
CABD02042904.1_108		
CABD02042904.1_109	D1	
CABD02042904.1_110		
CABD02042904.1_111		
CABD02042904.1_112		
CABD02042904.1_113		
CABD02042904.1_114		
CABD02042904.1_115		
CABD02042904.1_116		
CABD02042904.1_117		
CABD02042904.1_118		
CABD02042904.1_119		
CABD02042904.1_120		
CABD02042904.1_121		
CABD02042904.1_122		
CABD02042904.1_123	W1R1	
CABD02042904.1_124		
CABD02043063.1_1		
CABD02043063.1_2		
CABD02043063.1_3		
CABD02043063.1_4		
CABD02043063.1_5	W3	
CABD02043063.1_6		
CABD02043063.1_7		
CABD02043063.1_8		
CABD02043063.1_9		
CABD02043063.1_10		
CABD02043063.1_11	W3	
CABD02043063.1_12		
CABD02043063.1_13		
CABD02043063.1_14		
CABD02043063.1_15		
CABD02043063.1_16		
CABD02043063.1_17	W3	
CABD02043063.1_18		
CABD02043063.1_19	W3R1	
CABD02043063.1_20		
CABD02043063.1_21		
CABD02043063.1_22		
CABD02043063.1_23	W3	
CABD02043063.1_24		
CABD02043063.1_25		
CABD02043063.1_26		
CABD02043063.1_27		
CABD02043063.1_28		
CABD02043063.1_29	W3	
CABD02043063.1_30		
CABD02043063.1_31		
CABD02043063.1_32		
CABD02043063.1_33		
CABD02043063.1_34		
CABD02043063.1_35		
CABD02043063.1_36		
CABD02043063.1_37		
CABD02043063.1_38		
CABD02043063.1_39	W3R1	
CABD02043063.1_40		
CABD02043063.1_41		
CABD02043063.1_42		
CABD02043063.1_43		
CABD02043063.1_44		
CABD02043063.1_45		
CABD02043063.1_46		
CABD02043063.1_47		
CABD02043063.1_48		
CABD02043063.1_49		
CABD02043063.1_50		
CABD02043063.1_51	W3R1	
CABD02043063.1_52		
CABD02043063.1_53		
CABD02043063.1_54		
CABD02043063.1_55		
CABD02043063.1_56		
CABD02043063.1_57	W3R1	
CABD02043063.1_58		
CABD02043063.1_59	W3	
CABD02043063.1_60		
CABD02043063.1_61	W3	
CABD02043063.1_62		
CABD02043063.1_63		
CABD02043063.1_64		
CABD02043063.1_65		
CABD02043063.1_66		
CABD02043063.1_67	W3	
CABD02043063.1_68		
CABD02043063.1_69		
CABD02043063.1_70		
CABD02043063.1_71	W3R1	
CABD02043063.1_72		
CABD02043063.1_73	W3R1	
CABD02043063.1_74		
CABD02043063.1_75		
CABD02043063.1_76		
CABD02043063.1_77		
CABD02043063.1_78		
CABD02043063.1_79	W3	
CABD02043063.1_80		
CABD02043063.1_81	W3	
CABD02043063.1_82		
CABD02043063.1_83	W3	
CABD02043063.1_84		
CABD02043063.1_85		
CABD02043063.1_86		
CABD02043063.1_87	W3	
CABD02043063.1_88		
CABD02043063.1_89		
CABD02043063.1_90		
CABD02043063.1_91		
CABD02043063.1_92		
CABD02043063.1_93	W3	
CABD02043063.1_94		
CABD02043063.1_95		
CABD02043063.1_96		
CABD02043063.1_97		
CABD02043063.1_98		
CABD02043063.1_99		
CABD02043063.1_100		
CABD02043063.1_101		
CABD02043063.1_102		
CABD02043063.1_103	W3	
CABD02043194.1_1		
CABD02043194.1_2		
CABD02043194.1_3		
CABD02043194.1_4		
CABD02043194.1_5		
CABD02043194.1_6		
CABD02043194.1_7		
CABD02043194.1_8		
CABD02043194.1_9		
CABD02043194.1_10	D1	
CABD02043194.1_11		
CABD02043194.1_12		
CABD02043194.1_13		
CABD02043194.1_14	W1R1	
CABD02043194.1_15		
CABD02043194.1_16		
CABD02043194.1_17		
CABD02043194.1_18	W1R1	
CABD02043194.1_19		
CABD02043194.1_20		
CABD02043194.1_21		
CABD02043194.1_22		
CABD02043194.1_23		
CABD02043194.1_24		
CABD02043194.1_25		
CABD02043194.1_26		
CABD02043194.1_27		
CABD02043194.1_28		
CABD02043194.1_29		
CABD02043194.1_30	W1R1	
CABD02043194.1_31		
CABD02043194.1_32		
CABD02043194.1_33		
CABD02043194.1_34		
CABD02043194.1_35		
CABD02043194.1_36		
CABD02043380.1_1	W1R1	
CABD02043380.1_2		
CABD02043380.1_3		
CABD02043380.1_4		
CABD02043380.1_5		
CABD02043380.1_6		
CABD02043380.1_7		
CABD02043380.1_8		
CABD02043380.1_9	D1	
CABD02043380.1_10		
CABD02043380.1_11	D1	
CABD02043380.1_12		
CABD02043380.1_13	W1R1	
CABD02043380.1_14		
CABD02043380.1_15		
CABD02043380.1_16		
CABD02043380.1_17		
CABD02043380.1_18		
CABD02043380.1_19	W1	
CABD02043380.1_20		
CABD02043380.1_21	D1	
CABD02043380.1_22		
CABD02043380.1_23		
CABD02043380.1_24		
CABD02043380.1_25		
CABD02043380.1_26		
CABD02043685.1_1		
CABD02043685.1_2		
CABD02043685.1_3		
CABD02043685.1_4	W1	
CABD02043685.1_5		
CABD02043685.1_6		
CABD02043685.1_7		
CABD02043685.1_8		
CABD02043685.1_9		
CABD02043685.1_10		
CABD02043685.1_11		
CABD02043685.1_12		
CABD02043685.1_13		
CABD02043685.1_14		
CABD02043685.1_15		
CABD02043685.1_16		
CABD02043685.1_17		
CABD02043685.1_18		
CABD02043685.1_19		
CABD02043685.1_20		
CABD02043685.1_21		
CABD02043685.1_22		
CABD02043685.1_23		
CABD02043685.1_24		
CABD02043685.1_25		
CABD02043685.1_26		
CABD02043685.1_27		
CABD02043685.1_28		
CABD02043685.1_29		
CABD02043685.1_30		
CABD02043685.1_31		
CABD02043685.1_32		
CABD02043685.1_33		
CABD02043685.1_34		
CABD02043690.1_1		
CABD02043690.1_2		
CABD02043690.1_3		
CABD02043690.1_4		
CABD02043690.1_5		
CABD02043690.1_6		
CABD02043690.1_7		
CABD02043690.1_8		
CABD02043690.1_9		
CABD02043690.1_10		
CABD02043690.1_11		
CABD02043690.1_12	D1	
CABD02043690.1_13		
CABD02043690.1_14		
CABD02043690.1_15		
CABD02043690.1_16		
CABD02043690.1_17		
CABD02043690.1_18		
CABD02043690.1_19		
CABD02043690.1_20		
CABD02043690.1_21		
CABD02043690.1_22		
CABD02043690.1_23		
CABD02043690.1_24		
CABD02043690.1_25		
CABD02043690.1_26		
CABD02043690.1_27		
CABD02043690.1_28		
CABD02043690.1_29		
CABD02043690.1_30	W1R1	
CABD02043690.1_31		
CABD02043690.1_32	W1R1	
CABD02043690.1_33		
CABD02043690.1_34	W1R1	
CABD02043690.1_35		
CABD02043690.1_36	W1R1	
CABD02043710.1_1		
CABD02043710.1_2		
CABD02043710.1_3		
CABD02043710.1_4		
CABD02043710.1_5		
CABD02043710.1_6		
CABD02043710.1_7		
CABD02043710.1_8		
CABD02043710.1_9		
CABD02043710.1_10		
CABD02043710.1_11		
CABD02043710.1_12		
CABD02043744.1_1		
CABD02043744.1_2		
CABD02043744.1_3		
CABD02043744.1_4	W3	
CABD02043744.1_5		
CABD02043744.1_6		
CABD02043744.1_7		
CABD02043744.1_8		
CABD02043744.1_9		
CABD02043744.1_10	W3	
CABD02043744.1_11		
CABD02043744.1_12	W3R1	
CABD02043744.1_13		
CABD02043744.1_14		
CABD02043744.1_15		
CABD02043744.1_16		
CABD02043744.1_17		
CABD02043744.1_18		
CABD02043744.1_19		
CABD02043744.1_20	W3	
CABD02043744.1_21		
CABD02043744.1_22		
CABD02043744.1_23		
CABD02043744.1_24	W3	
CABD02043744.1_25		
CABD02043744.1_26		
CABD02043744.1_27		
CABD02043744.1_28		

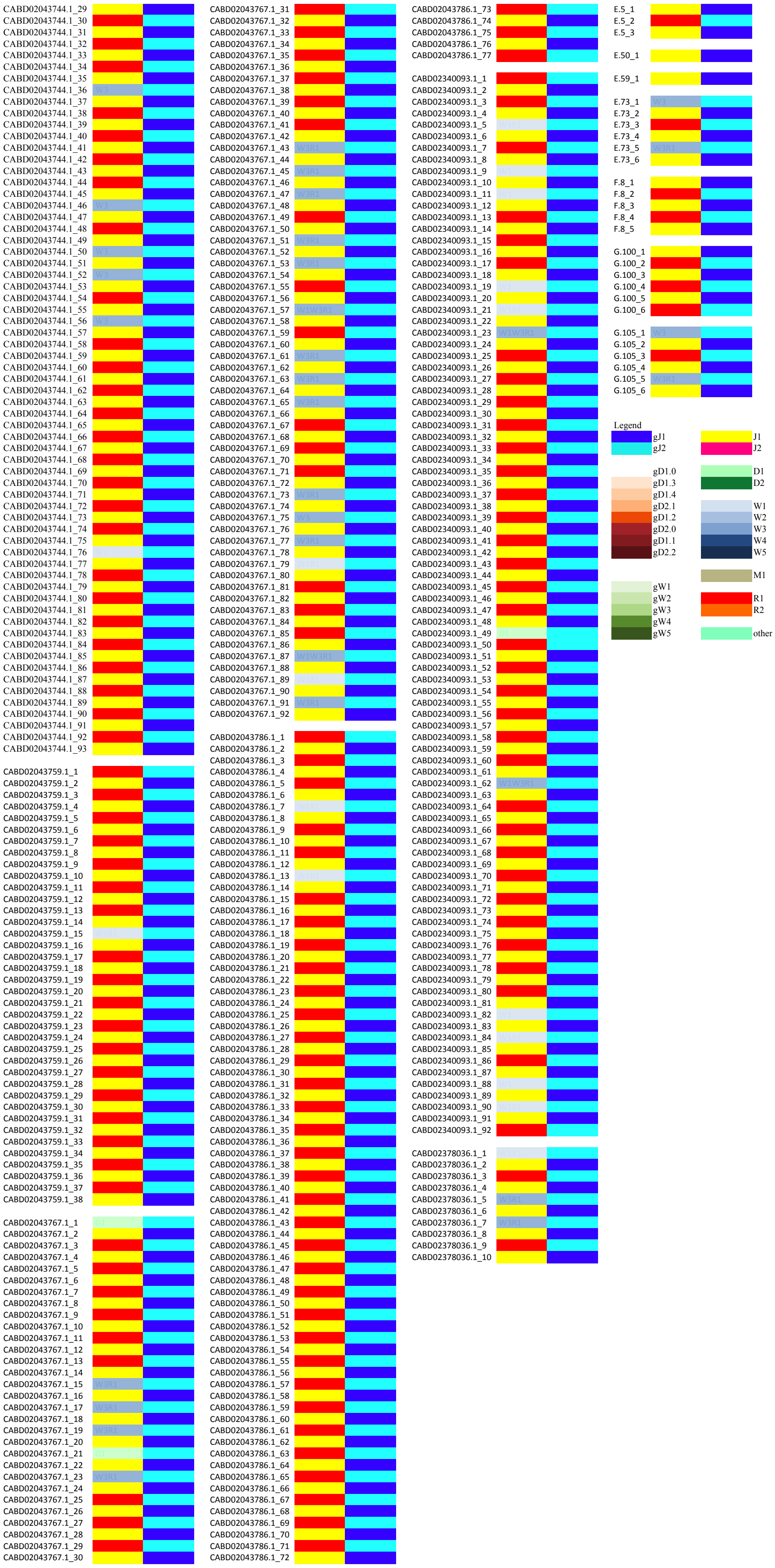

CABD02043744.1_29
CABD02043744.1_30
CABD02043744.1_31
CABD02043744.1_32
CABD02043744.1_33
CABD02043744.1_34
CABD02043744.1_35
CABD02043744.1_36
CABD02043744.1_37
CABD02043744.1_38
CABD02043744.1_39
CABD02043744.1_40
CABD02043744.1_41
CABD02043744.1_42
CABD02043744.1_43
CABD02043744.1_44
CABD02043744.1_45
CABD02043744.1_46
CABD02043744.1_47
CABD02043744.1_48
CABD02043744.1_49
CABD02043744.1_50
CABD02043744.1_51
CABD02043744.1_52
CABD02043744.1_53
CABD02043744.1_54
CABD02043744.1_55
CABD02043744.1_56
CABD02043744.1_57
CABD02043744.1_58
CABD02043744.1_59
CABD02043744.1_60
CABD02043744.1_61
CABD02043744.1_62
CABD02043744.1_63
CABD02043744.1_64
CABD02043744.1_65
CABD02043744.1_66
CABD02043744.1_67
CABD02043744.1_68
CABD02043744.1_69
CABD02043744.1_70
CABD02043744.1_71
CABD02043744.1_72
CABD02043744.1_73
CABD02043744.1_74
CABD02043744.1_75
CABD02043744.1_76
CABD02043744.1_77
CABD02043744.1_78
CABD02043744.1_79
CABD02043744.1_80
CABD02043744.1_81
CABD02043744.1_82
CABD02043744.1_83
CABD02043744.1_84
CABD02043744.1_85
CABD02043744.1_86
CABD02043744.1_87
CABD02043744.1_88
CABD02043744.1_89
CABD02043744.1_90
CABD02043744.1_91
CABD02043744.1_92
CABD02043744.1_93
CABD02043759.1_1
CABD02043759.1_2
CABD02043759.1_3
CABD02043759.1_4
CABD02043759.1_5
CABD02043759.1_6
CABD02043759.1_7
CABD02043759.1_8
CABD02043759.1_9
CABD02043759.1_10
CABD02043759.1_11
CABD02043759.1_12
CABD02043759.1_13
CABD02043759.1_14
CABD02043759.1_15
CABD02043759.1_16
CABD02043759.1_17
CABD02043759.1_18
CABD02043759.1_19
CABD02043759.1_20
CABD02043759.1_21
CABD02043759.1_22
CABD02043759.1_23
CABD02043759.1_24
CABD02043759.1_25
CABD02043759.1_26
CABD02043759.1_27
CABD02043759.1_28
CABD02043759.1_29
CABD02043759.1_30
CABD02043759.1_31
CABD02043759.1_32
CABD02043759.1_33
CABD02043759.1_34
CABD02043759.1_35
CABD02043759.1_36
CABD02043759.1_37
CABD02043759.1_38
CABD02043767.1_1
CABD02043767.1_2
CABD02043767.1_3
CABD02043767.1_4
CABD02043767.1_5
CABD02043767.1_6
CABD02043767.1_7
CABD02043767.1_8
CABD02043767.1_9
CABD02043767.1_10
CABD02043767.1_11
CABD02043767.1_12
CABD02043767.1_13
CABD02043767.1_14
CABD02043767.1_15
CABD02043767.1_16
CABD02043767.1_17
CABD02043767.1_18
CABD02043767.1_19
CABD02043767.1_20
CABD02043767.1_21
CABD02043767.1_22
CABD02043767.1_23
CABD02043767.1_24
CABD02043767.1_25
CABD02043767.1_26
CABD02043767.1_27
CABD02043767.1_28
CABD02043767.1_29
CABD02043767.1_30
CABD02043767.1_31
CABD02043767.1_32
CABD02043767.1_33
CABD02043767.1_34
CABD02043767.1_35
CABD02043767.1_36
CABD02043767.1_37
CABD02043767.1_38
CABD02043767.1_39
CABD02043767.1_40
CABD02043767.1_41
CABD02043767.1_42
CABD02043767.1_43
CABD02043767.1_44
CABD02043767.1_45
CABD02043767.1_46
CABD02043767.1_47
CABD02043767.1_48
CABD02043767.1_49
CABD02043767.1_50
CABD02043767.1_51
CABD02043767.1_52
CABD02043767.1_53
CABD02043767.1_54
CABD02043767.1_55
CABD02043767.1_56
CABD02043767.1_57
CABD02043767.1_58
CABD02043767.1_59
CABD02043767.1_60
CABD02043767.1_61
CABD02043767.1_62
CABD02043767.1_63
CABD02043767.1_64
CABD02043767.1_65
CABD02043767.1_66
CABD02043767.1_67
CABD02043767.1_68
CABD02043767.1_69
CABD02043767.1_70
CABD02043767.1_71
CABD02043767.1_72
CABD02043767.1_73
CABD02043767.1_74
CABD02043767.1_75
CABD02043767.1_76
CABD02043767.1_77
CABD02043767.1_78
CABD02043767.1_79
CABD02043767.1_80
CABD02043767.1_81
CABD02043767.1_82
CABD02043767.1_83
CABD02043767.1_84
CABD02043767.1_85
CABD02043767.1_86
CABD02043767.1_87
CABD02043767.1_88
CABD02043767.1_89
CABD02043767.1_90
CABD02043767.1_91
CABD02043767.1_92
CABD02043786.1_1
CABD02043786.1_2
CABD02043786.1_3
CABD02043786.1_4
CABD02043786.1_5
CABD02043786.1_6
CABD02043786.1_7
CABD02043786.1_8
CABD02043786.1_9
CABD02043786.1_10
CABD02043786.1_11
CABD02043786.1_12
CABD02043786.1_13
CABD02043786.1_14
CABD02043786.1_15
CABD02043786.1_16
CABD02043786.1_17
CABD02043786.1_18
CABD02043786.1_19
CABD02043786.1_20
CABD02043786.1_21
CABD02043786.1_22
CABD02043786.1_23
CABD02043786.1_24
CABD02043786.1_25
CABD02043786.1_26
CABD02043786.1_27
CABD02043786.1_28
CABD02043786.1_29
CABD02043786.1_30
CABD02043786.1_31
CABD02043786.1_32
CABD02043786.1_33
CABD02043786.1_34
CABD02043786.1_35
CABD02043786.1_36
CABD02043786.1_37
CABD02043786.1_38
CABD02043786.1_39
CABD02043786.1_40
CABD02043786.1_41
CABD02043786.1_42
CABD02043786.1_43
CABD02043786.1_44
CABD02043786.1_45
CABD02043786.1_46
CABD02043786.1_47
CABD02043786.1_48
CABD02043786.1_49
CABD02043786.1_50
CABD02043786.1_51
CABD02043786.1_52
CABD02043786.1_53
CABD02043786.1_54
CABD02043786.1_55
CABD02043786.1_56
CABD02043786.1_57
CABD02043786.1_58
CABD02043786.1_59
CABD02043786.1_60
CABD02043786.1_61
CABD02043786.1_62
CABD02043786.1_63
CABD02043786.1_64
CABD02043786.1_65
CABD02043786.1_66
CABD02043786.1_67
CABD02043786.1_68
CABD02043786.1_69
CABD02043786.1_70
CABD02043786.1_71
CABD02043786.1_72
CABD02043786.1_73
CABD02043786.1_74
CABD02043786.1_75
CABD02043786.1_76
CABD02043786.1_77
CABD02340093.1_1
CABD02340093.1_2
CABD02340093.1_3
CABD02340093.1_4
CABD02340093.1_5
CABD02340093.1_6
CABD02340093.1_7
CABD02340093.1_8
CABD02340093.1_9
CABD02340093.1_10
CABD02340093.1_11
CABD02340093.1_12
CABD02340093.1_13
CABD02340093.1_14
CABD02340093.1_15
CABD02340093.1_16
CABD02340093.1_17
CABD02340093.1_18
CABD02340093.1_19
CABD02340093.1_20
CABD02340093.1_21
CABD02340093.1_22
CABD02340093.1_23
CABD02340093.1_24
CABD02340093.1_25
CABD02340093.1_26
CABD02340093.1_27
CABD02340093.1_28
CABD02340093.1_29
CABD02340093.1_30
CABD02340093.1_31
CABD02340093.1_32
CABD02340093.1_33
CABD02340093.1_34
CABD02340093.1_35
CABD02340093.1_36
CABD02340093.1_37
CABD02340093.1_38
CABD02340093.1_39
CABD02340093.1_40
CABD02340093.1_41
CABD02340093.1_42
CABD02340093.1_43
CABD02340093.1_44
CABD02340093.1_45
CABD02340093.1_46
CABD02340093.1_47
CABD02340093.1_48
CABD02340093.1_49
CABD02340093.1_50
CABD02340093.1_51
CABD02340093.1_52
CABD02340093.1_53
CABD02340093.1_54
CABD02340093.1_55
CABD02340093.1_56
CABD02340093.1_57
CABD02340093.1_58
CABD02340093.1_59
CABD02340093.1_60
CABD02340093.1_61
CABD02340093.1_62
CABD02340093.1_63
CABD02340093.1_64
CABD02340093.1_65
CABD02340093.1_66
CABD02340093.1_67
CABD02340093.1_68
CABD02340093.1_69
CABD02340093.1_70
CABD02340093.1_71
CABD02340093.1_72
CABD02340093.1_73
CABD02340093.1_74
CABD02340093.1_75
CABD02340093.1_76
CABD02340093.1_77
CABD02340093.1_78
CABD02340093.1_79
CABD02340093.1_80
CABD02340093.1_81
CABD02340093.1_82
CABD02340093.1_83
CABD02340093.1_84
CABD02340093.1_85
CABD02340093.1_86
CABD02340093.1_87
CABD02340093.1_88
CABD02340093.1_89
CABD02340093.1_90
CABD02340093.1_91
CABD02340093.1_92
CABD02378036.1_1
CABD02378036.1_2
CABD02378036.1_3
CABD02378036.1_4
CABD02378036.1_5
CABD02378036.1_6
CABD02378036.1_7
CABD02378036.1_8
CABD02378036.1_9
CABD02378036.1_10
E.5_1
E.5_2
E.5_3
E.50_1
E.59_1
E.73_1
E.73_2
E.73_3
E.73_4
E.73_5
E.73_6
F.8_1
F.8_2
F.8_3
F.8_4
F.8_5
G.100_1
G.100_2
G.100_3
G.100_4
G.100_5
G.100_6
G.105_1
G.105_2
G.105_3
G.105_4
G.105_5
G.105_6
Legend
gJ1
gJ2
J1
J2
gD1.0
gD1.3
gD1.4
gD2.1
gD1.2
gD2.0
gD1.1
gD2.2
D1
D2
W1
W2
W3
W4
W5
M1
gW1
gW2
gW3
gW4
gW5
R1
R2
other

Supporting Note Table 8. Alphoid composition and organiation of the WGSSs and plasmids (10+11) containing exclusively monomers from the red branches of the phylogenetic tree.

CABD02378856.1_1
CABD02378856.1_2
CABD02378856.1_3
CABD02378856.1_4
CABD02378856.1_5
CABD02378856.1_6
CABD02378856.1_7
CABD02378856.1_8
CABD02378856.1_9
CABD02378856.1_10
CABD02378856.1_11
CABD02378856.1_12
CABD02378856.1_13
CABD02378856.1_14
CABD02378856.1_15
CABD02378856.1_16
CABD02378856.1_17
CABD02378856.1_18
CABD02378856.1_19
CABD02378856.1_20
CABD02378856.1_21
CABD02378856.1_22
CABD02378856.1_23
CABD02378856.1_24
CABD02378856.1_25
CABD02378856.1_26
CABD02378856.1_27
CABD02378856.1_28
CABD02378856.1_29
CABD02378856.1_30
CABD02378856.1_31
CABD02378856.1_32
CABD02378856.1_33
CABD02378856.1_34
CABD02378856.1_35
CABD02378856.1_36
CABD02378856.1_37
CABD02378856.1_38
CABD02378856.1_39
CABD02378856.1_40
CABD02378856.1_41
CABD02378856.1_42
CABD02378856.1_43
CABD02378856.1_44
CABD02378856.1_45
CABD02378856.1_46
CABD02378856.1_47
CABD02378856.1_48
CABD02378856.1_49
CABD02378856.1_50
CABD02378856.1_51
CABD02378856.1_52
CABD02378856.1_53
CABD02378856.1_54
CABD02378856.1_55
CABD02378856.1_56
CABD02378856.1_57
CABD02378856.1_58
CABD02378856.1_59
CABD02378856.1_60
CABD02378856.1_61
CABD02378856.1_62
CABD02378856.1_63
CABD02378856.1_64
CABD02378856.1_65
CABD02378856.1_66
CABD02378856.1_67
CABD02378856.1_68
CABD02378856.1_69
CABD02378856.1_70
CABD02378856.1_71
CABD02378856.1_72
CABD02378856.1_73
CABD02378856.1_74
CABD02378856.1_75
CABD02378856.1_76
CABD02378856.1_77
CABD02378856.1_78
CABD02378856.1_79
CABD02378856.1_80
CABD02378856.1_81
CABD02378856.1_82
CABD02378856.1_83
CABD02378856.1_84
CABD02378856.1_85
CABD02378856.1_86
CABD02378856.1_87
CABD02378856.1_88
CABD02378856.1_89
CABD02378856.1_90
CABD02378856.1_91
CABD02378856.1_92
CABD02378856.1_93
CABD02378856.1_94
CABD02378856.1_95
CABD02378856.1_96
CABD02378856.1_97
CABD02378856.1_98

CABD02339997.1_1
CABD02339997.1_2
CABD02339997.1_3
CABD02339997.1_4
CABD02339997.1_5
CABD02339997.1_6
CABD02339997.1_7
CABD02339997.1_8
CABD02219203.1_1
CABD02219203.1_2
CABD02219203.1_3
CABD02219203.1_4
CABD02219203.1_5
CABD02219203.1_6
CABD02219203.1_7
CABD02219203.1_8
CABD02219203.1_9
CABD02219203.1_10
CABD02219203.1_11
CABD02219203.1_12
CABD02219203.1_13
CABD02219203.1_14
CABD02219203.1_15
CABD02219203.1_16
CABD02219203.1_17
CABD02219203.1_18
CABD02219203.1_19
CABD02219203.1_20
CABD02219203.1_21
CABD02219203.1_22
CABD02219203.1_23
CABD02219203.1_24
CABD02219203.1_25
CABD02219203.1_26
CABD02219203.1_27
CABD02219203.1_28
CABD02219203.1_29
CABD02219203.1_30
CABD02219203.1_31
CABD02219203.1_32
CABD02219203.1_33
CABD02219203.1_34
CABD02219203.1_35
CABD02219203.1_36
CABD02219203.1_37
CABD02219203.1_38
CABD02219203.1_39
CABD02219203.1_40
CABD02219203.1_41
CABD02219203.1_42
CABD02219203.1_43
CABD02219203.1_44
CABD02219203.1_45
CABD02219203.1_46
CABD02219203.1_47
CABD02219203.1_48
CABD02219203.1_49
CABD02219203.1_50
CABD02219203.1_51
CABD02219203.1_52
CABD02219203.1_53
CABD02219203.1_54
CABD02219203.1_55
CABD02219203.1_56
CABD02219203.1_57

CABD02196967.1_1
CABD02196967.1_2
CABD02196967.1_3
CABD02196967.1_4
CABD02196967.1_5
CABD02196967.1_6
CABD02196967.1_7
CABD02196967.1_8
CABD02196967.1_9
CABD02196967.1_10
CABD02196967.1_11
CABD02196967.1_12
CABD02196967.1_13
CABD02196967.1_14
CABD02196967.1_15
CABD02196967.1_16
CABD02196967.1_17
CABD02196967.1_18
CABD02196967.1_19
CABD02196967.1_20
CABD02196967.1_21
CABD02196967.1_22
CABD02196967.1_23
CABD02196967.1_24
CABD02196967.1_25
CABD02196967.1_26
CABD02196967.1_27
CABD02196967.1_28
CABD02196967.1_29
CABD02196967.1_30
CABD02196967.1_31
CABD02196967.1_32
CABD02196967.1_33
CABD02196967.1_34
CABD02196967.1_35
CABD02196967.1_36
CABD02196967.1_37
CABD02196967.1_38
CABD02196967.1_39
CABD02196967.1_40
CABD02196967.1_41
CABD02196967.1_42
CABD02196967.1_43
CABD02196967.1_44
CABD02196967.1_45
CABD02196967.1_46
CABD02196967.1_47
CABD02196967.1_48

CABD02196940.1_1
CABD02196940.1_2
CABD02196940.1_3
CABD02196940.1_4
CABD02196940.1_5
CABD02196940.1_6
CABD02196940.1_7
CABD02196940.1_8
CABD02196940.1_9
CABD02196940.1_10
CABD02196940.1_11
CABD02196940.1_12
CABD02196940.1_13
CABD02196940.1_14
CABD02196940.1_15
CABD02196940.1_16
CABD02196940.1_17
CABD02196940.1_18
CABD02196940.1_19
CABD02196940.1_20
CABD02196940.1_21
CABD02196940.1_22
CABD02196940.1_23
CABD02196940.1_24
CABD02196940.1_25
CABD02196940.1_26
CABD02196940.1_27
CABD02196940.1_28
CABD02196940.1_29
CABD02196940.1_30
CABD02196940.1_31
CABD02196940.1_32
CABD02196940.1_33
CABD02196940.1_34
CABD02196940.1_35
CABD02196940.1_36
CABD02196940.1_37
CABD02196940.1_38
CABD02196940.1_39
CABD02196940.1_40
CABD02196940.1_41
CABD02196940.1_42
CABD02196940.1_43
CABD02196940.1_44
CABD02196940.1_45
CABD02196940.1_46
CABD02196940.1_47
CABD02196940.1_48
CABD02196940.1_49
CABD02196940.1_50
CABD02196940.1_51
CABD02196940.1_52
CABD02196940.1_53
CABD02196940.1_54
CABD02196940.1_55
CABD02196940.1_56
CABD02196940.1_57
CABD02196940.1_58
CABD02196940.1_59
CABD02196940.1_60
CABD02196940.1_61
CABD02196940.1_62
CABD02196940.1_63
CABD02196940.1_64
CABD02196940.1_65
CABD02196940.1_66
CABD02196940.1_67
CABD02196940.1_68
CABD02196940.1_69
CABD02196940.1_70
CABD02196940.1_71
CABD02196940.1_72
CABD02196940.1_73
CABD02196940.1_74
CABD02196940.1_75
CABD02196940.1_76
CABD02196940.1_77

CABD02196935.1_1
CABD02196935.1_2
CABD02196935.1_3
CABD02196935.1_4
CABD02196935.1_5
CABD02196935.1_6
CABD02196935.1_7
CABD02196935.1_8
CABD02196935.1_9
CABD02196935.1_10
CABD02196935.1_11
CABD02196935.1_12
CABD02196935.1_13
CABD02196935.1_14
CABD02196935.1_15
CABD02196935.1_16
CABD02196935.1_17
CABD02196935.1_18
CABD02196935.1_19
CABD02196935.1_20
CABD02196935.1_21
CABD02196935.1_22
CABD02196935.1_23
CABD02196935.1_24
CABD02196935.1_25
CABD02196935.1_26
CABD02196935.1_27
CABD02196935.1_28
CABD02196935.1_29
CABD02196935.1_30
CABD02196935.1_31
CABD02196935.1_32
CABD02196935.1_33
CABD02196935.1_34
CABD02196935.1_35
CABD02196935.1_36
CABD02196935.1_37
CABD02196935.1_38
CABD02196935.1_39
CABD02196935.1_40
CABD02196935.1_41
CABD02196935.1_42
CABD02196935.1_43
CABD02196935.1_44
CABD02196935.1_45
CABD02196935.1_46
CABD02196935.1_47
CABD02196935.1_48
CABD02196935.1_49
CABD02196935.1_50
CABD02196935.1_51
CABD02196935.1_52
CABD02196935.1_53
CABD02196935.1_54
CABD02196935.1_55
CABD02196935.1_56
CABD02196935.1_57
CABD02196935.1_58
CABD02196935.1_59
CABD02196935.1_60
CABD02196935.1_61
CABD02196935.1_62
CABD02196935.1_63
CABD02196935.1_64
CABD02196935.1_65
CABD02196935.1_66
CABD02196935.1_67
CABD02196935.1_68
CABD02196935.1_69
CABD02196935.1_70
CABD02196935.1_71
CABD02196935.1_72
CABD02196935.1_73
CABD02196935.1_74
CABD02196935.1_75
CABD02196935.1_76
CABD02196935.1_77
CABD02196935.1_78
CABD02196935.1_79

CABD02162533.1_1
CABD02162533.1_2
CABD02162533.1_3
CABD02162533.1_4
CABD02162533.1_5
CABD02162533.1_6
CABD02162533.1_7
CABD02162533.1_8
CABD02162533.1_9
CABD02162533.1_10
CABD02162533.1_11
CABD02162533.1_12
CABD02162533.1_13

CABD02162145.1_1
CABD02162145.1_2
CABD02162145.1_3
CABD02162145.1_4
CABD02162145.1_5
CABD02162145.1_6
CABD02162145.1_7
CABD02162145.1_8
CABD02162145.1_9
CABD02162145.1_10
CABD02162145.1_11
CABD02162145.1_12
CABD02162145.1_13
CABD02162145.1_14
CABD02162145.1_15
CABD02162145.1_16

CABD02111890.1_1
CABD02111890.1_2
CABD02111890.1_3
CABD02111890.1_4
CABD02111890.1_5
CABD02111890.1_6
CABD02111890.1_7
CABD02111890.1_8
CABD02111890.1_9
CABD02111890.1_10
CABD02111890.1_11
CABD02111890.1_12
CABD02111890.1_13
CABD02111890.1_14
CABD02111890.1_15
CABD02111890.1_16
CABD02111890.1_17
CABD02111890.1_18
CABD02111890.1_19
CABD02111890.1_20
CABD02111890.1_21
CABD02111890.1_22
CABD02111890.1_23
CABD02111890.1_24
CABD02111890.1_25
CABD02111890.1_26
CABD02111890.1_27
CABD02111890.1_28
CABD02111890.1_29
CABD02111890.1_30
CABD02111890.1_31
CABD02111890.1_32
CABD02111890.1_33
CABD02111890.1_34
CABD02111890.1_35
CABD02111890.1_36
CABD02111890.1_37
CABD02111890.1_38
CABD02111890.1_39
CABD02111890.1_40
CABD02111890.1_41
CABD02111890.1_42
CABD02111890.1_43
CABD02111890.1_44
CABD02111890.1_45
CABD02111890.1_46
CABD02111890.1_47
CABD02111890.1_48
CABD02111890.1_49

CABD02105058.1_1
CABD02105058.1_2
CABD02105058.1_3
CABD02105058.1_4
CABD02105058.1_5
CABD02105058.1_6
CABD02105058.1_7
CABD02105058.1_8
CABD02105058.1_9
CABD02105058.1_10
CABD02105058.1_11
CABD02105058.1_12
CABD02105058.1_13
CABD02105058.1_14
CABD02105058.1_15
CABD02105058.1_16
CABD02105058.1_17
CABD02105058.1_18
CABD02105058.1_19
CABD02105058.1_20
CABD02105058.1_21
CABD02105058.1_22
CABD02105058.1_23
CABD02105058.1_24
CABD02105058.1_25
CABD02105058.1_26
CABD02105058.1_27
CABD02105058.1_28
CABD02105058.1_29
CABD02105058.1_30
CABD02105058.1_31
CABD02105058.1_32
CABD02105058.1_33
CABD02105058.1_34
CABD02105058.1_35
CABD02105058.1_36
CABD02105058.1_37
CABD02105058.1_38
CABD02105058.1_39
CABD02105058.1_40
CABD02105058.1_41
CABD02105058.1_42
CABD02105058.1_43
CABD02105058.1_44
CABD02105058.1_45
CABD02105058.1_46
CABD02105058.1_47
CABD02105058.1_48
CABD02105058.1_49
CABD02105058.1_50
CABD02105058.1_51
CABD02105058.1_52
CABD02105058.1_53
CABD02105058.1_54
CABD02105058.1_55
CABD02105058.1_56
CABD02105058.1_57
CABD02105058.1_58
CABD02105058.1_59
CABD02105058.1_60
CABD02105058.1_61
CABD02105058.1_62
CABD02105058.1_63
CABD02105058.1_64
CABD02105058.1_65
CABD02105058.1_66
CABD02105058.1_67
CABD02105058.1_68
CABD02105058.1_69
CABD02105058.1_70
CABD02105058.1_71
CABD02105058.1_72
CABD02105058.1_73
CABD02105058.1_74
CABD02105058.1_75
CABD02105058.1_76
CABD02105058.1_77
CABD02105058.1_78
CABD02105058.1_79
CABD02105058.1_80
CABD02105058.1_81
CABD02105058.1_82
CABD02105058.1_83
CABD02105058.1_84
CABD02105058.1_85
CABD02105058.1_86
CABD02105058.1_87

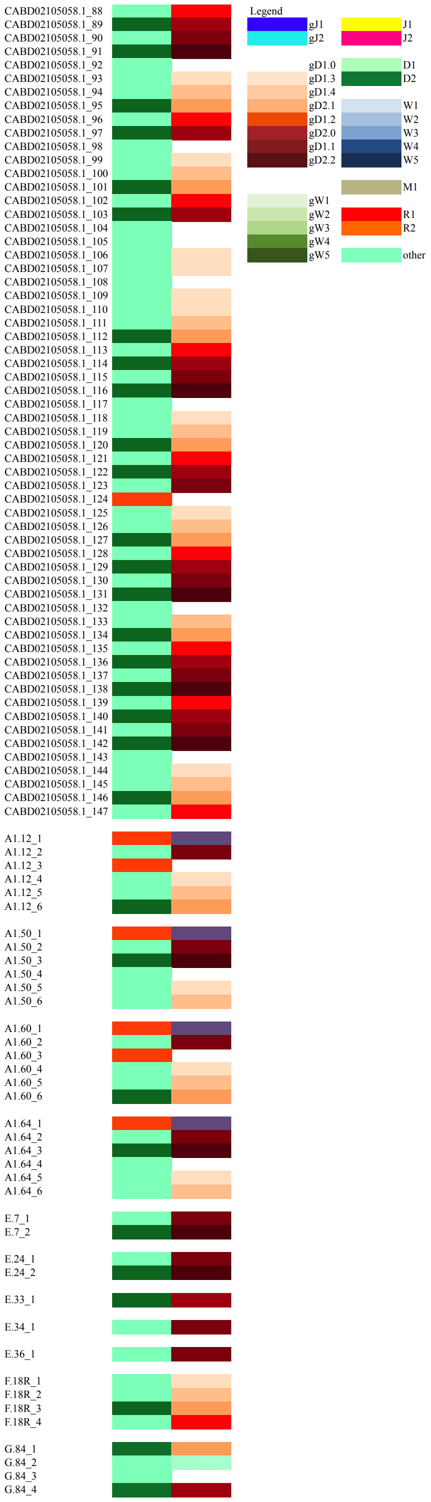
CABD02105058.1_88
CABD02105058.1_89
CABD02105058.1_90
CABD02105058.1_91
CABD02105058.1_92
CABD02105058.1_93
CABD02105058.1_94
CABD02105058.1_95
CABD02105058.1_96
CABD02105058.1_97
CABD02105058.1_98
CABD02105058.1_99
CABD02105058.1_100
CABD02105058.1_101
CABD02105058.1_102
CABD02105058.1_103
CABD02105058.1_104
CABD02105058.1_105
CABD02105058.1_106
CABD02105058.1_107
CABD02105058.1_108
CABD02105058.1_109
CABD02105058.1_110
CABD02105058.1_111
CABD02105058.1_112
CABD02105058.1_113
CABD02105058.1_114
CABD02105058.1_115
CABD02105058.1_116
CABD02105058.1_117
CABD02105058.1_118
CABD02105058.1_119
CABD02105058.1_120
CABD02105058.1_121
CABD02105058.1_122
CABD02105058.1_123
CABD02105058.1_124
CABD02105058.1_125
CABD02105058.1_126
CABD02105058.1_127
CABD02105058.1_128
CABD02105058.1_129
CABD02105058.1_130
CABD02105058.1_131
CABD02105058.1_132
CABD02105058.1_133
CABD02105058.1_134
CABD02105058.1_135
CABD02105058.1_136
CABD02105058.1_137
CABD02105058.1_138
CABD02105058.1_139
CABD02105058.1_140
CABD02105058.1_141
CABD02105058.1_142
CABD02105058.1_143
CABD02105058.1_144
CABD02105058.1_145
CABD02105058.1_146
CABD02105058.1_147
A1.12_1
A1.12_2
A1.12_3
A1.12_4
A1.12_5
A1.12_6
A1.50_1
A1.50_2
A1.50_3
A1.50_4
A1.50_5
A1.50_6
A1.60_1
A1.60_2
A1.60_3
A1.60_4
A1.60_5
A1.60_6
A1.64_1
A1.64_2
A1.64_3
A1.64_4
A1.64_5
A1.64_6
E.7_1
E.7_2
E.24_1
E.24_2
E.33_1
E.34_1
E.36_1
F.18R_1
F.18R_2
F.18R_3
F.18R_4
G.84_1
G.84_2
G.84_3
G.84_4
Legend
gJ1
gJ2
gD1.0
gD1.3
gD1.4
gD2.1
gD1.2
gD2.0
gD1.1
gD2.2
gW1
gW2
gW3
gW4
gW5
J1
J2
D1
D2
W1
W2
W3
W4
W5
M1
R1
R2
other

Supporting Note Table 9. Alphoid composition and organiation of the WGSSs and plasmids (5+1) containing exclusively monomers from the green branches of the phylogenetic tre

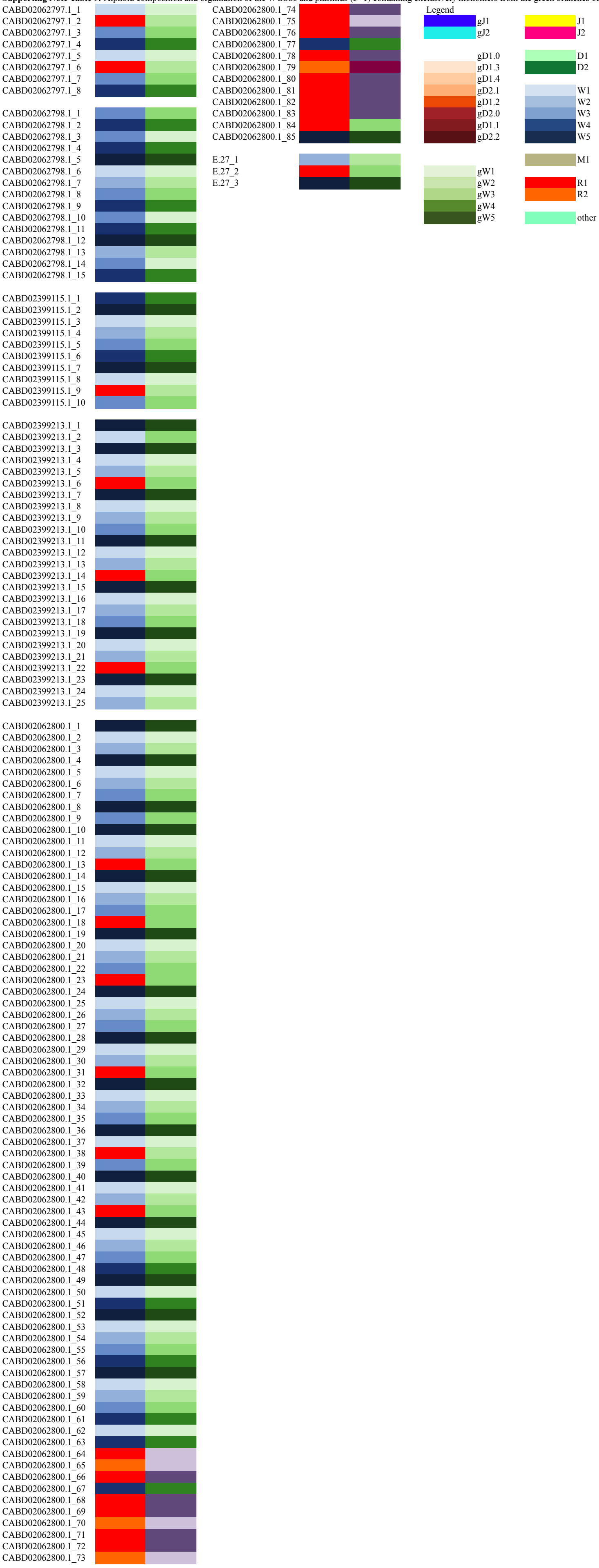

Supplement: Supplementary Information [file srep14189-s1.pdf]
